# Phytochemical Analysis and Bioevaluation of *Echinophora sibthorpiana* Guss. (Apiaceae): Antioxidant Capacity, DNA Protection and In Vivo Genotoxic Safety

**DOI:** 10.3390/antiox15081009

**Published:** 2026-08-13

**Authors:** Seren Gündoğdu, Gülnur Ipek Erdemli, Merve Yüzbaşıoğlu Baran, Güzin Emecen, Aslı Doğru, Emirhan Nemutlu, András Simon, Ayşe Kuruüzüm-Uz

**Affiliations:** 1 Department of Pharmacognosy, Faculty of Pharmacy, Hacettepe University, 06100 Ankara, Türkiye; 2 Functional and Evolutionary Genetics Laboratory, Department of Biology, Faculty of Science, Hacettepe University, 06800 Ankara, Türkiye; gulnur.ipek11@hacettepe.edu.tr (G.I.E.); guzin@hacettepe.edu.tr (G.E.); 3 Department of Pharmacognosy, Gülhane Faculty of Pharmacy, University of Health Sciences, 06108 Ankara, Türkiye; merve.yuzbasioglu@sbu.edu.tr; 4 Division of Biology, Department of Botany, Faculty of Science, Hacettepe University, 06800 Ankara, Türkiye; adogru@hacettepe.edu.tr; 5 Department of Analytical Chemistry, Faculty of Pharmacy, Hacettepe University, 06100 Ankara, Türkiye; enemutlu@hacettepe.edu.tr; 6 Department of Inorganic and Analytical Chemistry, Faculty of Chemical Technology and Biotechnology, Budapest University of Technology and Economics, Szt. Gellért tér 4, H-1111 Budapest, Hungary; simon.andras@vbk.bme.hu

**Keywords:** *Echinophora sibthorpiana*, Apiaceae, flavonoid glycosides, fingerprint analysis, antioxidant activity, DNA protection, genotoxicity, antigenotoxicity, *Drosophila melanogaster*, SMART assay

## Abstract

*Echinophora sibthorpiana* Guss. (Apiaceae) is an aromatic plant of considerable ethnobotanical significance, traditionally employed as a flavouring and preservative agent in food and valued for its medicinal properties across the Eastern Mediterranean region, yet its non-volatile phytochemistry and biosafety profile remain insufficiently characterized. In the present study, the *n*-butanol fraction of the 80% methanolic extract of the aerial parts of *E. sibthorpiana* was fractionated using chromatographic methods and the structures of the isolated compounds were elucidated by 1D and 2D-NMR spectroscopy and HR-ESI-MS. The distribution of *Echinophora* metabolites isolated by our group across six species in Türkiye was assessed by LC-qTOF-MS. Antioxidant capacity was evaluated by CUPRAC, FRAP, and TEAC assays; DNA-protective activity by the pBR322 plasmid model; genotoxic and antigenotoxic potential in the *Drosophila melanogaster* wing SMART assay. Four secondary metabolites, known as vicenin-2 (**1**), rutin (**2**), isoquercitrin (**3**) and betulalbuside A (**4**), were isolated and reported from *E. sibthorpiana* for the first time. Notably, the C-glycoside flavone vicenin-2 and the acyclic monoterpene glucoside betulalbuside A also represent the first isolation of these compounds from the genus *Echinophora*. LC-qTOF-MS profiling identified widely distributed flavonoid constituents together with more restricted metabolites that may have chemotaxonomic relevance. The *n*-BuOH fraction and the flavonol glycosides rutin and isoquercitrin exhibited the highest antioxidant and DNA-protective activities. None of the tested samples displayed genotoxic activity, while all showed antigenotoxic effects against ethyl methanesulfonate-induced DNA damage, with total spot frequency inhibition ranging from 47% to 83%. Overall, these findings provide a first comprehensive characterization of the non-volatile phytochemistry and biosafety profile of *E. sibthorpiana* and support the further investigation of its constituents as safe antioxidant and chemopreventive agents with potential nutraceutical applications.

## 1. Introduction

Plants have constituted a primary source of medicines and functional foods across human cultures since antiquity. The isolation of pharmacologically active compounds from medicinal plants, including morphine, digitoxin, quinine, paclitaxel, artemisinin, and silymarin, represents one of the most significant milestones in the history of medicine [1]. According to the World Health Organization (WHO), approximately 80% of the global population relies on plant-based preparations as a first line of healthcare, while plant-derived compounds account for about 25% of all drugs approved by the U.S. Food and Drug Administration (FDA) and the European Medicines Agency (EMA) [2,3]. The adverse effects associated with many synthetic drugs, combined with the extent of unexplored plant biodiversity, continues to sustain the relevance of natural products as a source of molecular scaffolds for drug discovery [4]. Accordingly, increasing attention has been directed toward evaluating the genotoxic and antigenotoxic potential of plant-derived compounds, as phytochemicals may either protect against or promote DNA damage depending on their chemical structure, concentration and biological context [5,6].

The family Apiaceae (Umbelliferae), comprising approximately 300–455 genera and 3000–3750 species distributed predominantly across the Northern Hemisphere temperate zone [7], is among the largest and most economically significant families of flowering plants. The genus *Echinophora* Tourn. ex L., a member of this family, encompasses approximately eleven taxa globally and is represented by three species in Europe: *E. spinosa* L., *E. sibthorpiana* Guss., and *E. tenuifolia* L. In Türkiye, six species occur, three of which are endemic: *E. chrysantha* Freyn & Sint., *E. lamondiana* Yıldız & Z. Bahçecioğlu, and *E. trichophylla* Sm.; the non-endemic members are *E. orientalis* Hedge & Lamond, *E. sibthorpiana* Guss., and *E. tournefortii* Jaub. & Spach. In Iran, five species are recorded, including two endemics, *E. platyloba* DC. and *E. cinerea* (Boiss.) Hedge & Lamond [8,9,10,11]. *Echinophora* species are aromatic plants with a long history of use as flavouring and preservative agents in food (particularly in Türkiye, Greece, and Iran) and have been employed in folk medicine for their antifungal, carminative, digestive, and wound-healing properties [12,13,14]. Despite their long-standing ethnobotanical use, the phytochemistry, and pharmacology of the genus remain comparatively underexplored.

Most published phytochemical studies on *Echinophora* genus have focused on essential oils, in which monoterpene hydrocarbons (principally α-phellandrene, δ-3-carene, and β-phellandrene) and the phenylpropanoid methyl eugenol predominate [15,16,17]. In contrast, information on non-volatile metabolites is comparatively limited. These metabolites include phenolic acids, flavonoids (rutin, quercitrin, and hesperidin), coumarins, saponins, polyacetylenes, fatty acids, phytosterols, and terpenoids [18,19,20,21,22]. Among these, the genus-specific C14 polyacetylenes (echinophorins A, B, and D), isolated from *E. platyloba* and *E. cinerea*, are of particular pharmacological interest owing to their selective modulation of the TRPA1 channel, an ion channel implicated in neuropathic and inflammatory pain signalling [23]. Biological activities documented across the genus include antioxidant, antifungal, anti-inflammatory, cytotoxic, and antimutagenic properties [24,25,26].

*Echinophora sibthorpiana* Guss. (syn. *E. tenuifolia* L. subsp. *sibthorpiana* (Guss.) Tutin) is the most widely studied member of the genus. The species is native to southeastern Europe, Crimea, western and Central Asia, and northeastern Afghanistan [11], it is only *Echinophora* species occurring in Bulgarian flora, and one of six species recorded in Türkiye. Currently accepted under this binomial name by Plants of the World Online (Royal Botanic Gardens, Kew), it is known locally in Türkiye as “çörtük” or “tarhana otu”, names that reflect its principal culinary roles as a spice in meats, pickles, and dairy products, as well as an ingredient of tarhana, a traditional fermented cereal food. The addition of the plant to tarhana has been shown to sustain lactic acid bacteria and yeast populations throughout fermentation, thereby positively affecting the nutritional profile of the final product [27,28]. In folk medicine, the species is valued for antifungal, wound-healing, and digestive properties [12].

Phytochemical studies on *E. sibthorpiana* have revealed considerable compositional variability, most notably within the essential oil. GC-MS analyses of material from Turkish, Greek, Bulgarian, and Iranian origins consistently identify α-phellandrene, δ-3-carene, and methyl eugenol as major constituents, yet their relative proportions differ markedly between collections. Baser et al. reported α-phellandrene as the dominant compound in Turkish material (51.0%) [15], whereas Chalchat et al. found δ-3-carene predominating (36.6–38.8%) in material from Konya [29], and Ahmad et al. identified methyl eugenol as the principal constituent (50.4%) in Iranian collections [30]. A comparison of wild and cultivated Turkish populations further demonstrated significant differences in both oil composition and antifungal efficacy [31]. These discrepancies are attributable to geographic, agronomic, and phenological variables, underscoring the necessity of chemotype-specific characterization prior to any standardized application of the plant material. With respect to non-volatile constituents, the first systematic phenolic characterization of the species documented high total phenolic content (111.3 ± 9.1 mg GAE/g dry extract), with rutin as the predominant individual compound (29.625 mg/g), alongside chlorogenic, ferulic, rosmarinic, and *p*-coumaric acids, and hesperidin [32]. Reported biological activities encompass antimicrobial effects against foodborne pathogens and phytopathogenic fungi [12,31], antioxidant and radical scavenging capacity [32], and, most recently, alleviation of glucose-induced endoplasmic reticulum and mitochondrial stress in a *Caenorhabditis elegans* model [33].

Whether a plant-derived compound damages DNA or protects the genome against chemically induced injury depends on its chemical structure, concentration, exposure duration, metabolism, and the biological system used for evaluation. Phytochemicals may display genotoxic, neutral, or antigenotoxic effects depending on these variables. For this reason, plant extracts and isolated natural products intended for pharmaceutical, nutraceutical, cosmetic, or food-related applications require systematic genetic safety evaluation before their biological activity can be interpreted as beneficial. The increasing use of plant-derived antioxidants and chemopreventive candidates has created a need for experimental systems that can distinguish intrinsic genotoxicity from DNA-protective activity. *Drosophila melanogaster* is widely used for this purpose because of its short life cycle, low maintenance cost, extensive genetic characterization, conserved physiological and metabolic features make *D. melanogaster* useful for preliminary toxicological and nutrigenomic evaluations [34,35,36].

Among the available *Drosophila*-based genotoxicity assays, the wing Somatic Mutation and Recombination Test (SMART), occupies a central position. The assay was developed to detect somatic mutation and mitotic recombination events in wing imaginal disc cells of *D. melanogaster* larvae [6]. Because the adult wing preserves clonal genetic alterations that arise during larval development, the test permits the scoring of small single spots, large single spots, and twin spots, thereby allowing mutation- and recombination-related endpoints to be evaluated within a single in vivo assay [6,37]. SMART is particularly suitable for screening plant extracts and isolated phytochemicals because it can detect both directly acting genotoxic agents and compounds requiring metabolic activation. In addition to the standard cross, high-bioactivation crosses have also been developed to increase the sensitivity of the assay for detecting promutagens requiring metabolic activation [38]. For this reason, SMART has been used in studies evaluating food dyes, plant-derived compounds, essential oil constituents, heavy metals, and other environmental or pharmacologically relevant agents [39,40,41,42].

Despite these advances, the genotoxic safety profile of *Echinophora* extracts and isolated compounds, as well as their antioxidant potentials, remains insufficiently characterized. In the present study, secondary metabolites from *E. sibthorpiana* were isolated and structurally elucidated by high-resolution quadrupole time-of-flight mass spectrometry (qTOF-MS) and 1D/2D NMR spectroscopy. In addition, their distribution across six *Echinophora* species native to Türkiye was investigated by LC-qTOF-MS. Antioxidant capacity and DNA-protective activity were assessed through in vitro radical scavenging and plasmid DNA protection assays. The genotoxic and antigenotoxic potential of the extracts and isolated compounds was further evaluated using the *D. melanogaster* wing SMART assay. These findings provide both a comprehensive phytochemical characterization and a biosafety assessment of this ethnobotanically important species.

## 2. Materials and Methods

### 2.1. Plant Materials

**Figure 1:**
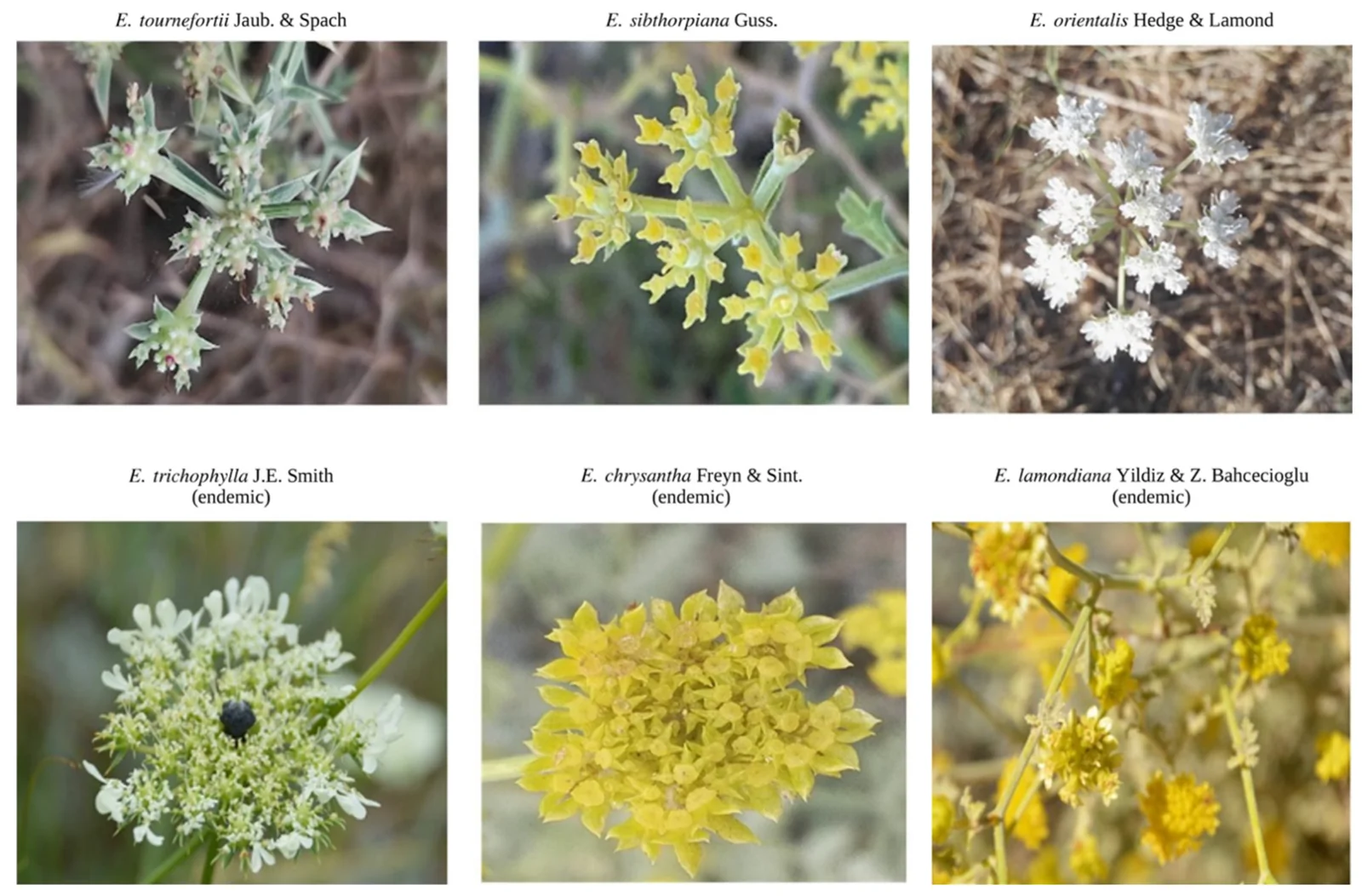
*Echinophora* species growing in the Flora of Türkiye.

Six species of the genus *Echinophora* Tourn. ex L. were collected from the localities listed in Table 1. All specimens (Figure 1) were collected and identified by Assoc. Prof. Dr. Golshan Zare and Prof. Dr. Aslı Doğru in TÜBİTAK project (216Z034). Voucher specimens are deposited in the Herbarium of Hacettepe University, Faculty of Pharmacy, Ankara, Türkiye (HUEF). Collection details are provided in Table 1.

### 2.2. General Experimental Procedures

NMR spectra were recorded on a Bruker Avance III spectrometer (Bruker, Rheinstetten, Germany) using tetramethylsilane (TMS) as the internal standard. One- and two-dimensional NMR experiments included ^1^H, ^13^C, DEPT, ^1^H-^1^H COSY, HSQC, HMBC, NOESY and ROESY. High-resolution electrospray ionization mass spectrometry (HR-ESI-MS) measurements were carried out on an Agilent Q-TOF mass spectrometer (Agilent Technologies, Redwood City, CA, USA). Column chromatographic separations employed silica gel (200–300 mesh) and reversed-phase C_18_ silica gel (Merck, Darmstadt, Germany), and Sephadex LH-20 (Cytiva, Uppsala, Sweden) as stationary phases.

### 2.3. Extraction and Isolation

Isolation studies were conducted exclusively on *E. sibthorpiana*. Dried aerial parts (340 g) of this species were extracted by maceration at 40 °C in a large-capacity rotary evaporator for 3 × 3 h using a methanol-water mixture (MeOH:H_2_O, 80:20, *v*/*v*; 3 × 2.5 L), yielding 65.12 g of crude aqueous methanolic extract. For the remaining *Echinophora* species included in the biological activity studies, dried aerial parts (30 g each) were extracted with MeOH:H_2_O (80:20, *v*/*v*, 200 mL) at 45 °C for 3 h. All extracts were concentrated under reduced pressure and lyophilized to dryness. Extraction yields were 24.3% (*E. tournefortii*), 12.7% (*E. orientalis*), 21.8% (*E. trichophylla*), 21.8% (*E. chrysantha*), and 20.2% (*E. lamondiana*).

The crude extract of *E. sibthorpiana* (65.12 g) were dissolved in distilled water and successively partitioned with *n*-hexane and *n*-BuOH, yielding 4.05 g and 16.23 g of the respective fractions. The obtained extract was concentrated to dryness and then lyophilized. The *n*-BuOH soluble fraction (15 g) of *E. sibthorpiana* was dissolved in a small volume of distilled water and subjected to vacuum liquid chromatography (VLC) on LiChroprep C_18_ using a stepwise H_2_O-MeOH gradient (0, 20, 40, 60, 80, and 100% MeOH), affording ten primary fractions: Fr. 0–1 (4.3 g), Fr. 2–6 (0.8 g), Fr. 7–9 (1.2 g), Fr. 10–11 (0.9 g), Fr. 12–14 (3.2 g), Fr. 15–20 (3.1 g), Fr. 21–23 (0.7 g), Fr. 24–25 (0.8 g), Fr. 26–30 (0.6 g), and Fr. 31 (0.02 g).

During the isolation process, the TLC profiles of all fractions were compared with those of compounds previously isolated from *E. tournefortii*. Fractions matching known compounds were identified accordingly, whereas fractions displaying distinct chromatographic profiles were selected for further purification to isolate potentially unique constituents of *E. sibthorpiana*.

Fraction 10–11 (0.9 g) was applied to a Sephadex LH-20 column using 100% MeOH as the eluent. Sub-fraction Fr. 29–33 (126 mg) was subsequently purified by Medium-Pressure Liquid Chromatography (MPLC) with a stepwise gradient from 100% H_2_O to 100% MeOH. Fr. 74–78 (54.5 mg) and Fr. 79–82 (12.7 mg) were each further purified on a Sephadex LH-20 column (100% MeOH), yielding compound **1** (11.3 mg) and additional 5.1 mg of compound **1**, respectively. Fr. 13–27 (52.7 mg) from the same RP-VLC separation, was re-chromatographed on a VLC column using an H_2_O:MeOH gradient (100% H_2_O → 95% MeOH), affording a further 10.6 mg of compound **1**. Thus, compound **1** was isolated in a total yield of 26 mg from three independent purification pathways.

Fraction 12–14 (3.2 g) was chromatographed on a Sephadex LH-20 column using 100% MeOH, yielding several sub-fractions. Fr. 22–28 (1.16 g) was subjected to a second Sephadex LH-20 column under the same conditions, affording compound **2** (355 mg) and compound **3** (21.1 mg). Fr. 9–13 (361 mg), collected from the same initial Sephadex LH-20 fractionation, was purified by silica gel column chromatography using a CH_2_Cl_2_:MeOH:H_2_O gradient (90:10:1 → 60:40:4). The resulting sub-fractions were further purified by preparative thin-layer chromatography (prep-TLC) using an CH_2_Cl_2_:MeOH:H_2_O (61:32:7) solvent system to yield compound **4** (41.6 mg) in pure form.

### 2.4. LC-qTOF-MS-Based Dereplication of Isolated Phytochemicals in Echinophora Species

The occurrence of the pure compounds isolated from *E. sibthorpiana* in the present study (compounds **1**–**4**: vicenin-2, rutin, isoquercitrin and betulalbuside A), together with seven compounds previously isolated from *E. tournefortii* in our earlier study (echinophoroside A, echinophoroside B, echinophoroside C, amburoside A, amburoside B, 3′-demethoxy-amburoside B, and 4′-hydroxy-salviifoside A), was investigated in the methanolic extracts of six *Echinophora* species native to Türkiye (*E. tournefortii*, *E. sibthorpiana*, *E. lamondiana*, *E. orientalis*, *E. chrysantha*, and *E. trichophylla*) using LC-qTOF-MS.

Lyophilized plant extracts were reconstituted in acetonitrile:water (1:1, *v*/*v*) to final concentration of 1 mg mL^−1^ and injected directly into the LC-qTOF-MS system. The isolated pure compounds were initially dissolved in methanol (1 mg mL^−1^) and subsequently diluted with acetonitrile (1:1, *v*/*v*) to a final concentration of 1 µg mL^−1^ before analysis.

Chromatographic separation was performed on a C18 column (100 mm × 2.1 mm, 2.7 µm) at a flow rate of 0.3 mL min^−1^ using a binary mobile phase consisting of 0.1% formic acid in water (A) and 0.1% formic acid in acetonitrile (B). The gradient elution program was as follows: 10% B (0–1 min), increased linearly to 90% B (7 min), held at 90% B until 10 min, returned to 10% B by 11 min, and re-equilibrated at 10% B until 15 min. Mass spectrometric detection was carried out in negative electrospray ionization mode. Instrument parameters were identical to those described in our previously published method [43]. Chromatographic data were processed using Agilent MassHunter software (version B.07) for peak deconvolution and feature extraction.

### 2.5. Determination of Total Phenolic Content

Stock solutions of each extract were prepared at 1000 µg mL^−1^, and gallic acid standard solutions were prepared by serial dilution to yield final concentrations ranging from 50 to 400 µg mL^−1^. For the colorimetric assay, 10 µL of each solution was transferred to the wells of a 96-well microplate. Folin–Ciocalteu reagent (150 µL), pre-diluted 1:4 (*v*/*v*) with distilled water, was then added, followed by 50 µL of saturated aqueous sodium carbonate (Na_2_CO_3_) solution. Reaction mixtures were incubated at room temperature for 2 h, and absorbance was measured at 725 nm. Total phenolic content (TPC) was calculated from the gallic acid calibration curve and expressed as milligrams of gallic acid equivalents per gram of dry extract (mg GAE g^−1^). Data are presented as the mean ± standard deviation (SD) of three independent replicates (*n* = 3) [44].

### 2.6. Antioxidant Activity

#### 2.6.1. CUPRAC

Sample stock solutions were prepared at 1000 µg mL^−1^. Aliquots of 25 µL of each extract or standard solution were dispensed into 96-well microplate wells. Copper (II) chloride solution (50 µL), neocuproine (2,9-dimethyl-1,10-phenanthroline; 50 µL), and ammonium acetate buffer (50 µL; pH 7.0) were added sequentially, after which the volume was brought to the final reaction volume with 25 µL of distilled water. After incubation in the dark for 30 min, absorbance was measured at 450 nm. Results were expressed as mg GAE g^−1^ extract and reported as the mean ± SD (*n* = 3) [45].

#### 2.6.2. FRAP

Sample stock solutions were adjusted to 1000 µg mL^−1^, and Trolox calibration standards were prepared by serial dilution. Aliquots of 15 µL of each sample or Trolox standard were transferred to a 96-well microplate, followed by 285 µL of freshly prepared FRAP reagent. The reagent consisted of 2,4,6-tripyridyl-*s*-triazine (TPTZ; 2.5 mL, dissolved in 10 mM HCl), sodium acetate buffer (25 mL, pH 3.6), and iron (III) chloride hexahydrate solution (FeCl_3_·6H_2_O; 20 mL). Plates were incubated at 37 °C for 30 min, and absorbance was measured at 593 nm. Antioxidant capacity was calculated from the Trolox calibration curve and expressed as mg Trolox equivalents per gram of extract (mg TE g^−1^). Results are presented as mean ± SD (*n* = 3) [46].

#### 2.6.3. ABTS^•+^ Antioxidant Capacity Assay

2,2′-Azino-bis(3-ethylbenzothiazoline-6-sulfonic acid) diammonium salt (ABTS^•+^; 7 mM) and potassium persulfate (2.45 mM) were dissolved in distilled water and allowed to react for 16 h in the dark to generate the ABTS^•+^ radical cation solution. Before use, the stock solution was diluted with either ethanol or phosphate-buffered saline (pH 7.4) to obtain an absorbance of 0.700 ± 0.020 at 734 nm. Aliquots of 10 µL of each sample or Trolox standard solution were placed in a 96-well microplate, and 240 µL of the ABTS^•+^ working solution was added to each well. After 6 min at room temperature, absorbance was measured at 734 nm. Radical-scavenging activity was calculated from the inhibition of absorbance relative to Trolox and expressed as Trolox equivalent antioxidant capacity (TEAC, mg TE g^−1^). Data are expressed as the mean ± SD (*n* = 3) [47].

All antioxidant assays (including total phenolic content determinations) were performed in triplicate; that is, each assay was carried out as three independent experimental runs conducted under identical conditions, and the results are expressed as mean ± SD, (*n* = 3). Pearson correlation analysis was performed between the total phenolic content and the antioxidant capacities (CUPRAC, FRAP and TEAC) of the extracts using GraphPad Prism (version 11, GraphPad Software, San Diego, CA, USA). Correlations were considered statistically significant at *p* < 0.05.

### 2.7. Assessment of DNA-Protective Activity

#### 2.7.1. Protection of pBR322 Plasmid DNA Against H_2_O_2_/UV-Induced Damage by *E. sibthorpiana* Extracts

The ability of the *E. sibthorpiana* extract to prevent oxidative DNA strand scission was evaluated using the pBR322 plasmid DNA model (Thermo Scientific, Waltham, MA, USA, cat. no. SD0041), as described previously with minor modifications [48,49]. Reaction mixtures (10 µL total) contained 3 µL pBR322 DNA (172 ng µL^−1^ in phosphate-buffered saline, PBS), 1 µL of 30% (*v*/*v*) H_2_O_2_, and 5 µL of each extract solution. After irradiation at 302 nm (UV intensity: 8000 µW cm^−2^) for 5 min, samples were combined with 6× gel-loading dye and electrophoresed on 1% (*w*/*v*) agarose gels in 0.5× Tris-borate-EDTA (TBE) buffer at 90 V for 120 min. DNA bands were visualized by staining with SafeView™ (cat. no. G108, Applied Biological Materials, Richmond, BC, Canada) and imaged with a VisionWorks LS documentation system. Band intensities for the supercoiled (Form I) and open-circular (Form II) DNA were quantified by densitometry. Plasmid DNA was incubated with H_2_O_2_ under UV irradiation, either in the presence or absence of the test samples. The blank consisted of untreated plasmid DNA, corresponding to 100% of the supercoiled form (%RV = 100). The oxidative damage control consisted of plasmid DNA treated with H_2_O_2_ under UV irradiation in the absence of any extract or compound, representing the maximal level of oxidative DNA damage. The relative supercoiled DNA content (%RV) of each sample was calculated relative to the blank.

The proportion of supercoiled DNA (%S) and the relative protection value (%RS) were calculated as:

%S = [I_SC_/(I_SC_ + I_OC_)] × 100    %RS = %S(treated)/%S(control) × 100

where I_SC_ and I_OC_ represent the densitometric intensities of the supercoiled and open-circular forms, respectively. Statistical differences among groups were assessed by one-way analysis of variance (ANOVA) followed by Dunnett’s multiple comparison test (*p* < 0.01).

#### 2.7.2. DNA-Protective Activity of the *E. sibthorpiana* Extracts, *n*-BuOH Subfraction, and Isolated Compounds

The protective effects of the *E. sibthorpiana*-MeOH crude extract, its *n*-butanol-soluble subfraction (*n*-BuOH), and selected isolated compounds (each at 50 µg mL^−1^) against H_2_O_2_/UV-induced oxidative DNA damage were examined using the pBR322 plasmid model as described in Section 2.7.1. For crude extracts, the final assay concentration was 0.5 mg mL^−1^. Following UV irradiation (302 nm, 8000 µW cm^−2^, 5 min) at room temperature, samples were combined with 6× loading dye and electrophoresed on 1% agarose gels in 0.5× TBE at 90 V for 120 min. Staining with SafeView™ (G108), imaging, and densitometric calculation of %S and %RS were performed as described above (Section 2.7.1). Statistical analysis employed one-way ANOVA with Dunnett’s post-hoc test (*p* < 0.01).

### 2.8. Genotoxicity and Antigenotoxicity

#### 2.8.1. Somatic Mutation and Recombination Test-SMART Assay

Genotoxic and antigenotoxic activities were assessed using the wing Somatic Mutation and Recombination Test in *D. melanogaster*. The assay detects somatic genetic alterations such as gene mutations, chromosomal deletions, and mitotic recombination events occurring in proliferating wing imaginal disc cells during larval development [6,38]. Because SMART detects both mutational and recombinational endpoints, it provides a broader assessment of somatic genotoxic mechanisms than endpoint-specific assays.

The assay was conducted using trans-heterozygous larvae carrying the recessive wing hair markers *multiple wing hairs* (*mwh*) and *flare* (*flr*^3^). Mutant clones formed in the adult wing blade were interpreted as indicators of genetic alterations induced during larval development. The scoring criteria followed the standard SMART classification: small single spots, large single spots, twin spots, and total spots [6,38].

*Genetic crosses and rearing conditions*

Trans-heterozygous *mwh*/*flr*^3^ larvae were obtained by crossing *mwh*/*mwh* females with *flr*^3^/In (*3LR*) *TM3*, *Bd^S^* males. The genetic markers and chromosomal information for these stocks are described in *The Genome of D. melanogaster* [50]. All stocks and experimental cultures were maintained at 25 ± 1 °C and 60–70% relative humidity. Larvae were collected from synchronized egg-laying cultures. Third-instar larvae, 72 ± 4 h after oviposition, were used for treatment. Experimental groups were established using approximately 50 larvae per vial. Each vial contained 0.85 g of Instant *Drosophila* Medium rehydrated with 4 mL of the appropriate treatment solution.

#### 2.8.2. Genotoxicity and Antigenotoxicity Tests

The genotoxicity and antigenotoxicity assays were performed using the standard cross of the wing SMART assay in *Drosophila melanogaster*, following the procedure described by Frei and Würgler (1988) [51]. For the genotoxicity assay, larvae were exposed to *E. sibthorpiana* extracts or isolated compounds alone at two previously determined non-lethal concentrations.

The two concentrations tested for each extract and compound were determined by the amount of material available for the biological experiments. As the isolated compounds were obtained only in milligram-scale quantities typical of plant secondary metabolites (e.g., isoquercitrin, 21.1 mg; vicenin-2, 26 mg; betulalbuside A, 41.6 mg) [52], two feasible non-lethal concentrations were selected for each sample rather than a full dose series. Because the amount of material was limited, the genotoxicity (sample-alone) and antigenotoxicity (sample plus EMS) tests were run at the concentrations that remained available for each sample, which therefore differed slightly between samples and between the two assays. All concentrations were within non-lethal limits, as shown by the normal development of larvae into adults in every group, and are comparable to those used in earlier wing-spot studies of related flavonoids (e.g., vitexin at 0.15–0.6 mM) [53]; being a food/spice plant, these low, dietarily relevant concentrations are also physiologically appropriate [54].

For the antigenotoxicity assay, the same extracts and isolated compounds were co-administered with 1 mM ethyl methanesulfonate (EMS). EMS was used as a positive mutagenic control because it is a well-established alkylating agent in genotoxicity testing. Distilled water was used as both the negative and solvent control.

After adult emergence, flies were collected and preserved in 70% ethanol. Wings of trans-heterozygous adults were dissected, mounted in Faure’s solution, and examined under a compound microscope at 400× magnification. Mutant spots were scored as follows: small single spots, consisting of one or two mutant cells; large single spots, consisting of three or more mutant cells; twin spots, consisting of adjacent *mwh* and *flr*^3^ clones; and total spots, representing the sum of all spot categories. Each treatment was set up in three replicate vials, each containing approximately 50 larvae each, and 40 wings randomly selected from the emerged adults were analyzed per treatment group (80 wings for the EMS-treated positive control).

### 2.9. Statistical Analysis

Mutant spot frequencies in treated and control groups were compared using the conditional binomial test of Kastenbaum and Bowman (1970) [55]. Responses were classified as positive, weakly positive, negative, or inconclusive according to the multiple-decision procedure described by Frei and Würgler (1988) [51]. The Wilcoxon-Mann–Whitney U test was used as an additional non-parametric comparison when appropriate, following the statistical approach recommended for SMART data evaluation [56].

For genotoxicity experiments, each treatment group was compared with the negative control. For antigenotoxicity experiments, EMS + sample groups were compared with the EMS-only positive control. Statistical significance was accepted at *p* < 0.05.

## 3. Results

### 3.1. Characterization of the Isolated Compounds

**Figure 2:**
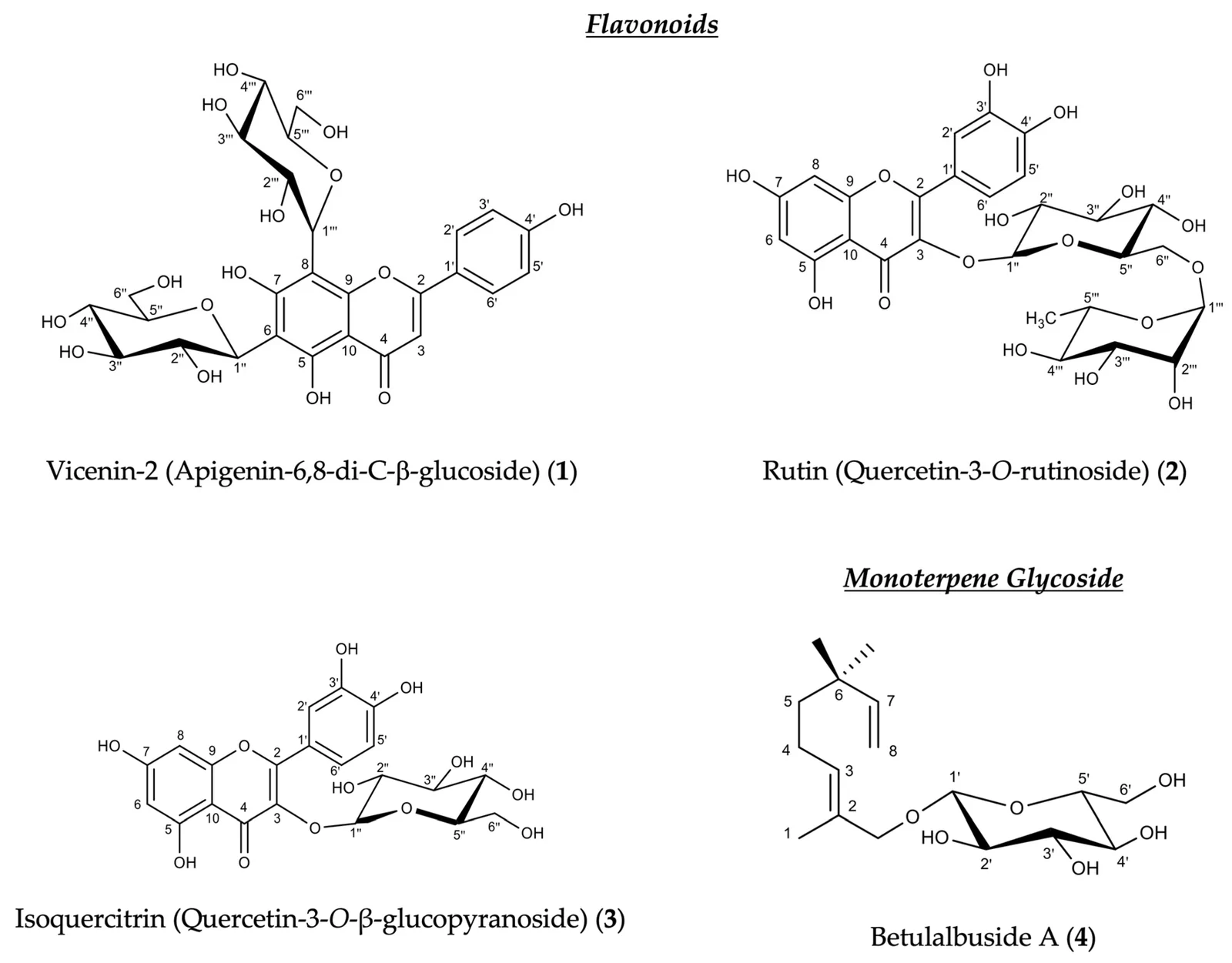
Chemical structures of compounds isolated from *E. sibthorpiana*.

The method for preparing the 80% MeOH extract from the aerial parts of *E. sibthorpiana* is described in detail in Section 2.3. The *n*-BuOH fraction of the 80% MeOH extract was subjected to various chromatographic methods. As a result, four compounds were isolated. The structures of the isolated compounds were determined by comparing 1D- and 2D-NMR (^1^H, ^13^C, DEPT, HMQC, HMBC, NOESY, ROESY) and HR-ESI-MS data (Supplementary File S1) with those reported in the literature. The compounds were identified as vicenin-2 (apigenin-6,8-di-C-glucoside) (**1**), rutin (quercetin-3-*O*-rutinoside) (**2**), isoquercitrin (quercetin-3-*O*-β-glucopyranoside) (**3**) and betulalbuside A (**4**) [57,58,59,60,61,62]. The chemical structures of all compounds isolated from *E. sibthorpiana* are presented in Figure 2.

### 3.2. LC-qTOF-MS-Based Phytochemical Profiling

**Figure 3:**
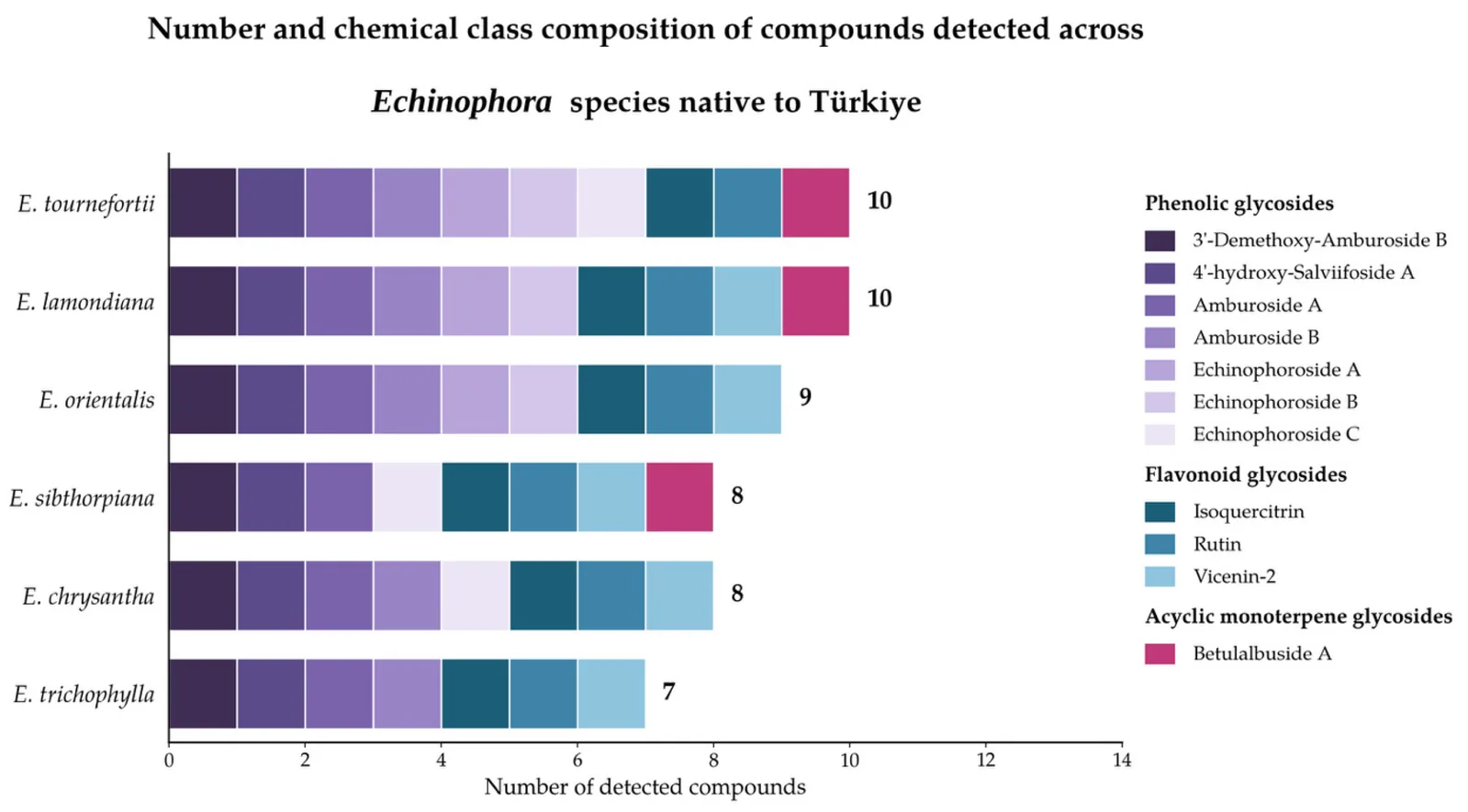
Distribution of target compounds across *Echinophora* species, as determined by LC-qTOF-MS-based extracted ion chromatogram (EIC) analysis.

The occurrence of the pure compounds isolated from E. *sibthorpiana* in the present study (compound **1**–**4**: vicenin-2, rutin, isoquercitrin and betulalbuside A), as well as that of seven compounds isolated from E. *tournefortii* in our other study [63] (echinophoroside A, echinophoroside B, echinophoroside C, amburoside A, amburoside B, 3′-demethoxy-amburoside B, and 4′-hydroxy-salviifoside A), was investigated in the methanolic extracts of six *Echinophora* species native to Türkiye (*E. tournefortii*, *E. sibthorpiana*, *E. lamondiana*, *E. orientalis*, *E. chrysantha*, and *E. trichophylla*) by LC-qTOF-MS in negative-ion mode. The presence or absence of each of the twelve target compounds in each species extract was assessed by extracted ion chromatogram (EIC) analysis based on the accurate *m*/*z* values of the deprotonated molecular ions ([M − H]^−^), verified by retention time matching against the corresponding isolated reference compounds (Figure 3, Supplementary File S2).

Among the twelve target compounds, rutin, isoquercitrin, amburoside A, 3′-demethoxy-amburoside B, and 4′-hydroxy-salviifoside A were detected in all six *Echinophora* species, indicating that these constituents represent conserved phytochemical constituents of the genus. Amburoside B and vicenin-2 showed a similarly broad distribution, being present in five of the six species, with amburoside B absent only in *E. sibthorpiana* and vicenin-2 absent only in *E. tournefortii*.

In contrast, the remaining compounds displayed a more restricted distribution. The monoterpene glycoside betulalbuside A were detected only in *E. tournefortii*, *E. sibthorpiana*, and *E. lamondiana*, and were absent in *E. orientalis*, *E. chrysantha*, and *E. trichophylla*. Echinophoroside C showed the most restricted distribution among the screened compounds, being detected exclusively in *E. tournefortii* and *E. sibthorpiana*. Echinophoroside B was detected in four species (*E. tournefortii*, *E. lamondiana*, *E. orientalis*, and *E. chrysantha*), being absent only in *E. sibthorpiana* and *E. trichophylla*. Echinophoroside A showed a more restricted distribution, being detected only in *E. tournefortii*, *E. lamondiana*, and *E. orientalis*, and absent in *E. sibthorpiana*, *E. chrysantha*, and *E. trichophylla*.

Overall, *E. tournefortii* and *E. lamondiana* displayed the broadest phytochemical profiles, each containing 11 of the 12 target compounds, whereas *E. trichophylla* displayed the most restricted profile, containing 7 of the 12 compounds. Notably, the widespread occurrence of rutin, isoquercitrin, and the three benzyl-glycoside-type compounds (amburoside A, 3′-demethoxy-amburoside B, and 4′-hydroxy-salviifoside A) across all examined species suggests that these constituents may serve as useful chemotaxonomic markers for the genus *Echinophora*, whereas the more restricted occurrence of betulalbuside A and echinophoroside C showed a more restricted distribution. These distribution patterns provide a basis for subsequent chemotaxonomic interpretation of the genus.

### 3.3. In Vitro Biological Activities

#### 3.3.1. Total Phenol Determination

**Figure 4:**
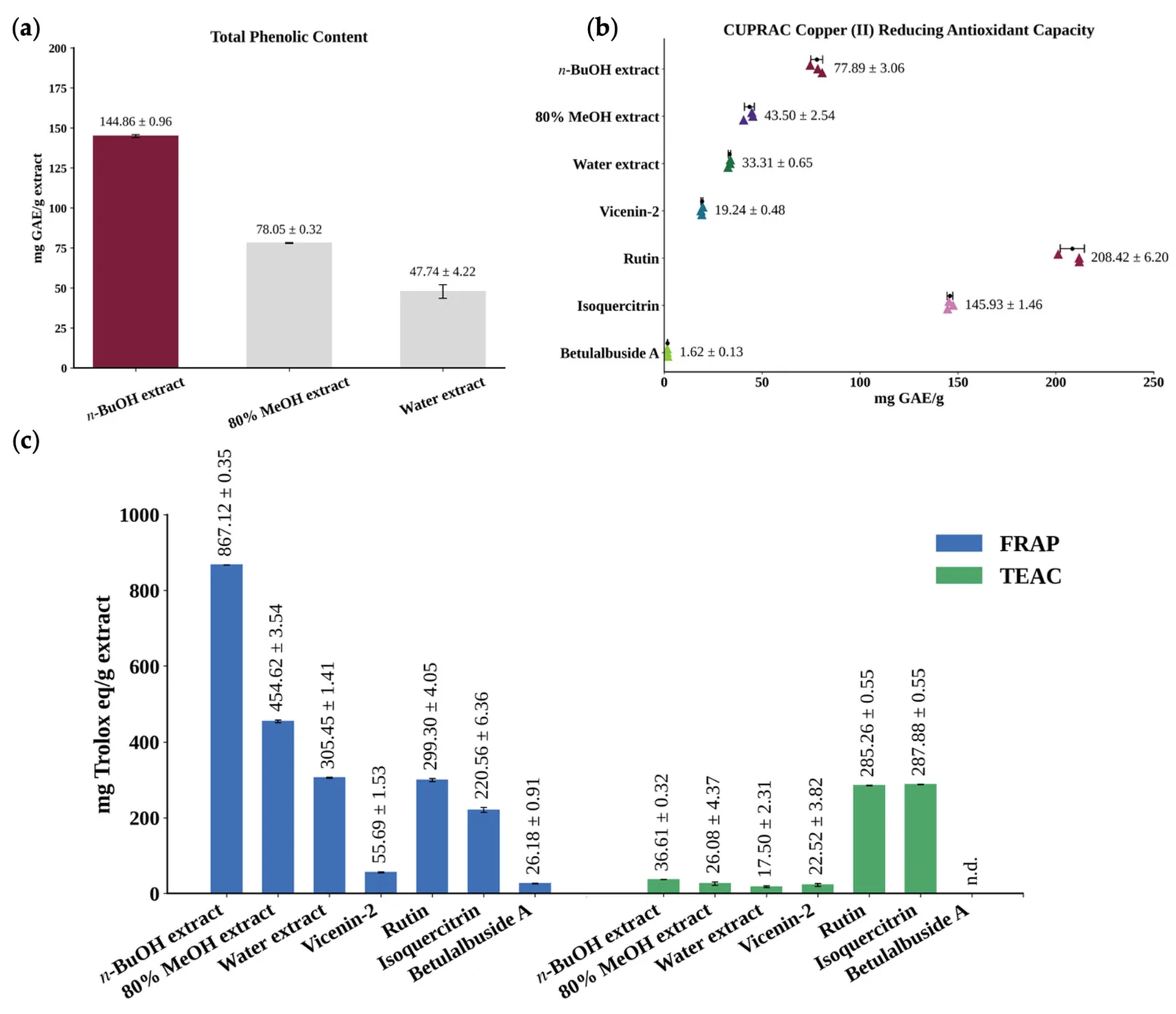
Total phenolic content (**a**), CUPRAC (**b**), FRAP and TEAC (**c**) antioxidant capacity of *E. sibthorpiana* extracts and some isolated compounds. Data are expressed as mean ± SD of three independent experiments, each performed in triplicate (*n* = 3).

According to the total phenolic content analysis, the *E. sibthorpiana*-*n*-BuOH extract exhibited the highest phenolic content among all extracts, with a value of 144.86 ± 0.96 mg gallic acid equivalents per gram of dry extract (GAE g^−1^ extract) (Figure 4a).

#### 3.3.2. Antioxidative Activity

In the cupric ion reducing antioxidant capacity (CUPRAC) assay, the *E. sibthorpiana*-*n*-BuOH extract exhibited the highest reducing capacity (77.89 ± 3.06 mg GAE g^−1^ extract), whereas among the isolated compounds, compound **2** showed the greatest activity (208.40 ± 6.20 mg GAE g^−1^ extract). Likewise, in the ferric reducing antioxidant power (FRAP) assay, the *E. sibthorpiana*-*n*-BuOH extract again displayed the most pronounced ferric-reducing capacity (867.12 ± 0.35 mg TE g^−1^ extract), with compound **2** once more showing the highest activity among the isolated compounds (299.30 ± 4.05 mg TE g^−1^ extract). In the Trolox equivalent antioxidant capacity (TEAC) assay, the *E. sibthorpiana*-*n*-BuOH extract exhibited the highest activity among the tested extracts (36.61 ± 0.32 mg TE g^−1^ extract), while compound **3** showed the highest TEAC value among the isolated compounds (287.88 ± 0.55 mg TE g^−1^ extract). Overall, the *E. sibthorpiana*-*n*-BuOH extract consistently exhibited the strongest antioxidant activity across all three assays. Among the isolated compounds, rutin (compound **2**) showed the highest activity in the CUPRAC and FRAP assays, whereas isoquercitrin (compound **3**) was the most active in the TEAC assay (Figure 4b,c).

**Figure 5:**
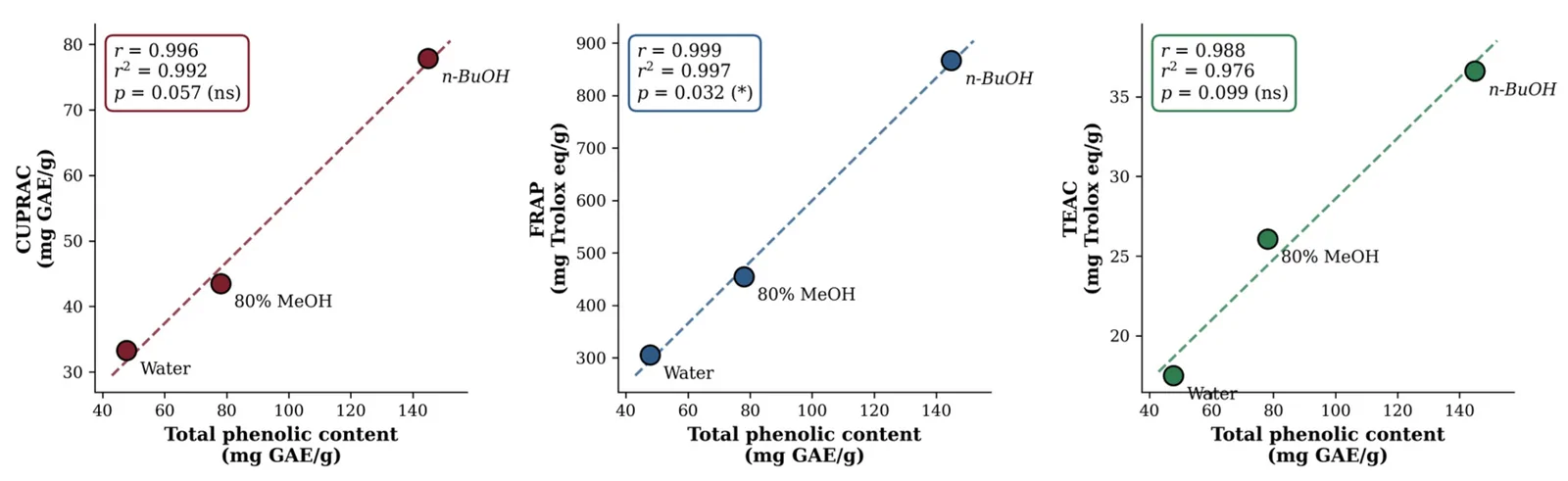
Pearson correlation between the total phenolic content and the CUPRAC, FRAP and TEAC antioxidant capacity of the *E. sibthorpiana* extracts (*n*-BuOH, 80% MeOH and water; *n* = 3). Solid lines represent the linear regression fit. r, Pearson correlation coefficient; R^2^, coefficient of determination; *p* < 0.05 (*); ns, not significant.

Since total phenolic content was determined only for the crude methanol extract and its *n*-butanol and aqueous subfractions (and not for the isolated compounds, which are individual pure substances), the correlation between total phenolic content and antioxidant capacity was evaluated across these three extracts (*n* = 3). Total phenolic content showed a very strong positive correlation with all three antioxidant assays (CUPRAC: r = 0.996, R^2^ = 0.992; FRAP: r = 0.999, R^2^ = 0.997, *p* < 0.05; TEAC: r = 0.988, R^2^ = 0.976). Owing to the limited number of extracts (*n* = 3), statistical significance was reached only for the FRAP assay; nevertheless, the uniformly high correlation coefficients (all r > 0.98) suggest a strong association between phenolic content and the antioxidant capacity of the extracts (Figure 5).

#### 3.3.3. Protective Effects of *E. sibthorpiana* MeOH Extract Against DNA Damage

**Figure 6:**
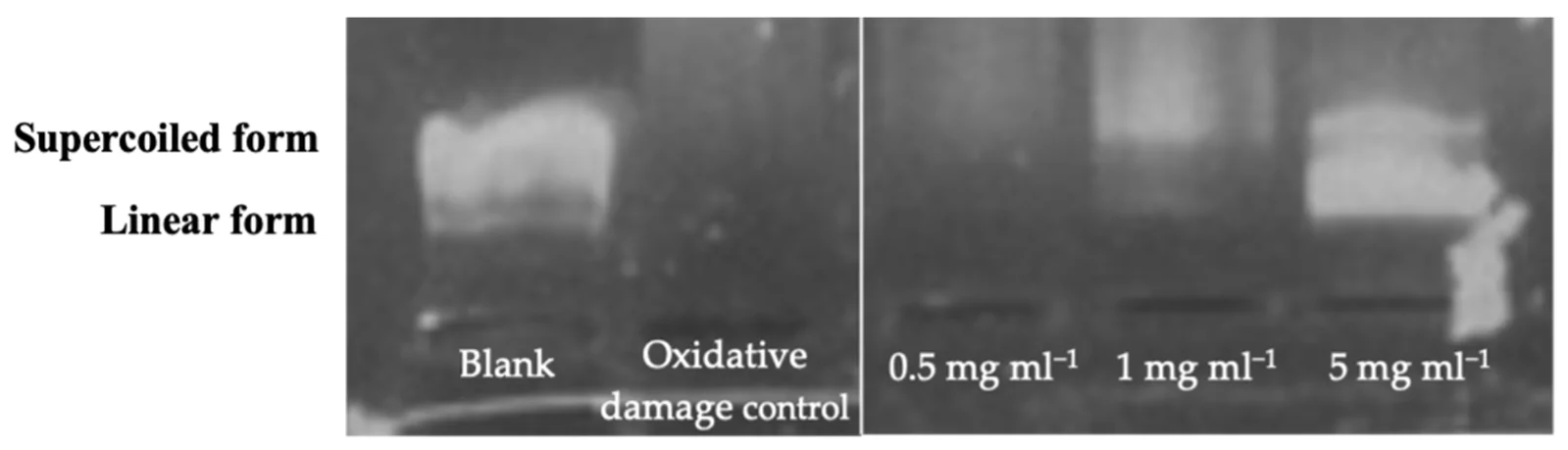
DNA agarose gel electrophoresis showing the concentration-dependent DNA-protective effect of the *E. sibthorpiana* MeOH extract (0.5, 1, and 5 mg mL^−1^) against H_2_O_2_/UV-induced oxidative damage, providing qualitative visual evidence (Supplementary File S3, Figure S44).

The plasmid DNA protection assay revealed that *E. sibthorpiana* MeOH extracts exhibited significant protection against H_2_O_2_/UV-induced oxidative damage at 5 mg mL^−1^ (53.83 ± 2.49%; *p* < 0.05) and retained notable protective activity (39.75 ± 2.49%) at 1 mg mL^−1^. The percentage of the relative supercoiled DNA form (%RV) increased in a concentration-dependent manner compared with the oxidative damage control (Figure 6). The extract preserved the native supercoiled structure of pBR322 plasmid DNA, indicating its potential antigenotoxic and antioxidant properties. The protection rates were in good agreement with the in vitro antioxidant capacity assays, suggesting a correlation between the redox activity and DNA-protective potential.

#### 3.3.4. Protective Effects of *E. sibthorpiana* Subfractions, and Isolated Compounds

**Figure 7:**
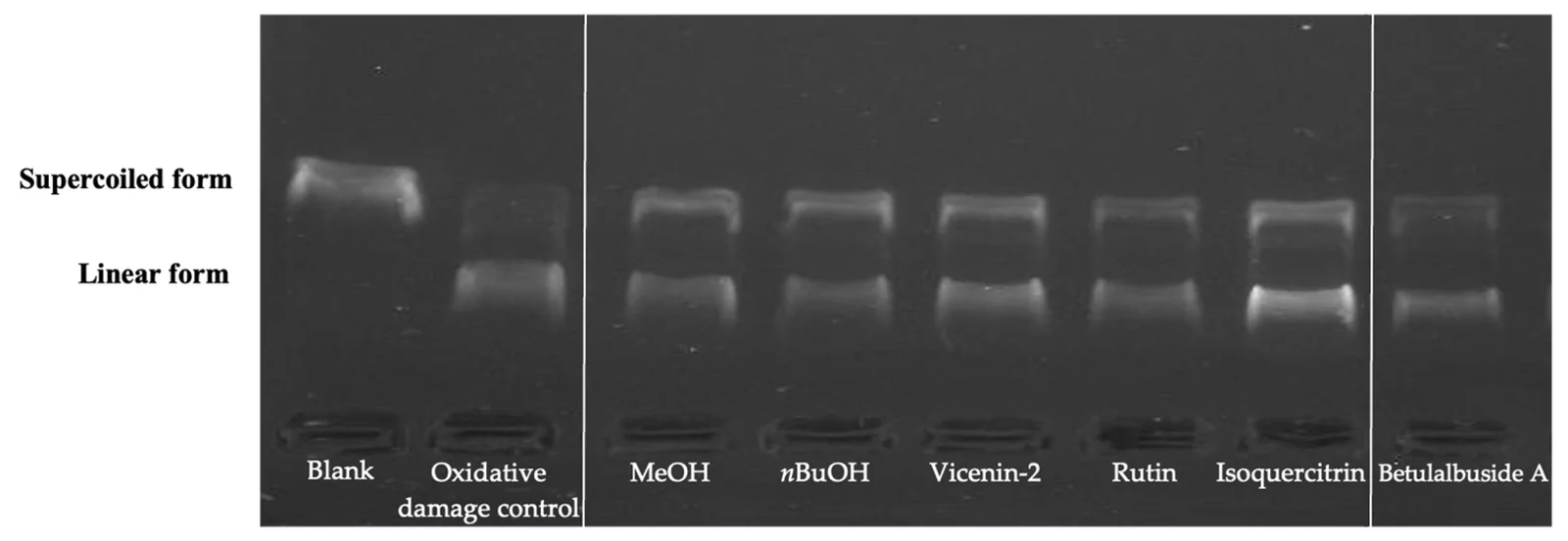
DNA agarose gel electrophoresis results of *E. sibthorpiana*-MeOH extract, *E. sibthorpiana*-*n*-BuOH extract, and four pure compounds obtained from these extracts (Supplementary File S3, Figure S45).

Further evaluation of the *E. sibthorpiana*-MeOH and *E. sibthorpiana*-*n*-BuOH extracts, and their isolated compounds demonstrated that both crude extracts provided significant DNA-protective effects compared with the oxidative damage control (*p* < 0.05). Among the isolated compounds, compounds **2** and **4** showed the highest preservation of the supercoiled plasmid DNA form (%RV ≈ 47–48%) (Table 2; Figure 7).

### 3.4. In Vivo Biological Activities

The genotoxic and antigenotoxic effects of *E. sibthorpiana* extracts and isolated compounds were evaluated using the *D. melanogaster* wing SMART assay. The negative control group showed low mutant spot frequencies, consistent with the expected spontaneous background level in *Drosophila* somatic wing tissue. In contrast, EMS treatment increased mutant spot frequency across all scored categories, confirming the sensitivity of the assay and the validity of the positive control. In the genotoxicity assay, the aqueous, butanolic, methanolic, and *n*-hexane extracts did not produce a statistically significant elevation in total spot frequency relative to the negative control (Table 3). A comparable pattern was observed for the tested compounds: none of the tested isolated compounds **1**–**4**, produced a statistically significant increase in total mutant spot frequency compared with the negative control under the experimental conditions described in Section 2.8. (Table 4). According to the SMART classification criteria, these responses were interpreted as negative or inconclusive rather than clearly genotoxic. Although isolated inconclusive or weak-positive diagnoses were observed for some individual endpoints, these responses were not consistently observed across all endpoints and doses, and therefore were not interpreted as evidence of biologically relevant genotoxicity. These findings indicate that the tested extracts and compounds did not display detectable intrinsic mutagenic or recombinogenic activity in *D. melanogaster* somatic wing cells under the tested concentrations and exposure conditions used.

In the antigenotoxicity assay, co-treatment with EMS and *E. sibthorpiana* extracts reduced EMS-induced mutant spot frequencies in a dose-dependent manner for most samples (Table 3). The *n*-BuOH and MeOH extracts showed the most pronounced protective effects, with the 0.15 mg mL^−1^ dose reducing total spot frequency by 74.54% and 82.72%, respectively, relative to the EMS-only group. The aqueous and *n*-hexane extracts also significantly reduced EMS-induced damage, although to a lesser extent, with maximal inhibition values of 68.18% and 60.90%, respectively. Overall, the magnitude of antigenotoxic protection followed the order MeOH > *n*-BuOH > Water > *n*-Hexane extract, broadly paralleling the total phenolic content and the in vitro antioxidant capacity determined for these extracts.

Among the isolated compounds, all four compounds reduced EMS-induced total mutant spot frequency, with inhibition values ranging from 47.11% to 74.03% (Table 4). Compound **2** (rutin) showed the strongest antigenotoxic effect, reaching 74.03% inhibition at 0.05 mg mL^−1^, the highest value recorded among the pure compounds and comparable to that of the *n*-BuOH extract. Compounds **1** (vicenin-2) showed moderate protective effects (61.53–63.46% inhibition), while compound **3** (isoquercitrin) showed slightly lower protection (56.73–58.65%). Compound **4** (betulalbuside A) displayed the weakest and most dose-dependent antigenotoxic activity, with inhibition decreasing from 56.73% at 0.05 mg mL^−1^ to 47.11% at 0.025 mg mL^−1^, the lowest value among all tested samples. Collectively, none of the tested *E. sibthorpiana* extracts or isolated compounds exhibited genotoxic or recombinogenic activity in the *D. melanogaster* wing SMART assay, whereas all exhibited antigenotoxic activity against EMS-induced DNA damage, with inhibition rates ranging from approximately 47% to 83% across extracts and compounds (Table 3 and Table 4).

## 4. Discussion

Natural products continue to represent a valuable source of structurally diverse secondary metabolites with relevance to drug discovery and biosafety assessment, and species belonging to the family Apiaceae are recognised for their characteristic accumulation of phenolic, terpenoid, and polyacetylenic constituents. Within this family, the genus *Echinophora* remains comparatively underexplored from a phytochemical standpoint, with most available data derived from essential oil analyses, while its non-volatile metabolite composition has received considerably less attention. The present study aimed to characterise the non-volatile secondary metabolites of *E. sibthorpiana* as a basis for subsequent antioxidant, DNA-protective, and genotoxicity/antigenotoxicity evaluations.

The structures of compounds **1**–**4** were established by detailed interpretation of 1D- and 2D-NMR spectra (^1^H, ^13^C, DEPT, HMQC, HMBC, NOESY, ROESY) supported by HR-ESI-MS data and confirmed through comparison with published spectroscopic data. Compound **1** gave HR-ESI-MS ion peaks at *m*/*z* 593.1526 [M − H]^−^ and *m*/*z* 595.1670 [M + H]^+^, consistent with the molecular formula C_27_H_30_O_15_ (calcd. 593.1511 and 595.1657, respectively). Its ^1^H-NMR spectrum displayed two anomeric proton signals together with the characteristic aromatic resonance pattern of an apigenin nucleus, indicative of a di-C-glycosylated flavone. Assignments obtained from HMQC, together with the key HMBC correlations from the anomeric protons to C-6 and C-8 of the aglycone, unequivocally established the presence of two β-glucopyranosyl units attached at these positions. The spectroscopic data were in excellent agreement with those reported for compound **1** as vicenin-2 (apigenin-6,8-di-C-glucoside) [57,62].

Compound **2** exhibited HR-ESI-MS ion peaks at *m*/*z* 611.1621 [M + H]^+^ and *m*/*z* 609.1474 [M − H]^−^, corresponding to the molecular formula C_27_H_30_O_16_ (calcd. 611.1606 and 609.1460, respectively). The NMR spectra revealed the characteristic quercetin-type flavonol skeleton together with two anomeric proton signals and a methyl doublet characteristic of a rhamnose unit, indicating a rutinose moiety attached at C-3. The sequential glycosidic linkages were confirmed by HMBC analysis, and the complete spectroscopic dataset matched that reported for rutin (quercetin-3-*O*-rutinoside) [59].

For compound **3**, HR-ESI-MS showed a prominent ion peak at *m*/*z* 463.0892 [M − H]^−^, consistent with the molecular formula C_21_H_20_O_12_ (calcd. 463.0881); together with the corresponding sodium adduct at *m*/*z* 487.0862 [M + Na]^+^. Compared with compound **2**, the NMR spectra showed an almost identical aglycone but only a single anomeric proton and the corresponding glucose carbon signals, indicating the presence of a single glucose residue. The HMBC correlation between the H-1″ and C-3 confirmed mono-*O*-glycosylation at C-3, and comparison with published data identified the compound as isoquercitrin (quercetin-3-*O*-β-glucopyranoside) [58].

The HR-ESI-MS spectra of compounds **4** displayed ion peaks at *m*/*z* 355.1747/355.1756 [M + Na]^+^ (positive mode) and *m*/*z* 331.1776/331.1772 [M − H]^−^ (negative mode), consistent with the molecular formula C_16_H_28_O_7_ (calcd. for 355.1728 and 331.1763, respectively). The ^1^H- and ^13^C-NMR spectra displayed the signal sets characteristic of an acyclic monoterpene-type aglycone bearing a β-D-glucopyranosyl unit, including olefinic methine/methylene signals, two singlet methyl groups attached to an oxygenated quaternary carbon, and an anomeric proton/carbon pair. Comprehensive HMQC and HMBC analyses established the complete structure and confirmed the glycosidic linkage between the glucose unit and the aglycone. The chemical shift and coupling-constant patterns of compounds **4** were closely comparable to those reported for acyclic monoterpene glucoside isolated from *Viburnum orientale* [60] and *Origanum acutidens* [62], differing mainly in the configuration at the oxygenated stereocenter of the monoterpenoid moiety. On this basis, compounds **4** were identified as betulalbuside A.

While vicenin-2, rutin, and isoquercitrin are reported here for the first time as isolated compounds from *E. sibthorpiana*, betulalbuside A represent the first isolation of acyclic monoterpene glucoside from the genus *Echinophora*. This finding extends the known chemical diversity of the genus beyond the flavonoid and terpenoid classes previously documented.

The LC-qTOF-MS-based metabolite distribution analysis across the six *Echinophora* species native to Türkiye provides additional context for interpreting the phytochemical profile of *E. sibthorpiana*. The consistent presence of rutin and isoquercitrin in all six species, together with the three benzyl-glycoside-type compounds (amburoside A, 3′-demethoxy-amburoside B, and 4′-hydroxy-salviifoside A), indicates that these constituents are not unique to *E. sibthorpiana* but rather represent conserved phytochemical features of the genus. Accordingly, they are likely to have limited diagnostic value for species-level differentiation. In contrast, the comparatively restricted distribution of betulalbuside A and echinophoroside C, detected only in *E. sibthorpiana* and a small subset of closely related taxa, suggests the existence of a more taxon-specific biosynthetic capacity within this group of species. This distribution pattern supports the hypothesis that these monoterpene- and phenolic-glycoside-type compounds may serve as chemotaxonomic markers for distinguishing *E. sibthorpiana* and its closest relatives from the remaining *Echinophora* taxa examined. From a biological perspective, the co-occurrence of widely distributed flavonoid glycosides (rutin, isoquercitrin, vicenin-2) with the more restricted betulalbuside-type glucoside in *E. sibthorpiana* suggests that the genotoxic and antigenotoxic properties evaluated in the present study may reflect contributions from both broadly conserved and species-specific metabolite classes. The possibility warrants further comparative investigation across the genus.

The pronounced antioxidant activity of the *E. sibthorpiana*-*n*-BuOH extract, and of the flavonoid glycosides rutin (**2**) and isoquercitrin (**3**) is well supported by previous reports highlighting the central role of the catechol-type B-ring (3′,4′-dihydroxyl) in determining the reducing and radical-scavenging capacities of flavonoids. For instance, flavone C-glycosides isolated from *Linum usitatissimum* showed that luteolin-derived compounds exhibited higher antioxidant activity than apigenin-derived compounds in CUPRAC and FRAP assays, an effect attributed to the ortho-dihydroxyl substitution at C-3′ and C-4′ of the B-ring [64]. Similarly, in the original CUPRAC method validation study, rutin was among the polyphenolic compounds exhibiting the highest cupric ion-reducing capacities [65], supporting the high CUPRAC value obtained for compound **2** in the present study. The slightly higher TEAC value of isoquercitrin compared with rutin observed in the present study is also in agreement with earlier findings. In a comparative evaluation of quercetin-3-*O*-glycosides, quercetin and its glycosides including isoquercitrin and rutin were shown to possess radical-scavenging and ferric-reducing capacities, with the aglycone quercetin showing the highest activity, indicating that the attached sugar moiety reduces the overall antioxidant potency relative to the parent aglycone [66]. Moreover, a mechanistic comparison of quercetin-3-*O*-glycosides demonstrated that isoquercitrin exhibited greater Fe^2+^-binding and ferric-reducing capacity than quercitrin, an effect linked to the free 6″-OH group on its glucosyl moiety [67], providing a structural basis for the comparable or slightly higher TEAC and FRAP performance of isoquercitrin relative to rutin in the present study.

The comparatively lower antioxidant activity of compound **1** (vicenin-2), a flavone C-diglycoside, relative to the *O*-glycosides rutin and isoquercitrin is likewise consistent with the previous structure-activity studies. In the same investigation, the antioxidant activity of apigenin-type flavone C-glycosides followed the order apigenin > vitexin > isovitexin > vicenin-2 > vicenin-1 [64], indicating that both glycosylation and the absence of an ortho-dihydroxylated B-ring substantially limit the reducing capacity of vicenin-2 compared with more hydroxylated flavonoids such as rutin and isoquercitrin. Finally, the negligible CUPRAC and FRAP activities and the absence of detectable TEAC activity for compounds **4** (betulalbuside A) is in line with the well-established relationship between phenolic content and reducing/radical-scavenging capacity. In a comparative evaluation of 30 plant extracts, FRAP activity was found to be strongly correlated with total phenolic content [68], indicating that compounds lacking phenolic hydroxyl groups contribute little to reducing or radical-scavenging activity. Similarly, the total phenolic content of the *E. sibthorpiana* extracts correlated strongly and positively with all three antioxidant assays in the present study (CUPRAC, FRAP and TEAC; all r > 0.98; Figure 5), and the ranking of phenolic content paralleled that of antioxidant activity (*n*-BuOH > 80% MeOH > water), further supporting phenolic constituents as the main contributors to the antioxidant capacity of the extracts. Since betulalbuside A is monoterpene glycoside devoid of phenolic substituents, their limited contribution to the overall antioxidant capacity of the *E. sibthorpiana*-*n*-BuOH extract is structurally expected.

The DNA protection results show a notable pattern: both crude extracts (*E. sibthorpiana*-MeOH, 56.87 ± 2.23%; *E. sibthorpiana*-*n*-BuOH, 60.78 ± 2.12%) preserved the supercoiled plasmid DNA form to a greater extent than any of the individual isolated compounds (45.59–48.07%), despite the compounds being derived from these same extracts. This finding suggests that the overall DNA-protective activity of the extracts may not be attributable to a single dominant constituent, but rather to the combined or synergistic action of multiple phenolic and non-phenolic components present in the crude extracts, an outcome consistent with previous observations that mixtures of plant phenolics can produce genoprotective effects exceeding the sum of their individual contributions [66]. Among the isolated compounds, compound **2** (rutin, 48.07 ± 1.19%) and compound **4** (betulalbuside A, 47.46 ± 2.02%) showed the highest DNA-protective activity, followed closely by compounds **1** (vicenin-2, 45.78 ± 1.84%), **3** (isoquercitrin, 45.59 ± 3.12%), all of which were statistically similar. Of particular interest, this ranking does not fully parallel the antioxidant capacity results (CUPRAC, FRAP, TEAC), where rutin and isoquercitrin showed markedly higher activity than vicenin-2 and the betulalbuside A. The relatively uniform DNA-protective activity across structurally diverse compounds, including the monoterpene glycoside betulalbuside A, which displayed negligible or undetectable antioxidant activity in the TEAC assay, suggests that DNA protection in the present assay may be mediated, at least in part, by mechanisms other than direct radical scavenging or metal-ion reducing capacity. Additional mechanisms, including direct interaction with the DNA or chelation of metal ions involved in hydroxyl radical generation, are also likely to contribute.

The wing SMART results demonstrate that neither the crude extracts nor compounds **1**–**4** produced a significant increase in total mutant spot frequency relative to the negative control, indicating that, under the tested concentrations, *E. sibthorpiana* and its isolated constituents do not possess detectable intrinsic genotoxic or recombinogenic activity in *D. melanogaster* somatic wing cells. This is a favourable outcome from a biosafety standpoint, since it suggests that the antioxidant and DNA-protective properties of these constituents are not accompanied by an inherent mutagenic liability, an important consideration when evaluating natural products as candidates for nutraceutical or pharmaceutical use. In contrast, all extracts and all four isolated compounds significantly reduced EMS-induced mutant spot frequency, confirming a consistent antigenotoxic potential across the whole sample set. Among the extracts, the order of protection (MeOH > *n*-BuOH > Water > *n*-Hexane) broadly paralleled the total phenolic content and in vitro antioxidant capacity determined for these fractions, supporting a contribution of phenolic antioxidants to the in vivo protective effect. Among the isolated compounds, rutin (**2**) showed the strongest antigenotoxic activity (up to 74.03% inhibition), in line with previous reports demonstrating that rutin and related flavonol glycosides can significantly reduce DNA damage induced by chemical mutagens, an effect attributed to a combination of radical scavenging and interference with the reactivity or metabolic activation of the genotoxic agent [69,70]. Vicenin-2 (**1**) displayed moderate protective effects, isoquercitrin (**3**) showed slightly lower activity, and betulalbuside A (**4**) was the least effective and most dose-dependent of the tested compounds. Betulalbuside A gave a positive result for total spots only at higher concentration (0.05 mg mL^−1^). Because this result appeared for only one type of spot, was not seen again at 0.025 mg mL^−1^, was not supported by the total *mwh* clone count, and did not increase with dose, it is not considered a genuine sign of genotoxicity, following the standard evaluation method of Frei and Würgler [51].

For several samples, the antigenotoxic effect did not increase steadily with dose. For the crude MeOH, *n*-BuOH and aqueous extracts, and for vicenin-2, the lower of the two tested concentrations gave an inhibition of EMS-induced total spot frequency equal to or slightly higher than that of the higher concentration (for example, 82.72% versus 80.90% for the MeOH extract, and 68.18% versus 60.00% for the aqueous extract), whereas rutin, isoquercitrin and betulalbuside A showed the expected dose-dependent increase in protection. Two explanations can be offered. First, where the differences were small (about 3% or less), the two concentrations gave essentially the same protection, within the normal variation in the wing-spot assay. Second, and mainly for the larger differences seen with the crude extracts, antioxidant plant compounds are known to protect most effectively at low-to-moderate concentrations, with the effect levelling off or decreasing at higher concentrations. This is because polyphenols such as quercetin and its glycosides can begin to act as pro-oxidants at higher concentrations (that is, they start to generate rather than neutralise reactive oxygen species), which partly cancels their protective effect and, at very high doses, may even cause slight DNA damage [69,71,72]. That this reversal was clearer for the multi-component crude extracts than for the single isolated compounds fits the combined action of their different constituents. The slightly higher protection at the lower dose is therefore not an experimental error but an expected feature of complex antioxidant mixtures, and it supports the use of low, non-toxic concentrations for this kind of evaluation.

The concentrations used in the in vivo SMART assay (0.025–0.30 mg mL^−1^ in the larval food) were lower than those needed in the cell-free plasmid DNA-protection assay (0.5–5 mg mL^−1^) and were kept within non-lethal limits for the developing larvae. Because the wing SMART is a whole-animal test, the antigenotoxic effects seen here were obtained only after the test substances had been eaten, absorbed and metabolized by the larvae, so they already reflect how available these compounds are inside the animal. The concentrations we used are also similar to those in earlier SMART studies of related flavonoids; for example, vitexin, an apigenin-6,8-di-C-glycoside closely related to vicenin-2, was tested in the same assay at 0.15–0.6 mM (about 0.065–0.26 mg mL^−1^) [53]. Even so, these concentrations should be transferred to mammals with caution, because flavonoid glycosides such as rutin and isoquercitrin are heavily metabolized and have low and variable oral bioavailability [54]. Accordingly, pharmacokinetic and mechanistic studies will be necessary to establish the translational relevance of these findings. Overall, the present results demonstrate that the favourable DNA-protective profile of *E. sibthorpiana* extends to an in vivo eukaryotic model without evidence of intrinsic genotoxicity. Together with the strong antioxidant and antigenotoxic activities observed, these findings highlight the potential of *E. sibthorpiana* and its major glycoside constituents as safe, multi-mechanistic chemopreventive agents worthy of further pharmacological investigation.

## 5. Conclusions

Phytochemical investigation of *Echinophora sibthorpiana* Guss. resulted in the isolation and structural characterization of four secondary metabolites, namely vicenin-2, rutin, isoquercitrin, and betulalbuside A, all of which are isolated here for the first time from this species. Moreover, betulalbuside A represents the first report an acyclic monoterpene glucoside from the genus *Echinophora*. LC-qTOF-MS profiling of six *Echinophora* species in Türkiye identified rutin, isoquercitrin, and three benzyl-glycoside-type compounds as genus-wide conserved constituents, while betulalbuside A and echinophoroside C showed a restricted distribution, suggesting their potential value as chemotaxonomic markers. Among the tested samples, the *n*-BuOH fraction and the flavonol glycosides rutin and isoquercitrin exhibited the strongest antioxidant and DNA-protective activities, consistent with their phenolic substitution patterns. In contrast, the crude extracts outperformed individual compounds in the plasmid DNA-protection assay, indicating synergistic interactions among co-occurring metabolites. In the in vivo *D. melanogaster* wing SMART assay, none of the samples displayed genotoxic activity, whereas all samples exhibited antigenotoxic activity to varying degrees against EMS-induced DNA damage, with the *n*-BuOH and MeOH extracts showing the highest inhibition (71.81–82.72%)**.** Collectively, these findings demonstrate both the genetic safety and the chemopreventive potential of *E. sibthorpiana* and support further investigation of *Echinophora* species and their glycoside constituents as safe antioxidant and antigenotoxic agents with potential nutraceutical and pharmacological applications. In the future, it will be necessary to develop new carrier drug formulations that will increase the in vivo efficacy of active molecules and to conduct detailed mechanism studies.

## Figures and Tables

**Table 1:** Collection data and herbarium numbers for the *Echinophora* species studied.

The Plant Name	Place of Collection	Altitudes	Herbarium No.
*E. tournefortii* Jaub. & Spach	Ankara: Beytepe, Hacettepe University Campus	20 m	HUEF22003
*E. sibthorpiana* Guss.	Ankara: Beytepe, Hacettepe University Campus, Ankara	900 m	HUEF22002
*E. orientalis* Hedge & Lamond	Van: Erciş, Y. Işıklı and Çakırbey village surroundings, road	900 m	HUEF22004
*E. trichophylla* J.E. Smith	Bilecik: Başköy, slopes	85 m	HUEF22005
*E. chrysantha* Freyn & Sint.	Erzincan: Spikor Mountain, western exit, Işıkpınar Village, roadside	900 m	HUEF22007
*E. lamondiana* Yıldız & Z. Bahcecioglu	Malatya: Between Hekimhan and Sivas, around Boğazgören, mining road, roadside	68 m	HUEF22008

**Table 2:** Level of supercoiled DNA damage protection by *E. sibthorpiana* extracts and isolated compounds (gel shown in Figure 7).

Samples	% RV (Relative Value): Mean ± SD
Blank	100
Oxidative Damage Control	28.20 ± 1.12
Extracts	
*E. sibthorpiana*-MeOH	56.87 ± 2.23 *
*E. sibthorpiana*-*n*-BuOH	60.78 ± 2.12 *
Isolated compounds	
**1** (Vicenin-2)	45.78 ± 1.84 *
**2** (Rutin)	48.07 ± 1.19 *
**3** (Isoquercitrin)	45.59 ± 3.12 *
**4** (Betulalbuside A)	47.46 ± 2.02 *

* *p* < 0.05 vs. oxidative damage control (the isolated compounds obtained in sufficient quantities were studied).

**Table 3:** Results from the *Drosophila* wing spot test evaluating the genotoxic and antigenotoxic potential of extracts from the *E. sibthorpiana.* In the antigenotoxicity assays, all doses of all extracts were simultaneously treated to *Drosophila* larvae with 1 mM EMS.

Treatment		Number of Wings	Frequency of Spots per Wing and Diagnosis						
			SSS (m = 2)	LSS (m = 2)	TS (m = 5)	TMS (m = 2)	Total Spots (m = 2)	CIF	Inhibition (%)
Control (Water)	Doses	40	12 (0.30)	1 (0.02)	0 (0.00)	13 (0.32)	13 (0.32)	1.33	
EMS (1 mM)		80	76 (0.95) +	32 (0.40) +	2 (0.02) +	98 (1.22) +	110 (1.37) +	5.02	
*E. sibthorpiana*-MeOH	0.12 mg mL^−1^	40	10 (0.25) −	3 (0.07) i	0 (0.00) i	13 (0.32) i	13 (0.32) i	1.33	
	0.06 mg mL^−1^	40	9 (0.23) −	2 (0.05) i	0 (0.00) i	11 (0.28) −	11 (0.28) −	1.13	
*E. sibthorpiana*-MeOH + EMS	0.30 mg mL^−1^	60	15 (0.25) −	6 (0.10) −	0 (0.00)	15 (0.25) −	21 (0.35) −	1.02	80.90
	0.15 mg mL^−1^	60	17 (0.28) −	2 (0.03) −	0 (0.00)	17 (0.28) −	19 (0.31) −	1.16	82.72
*E. sibthorpiana-n*-BuOH	0.10 mg mL^−1^	40	9 (0.22) −	2 (0.05) i	0 (0.00) i	11 (0.28) −	11 (0.28) −	1.13	
	0.05 mg mL^−1^	40	7 (0.18) −	1 (0.02) i	1 (0.02) i	8 (0.20) −	9 (0.22) −	0.82	
*E. sibthorpiana-n*-BuOH + EMS	0.30 mg mL^−1^	70	24 (0.34) i	6 (0.08) −	1 (0.01) i	28 (0.04) −	31 (0.44) −	1.63	71.81
	0.15 mg mL^−1^	60	19 (0.31) −	8 (0.13) −	1 (0.01) i	22 (0.37) −	28 (0.46) −	1.50	74.54
*E. sibthorpiana*-*n*-hexane	0.15 mg mL^−1^	40	4 (0.10) −	0 (0.00) i	0 (0.00) i	4 (0.10) −	4 (0.10) −	0.41	
	0.075 mg mL^−1^	40	4 (0.10) −	2 (0.05) i	1 (0.02) i	7 (0.18) −	7 (0.18) −	0.72	
*E. sibthorpiana*-*n*-hexane + EMS	0.30 mg mL^−1^	70	32 (0.45) i	11 (0.15) −	0 (0.01)	42 (0.60) −	43 (0.61) −	2.45	60.90
	0.15 mg mL^−1^	70	28 (0.40) i	6 (0.18) −	0 (0.00)	34 (0.48) −	34 (0.48) −	1.99	60.09
*E. sibthorpiana*-Water	0.10 mg mL^−1^	40	8 (0.20) −	0 (0.00) i	1 (0.02) i	9 (0.22) −	9 (0.22) −	0.92	
	0.05 mg mL^−1^	40	6 (0.15) −	3 (0.07) i	1 (0.02) i	8 (0.20) −	10 (0.25) −	0.82	
*E. sibthorpiana*-Water + EMS	0.30 mg mL^−1^	54	28 (0.51) i	16 (0.29) i	0 (0.00)	34 (0.62) i	44 (0.81) −	2.58	60.00
	0.15 mg mL^−1^	50	22 (0.44) i	12 (0.24) −	1 (0.02) i	29 (0.58) −	35 (0.70) −	2.37	68.18

The frequencies are given in parenthesis. Symbols next to values signify the following: +, positive mutagenic effect; −, no mutagenic effect; i, inconclusive effect (*p* = 0.05); Statistical diagnosis according to Frei and Würgler (1988) [51]. SSS: Small single spots, LSS: Large single spots, TS: Twin Spots, TMS: Total *mwh* spots, CIF: Clone Induction Frequency. Forty wings randomly selected from the emerged adults were scored per treatment (80 for the EMS positive control).

**Table 4:** Results from the *Drosophila* wing spot test evaluating the genotoxic and antigenotoxic potential of pure compounds from the *E. sibthorpiana.* In the antigenotoxicity assays, all doses of all substances were simultaneously treated to *Drosophila* larvae with 1 mM EMS.

Treatment		Number of Wings	Frequency of Spots per Wing and Diagnosis						
			SSS (m = 2)	LSS (m = 2)	TS (m = 5)	TMS (m = 2)	Total Spots (m = 2)	CIF	Inhibition (%)
Control (Water)	Doses	40	12 (0.30)	1 (0.02)	0 (0.00)	13 (0.32)	13 (0.32)	1.33	
EMS (1 mM)		80	62 (0.77) +	40 (0.50) +	2 (0.02) +	92 (1.15) +	104 (1.30) +	4.71	
**1** (Vicenin-2)	0.05 mg mL^−1^	40	13 (0.32) i	3 (0.07) i	0 (0.00)	15 (0.37) i	16 (0.40) i	1.53	
	0.025 mg mL^−1^	40	11 (0.27) −	3 (0.07) i	0 (0.00)	13 (0.32) i	14 (0.35) i	1.33	
**1** (Vicenin-2) + EMS	0.05 mg mL^−1^	40	24 (0.60) −	16 (0.40) −	0 (0.00)	38 (0.95) −	40 (1.00) −	3.89	61.53
	0.025 mg mL^−1^	40	22 (0.55) −	16 (0.40) −	0 (0.00)	38 (0.95) −	38 (0.95) −	3.89	63.46
**2** (Rutin)	0.05 mg mL^−1^	40	15 (0.37) −	4 (0.10) i	0 (0.00)	17 (0.42) i	19 (0.47) i	1.74	
	0.025 mg mL^−1^	40	15 (0.37) i	2 (0.05) i	0 (0.00)	15 (0.37) i	17 (0.42) i	1.53	
**2** (Rutin) + EMS	0.05 mg mL^−1^	40	14 (0.35) −	13 (0.32) −	0 (0.00)	25 (0.62) −	27 (0.67) −	2.56	74.03
	0.025 mg mL^−1^	40	24 (0.60) −	14 (0.35) −	0 (0.00)	38 (0.95) −	38 (0.95) −	3.89	63.46
**3** (Isoquercitrin)	0.05 mg mL^−1^	40	16 (0.40) −	4 (0.10) i	0 (0.00)	18 (0.45) i	20 (0.50) i	1.84	
	0.025 mg mL^−1^	40	11 (0.27) i	2 (0.05) i	0 (0.00)	13 (0.32) i	13 (0.32) i	1.33	
**3** (Isoquercitrin) + EMS	0.05 mg mL^−1^	40	28 (0.70) −	15 (0.37) −	0 (0.00)	37 (0.92) −	43 (1.07) −	3.79	58.65
	0.025 mg mL^−1^	40	26 (0.65) −	19 (0.47) z	0 (0.00)	37 (0.92) −	45 (1.12) −	3.79	56.73
**4** (Betulalbuside A)	0.05 mg mL^−1^	40	20 (0.50) i	3 (0.07) i	1 (0.00) i	22 (0.55) −	24 (0.60) +	2.25	
	0.025 mg mL^−1^	40	14 (0.35) i	4 (0.10) i	0 (0.00) i	16 (0.40) −	18 (0.45) i	1.63	
**4** (Betulalbuside A) + EMS	0.05 mg mL^−1^	40	31 (0.77) −	14 (0.35) −	0 (0.00)	42 (1.05) i	45 (1.12) −	4.30	56.73
	0.025 mg mL^−1^	40	32 (0.80) −	23 (0.57) −	0 (0.00)	51 (1.27) −	55 (1.37) −	5.22	47.11

The frequencies are given in parenthesis. Symbols next to values signify the following: +, positive mutagenic effect; −, no mutagenic effect; z, weak positive; i, inconclusive effect (*p* = 0.05); Statistical diagnosis according to Frei and Würgler (1988) [51]. SSS: Small single spots, LSS: Large single spots, TS: Twin Spots, TMS: Total *mwh* spots, CIF: Clone Induction Frequency. Forty wings randomly selected from the emerged adults were scored per treatment (80 for the EMS positive control).

## Data Availability

The experimental data supporting this work are accessible within the article and its Supplementary Files.

## Supplementary Materials

The following supporting information can be downloaded at https://www.mdpi.com/article/10.3390/antiox15081009/s1: Supplementary File S1, Figures S1–S32: 1D- and 2D-NMR (^1^H, ^13^C, DEPT, HSQC, HMBC, NOESY, ROESY) and HR-ESI-MS data of Compound **1**–**4**; Supplementary File S2, Figures S33–S43: LC/QTOF-MS-based extracted ion chromatogram (EIC) analysis of target compounds in methanolic extracts of six Turkish *Echinophora* species; Supplementary File S3, Figures S44 and S45: Original uncropped gel electrophoresis images of the plasmid DNA protection assay.

## Abbreviations

The following abbreviations are used in this manuscript:

ABTS	2,2-Azino-bis(3-ethylbenzothiazoline-6-sulfonic acid)
ANOVA	Analysis of variance
COSY	Correlation spectroscopy
CUPRAC	Cupric ion reducing antioxidant capacity
DEPT	Distortionless Enhancement by Polarization Transfer
DNA	Deoxyribonucleic acid
EMA	European Medicines Agency
EMS	Ethyl methanesulfonate
EIC	Extracted Ion Chromatogram
ESI	Electrospray ionization
FDA	U.S. Food and Drug Administration
flr^3^	Flare (*Drosophila* wing hair marker)
FRAP	Ferric reducing antioxidant power
GAE	Gallic acid equivalents
GC-MS	Gas chromatography-mass spectrometry
HMBC	Heteronuclear Multiple Bond Correlation
HR-ESI-MS	High-resolution electrospray ionization mass spectrometry
HSQC	Heteronuclear Single Quantum Coherence
HUEF	Herbarium of Hacettepe University, Faculty of Pharmacy
IOC	Densitometric intensity of open-circular DNA form
ISC	Densitometric intensity of supercoiled DNA form
LC/qTOF-MS	Liquid chromatography coupled with quadrupole time-of-flight mass spectrometry
MeOH	Methanol
mwh	Multiple wing hairs (*Drosophila* wing hair marker)
*n*-BuOH	*n*-Butanol
NMR	Nuclear magnetic resonance
NOESY	Nuclear Overhauser Effect Spectroscopy
PBS	Phosphate-buffered saline
ROESY	Rotating-frame Overhauser Effect Spectroscopy
Q-TOF	Quadrupole time-of-flight
%RS	Relative supercoiled DNA protection value
%RV	Relative supercoiled DNA content
%S	Percentage of supercoiled DNA form
SD	Standard deviation
SMART	Somatic Mutation and Recombination Test
TBE	Tris-borate-EDTA buffer
TE	Trolox equivalents
TEAC	Trolox equivalent antioxidant capacity
TMS	Tetramethylsilane
TPC	Total phenolic content
TPTZ	2,4,6-Tripyridyl-s-triazine
TRPA1	Transient receptor potential ankyrin type-1
UV	Ultraviolet
WHO	World Health Organization
