# Supplementary material for: Phytochemical Analysis and Bioevaluation of Echinophora sibthorpiana Guss. (Apiaceae): Antioxidant Capacity, DNA Protection and In Vivo Genotoxic Safety
Source: Antioxidants (Basel). 2026 Aug 13;15(8):1009. doi: 10.3390/antiox15081009 (PMC13509921; doi:10.3390/antiox15081009)
Supplement: Supplementary file 1 [file antioxidants-15-01009-s001.zip › Supplementary File S1.pdf]

Supplementary File S1: 1D- and 2D-NMR (^{1}H, ^{13}C, DEPT, HSQC, HMBC, NOESY, ROESY) and HR-ESI-MS data of Compounds 1-4

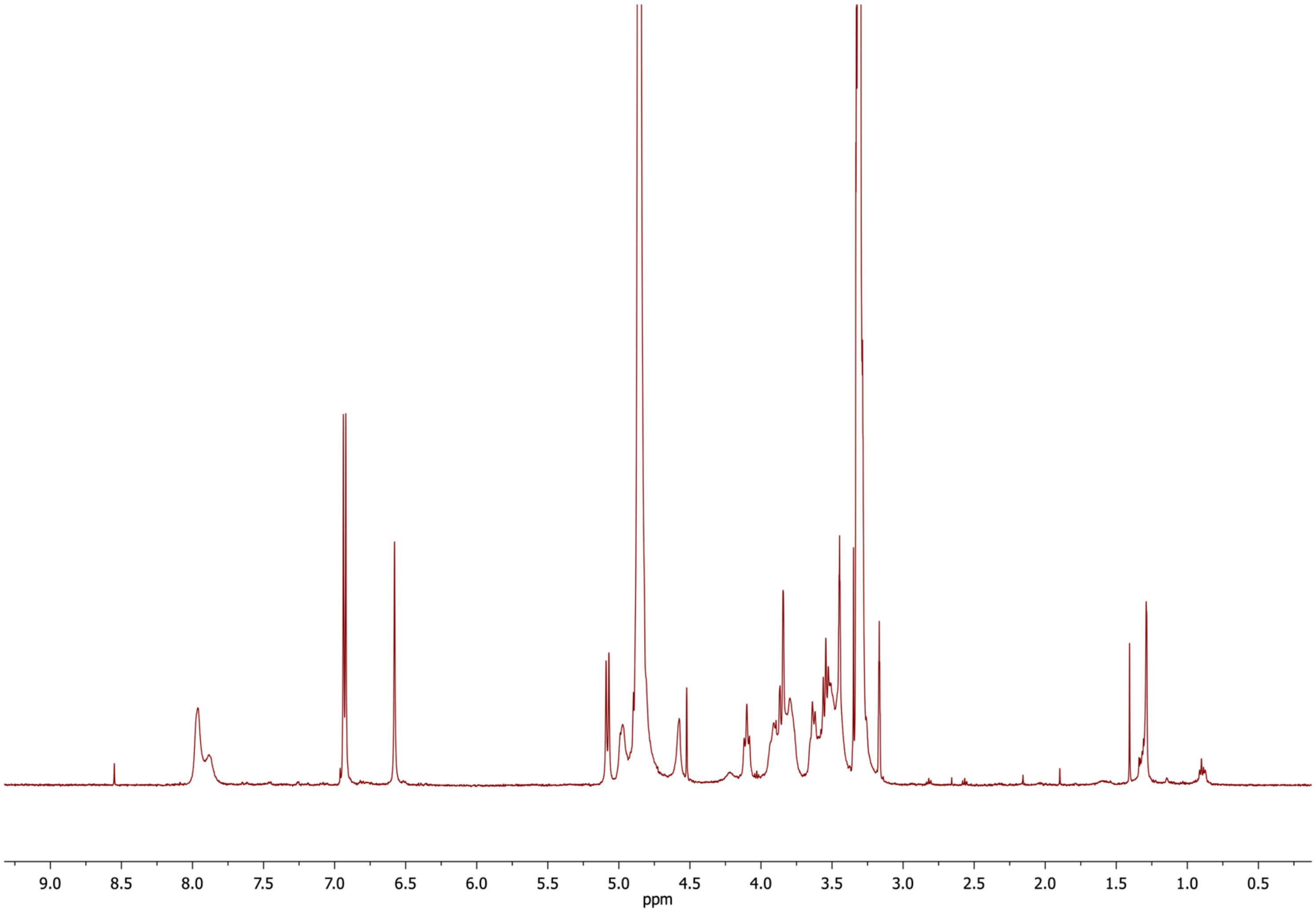

Figure S1. ^{1}H NMR spectrum of Compound **1** (CD_3OD, 500 MHz).

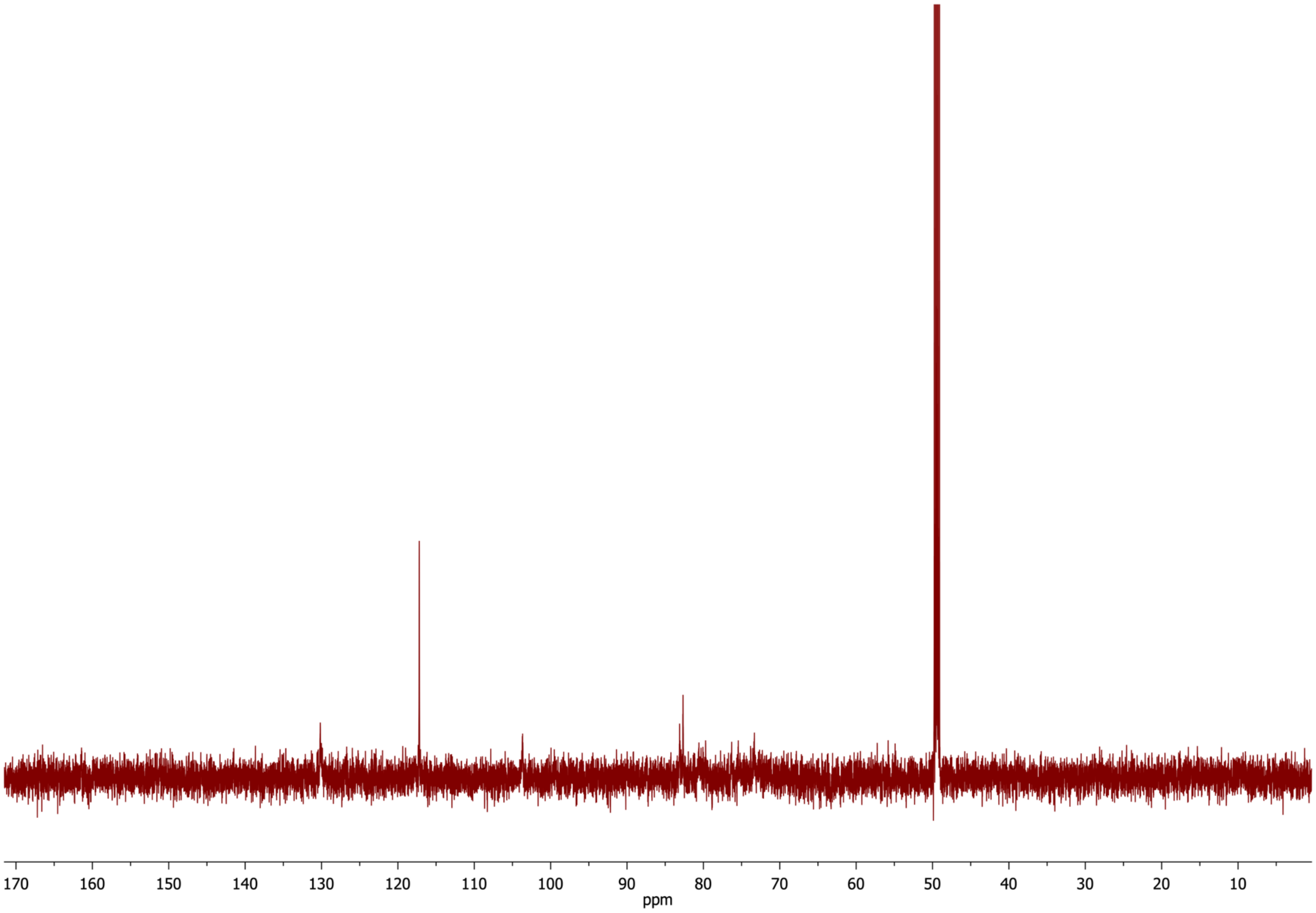

Figure S2. DEPT135 spectrum (CD_3OD, 500 MHz) of Compound **1**.

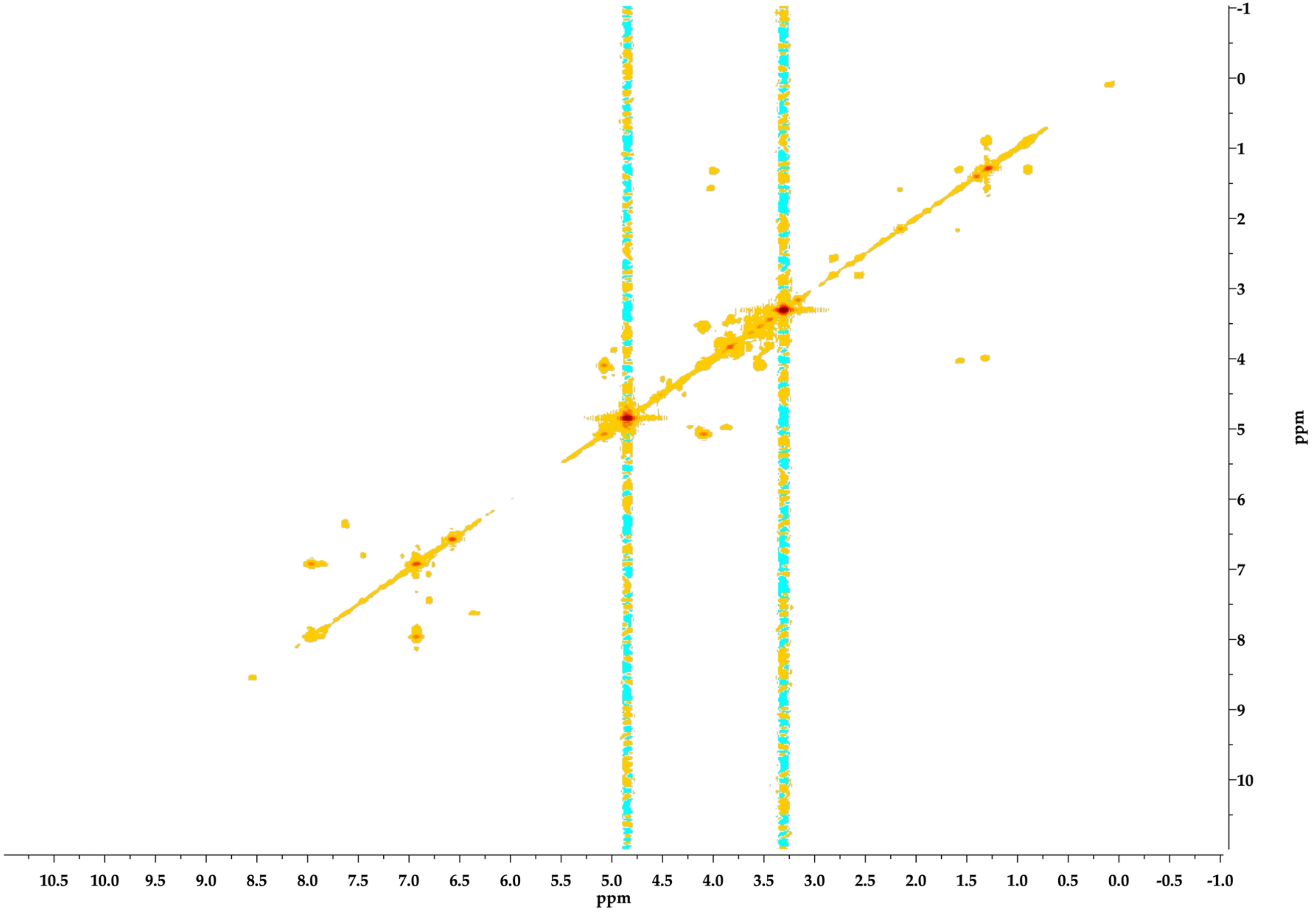

Figure S3. 2D ^{1}H, ^{1}H-Homonuclear Correlated Spectrum (COSY) of the Compound **1**.

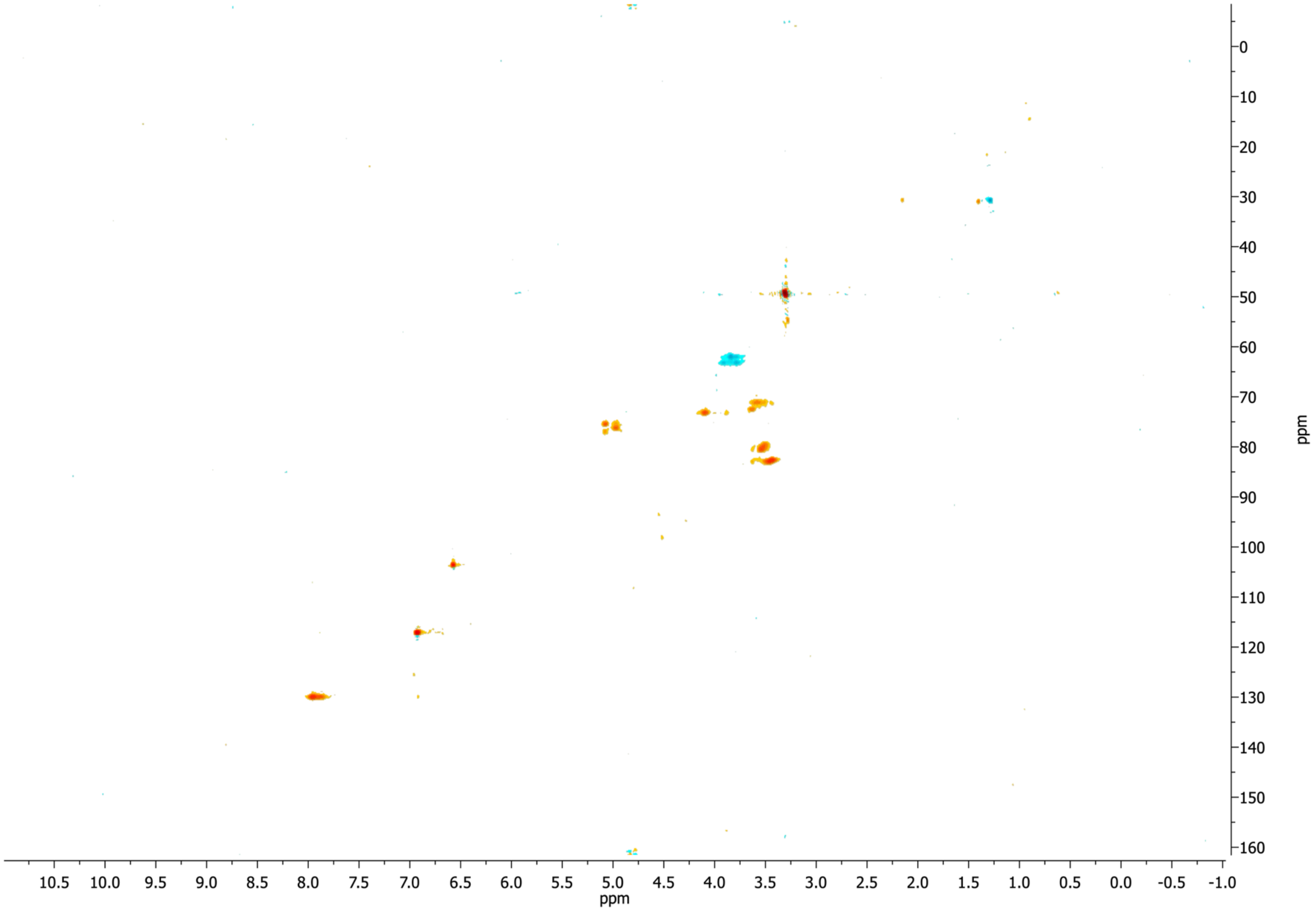

Figure S4. 2D ^{1}H, ^{13}C Heteronuclear Correlation (close range) NMR Spectrum (HSQC) of the Compound **1**.

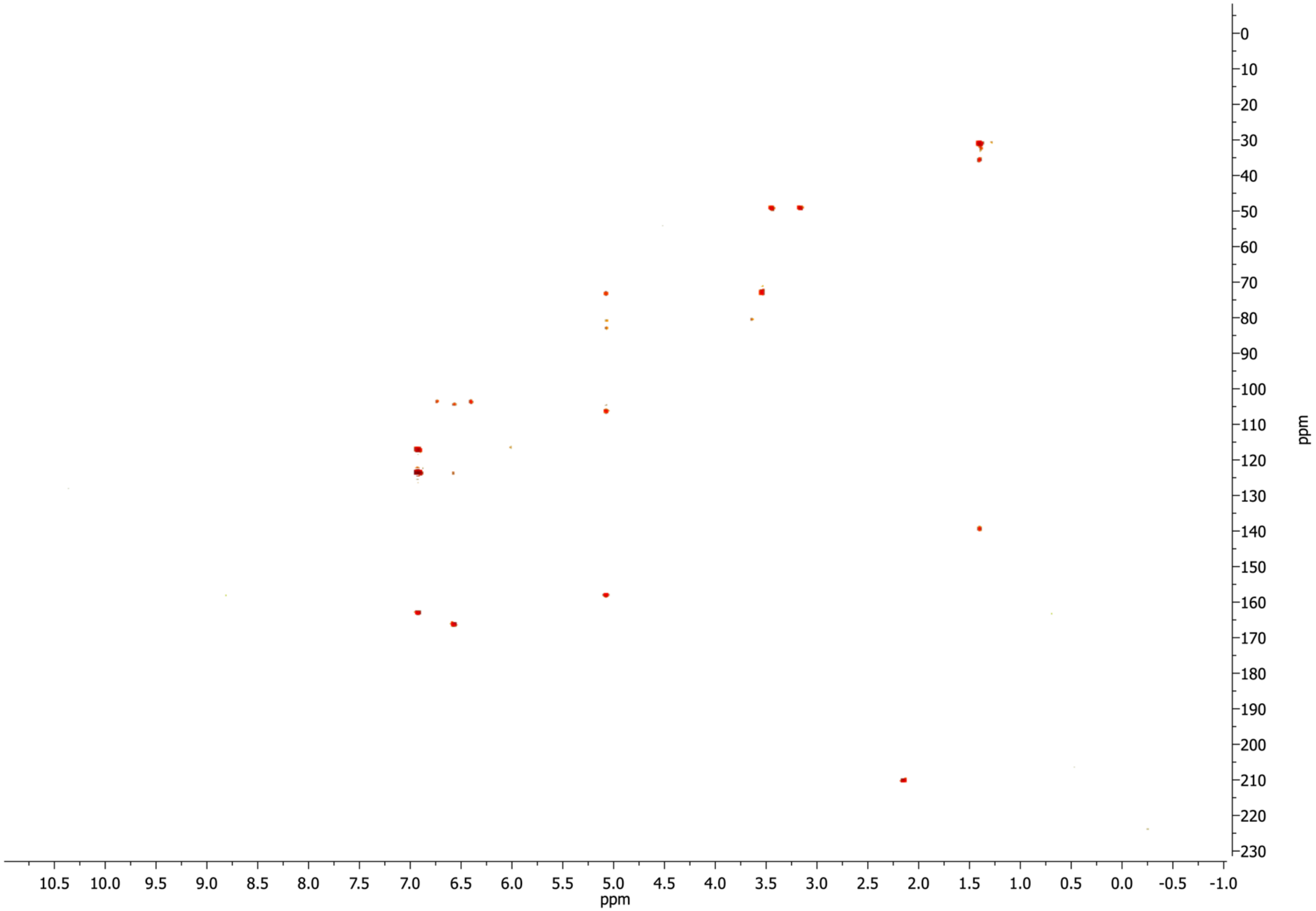

Figure S5. 2D ^{1}H, ^{13}C Heteronuclear Correlated (long-range) NMR Spectrum (HMBC) of the Compound **1**.

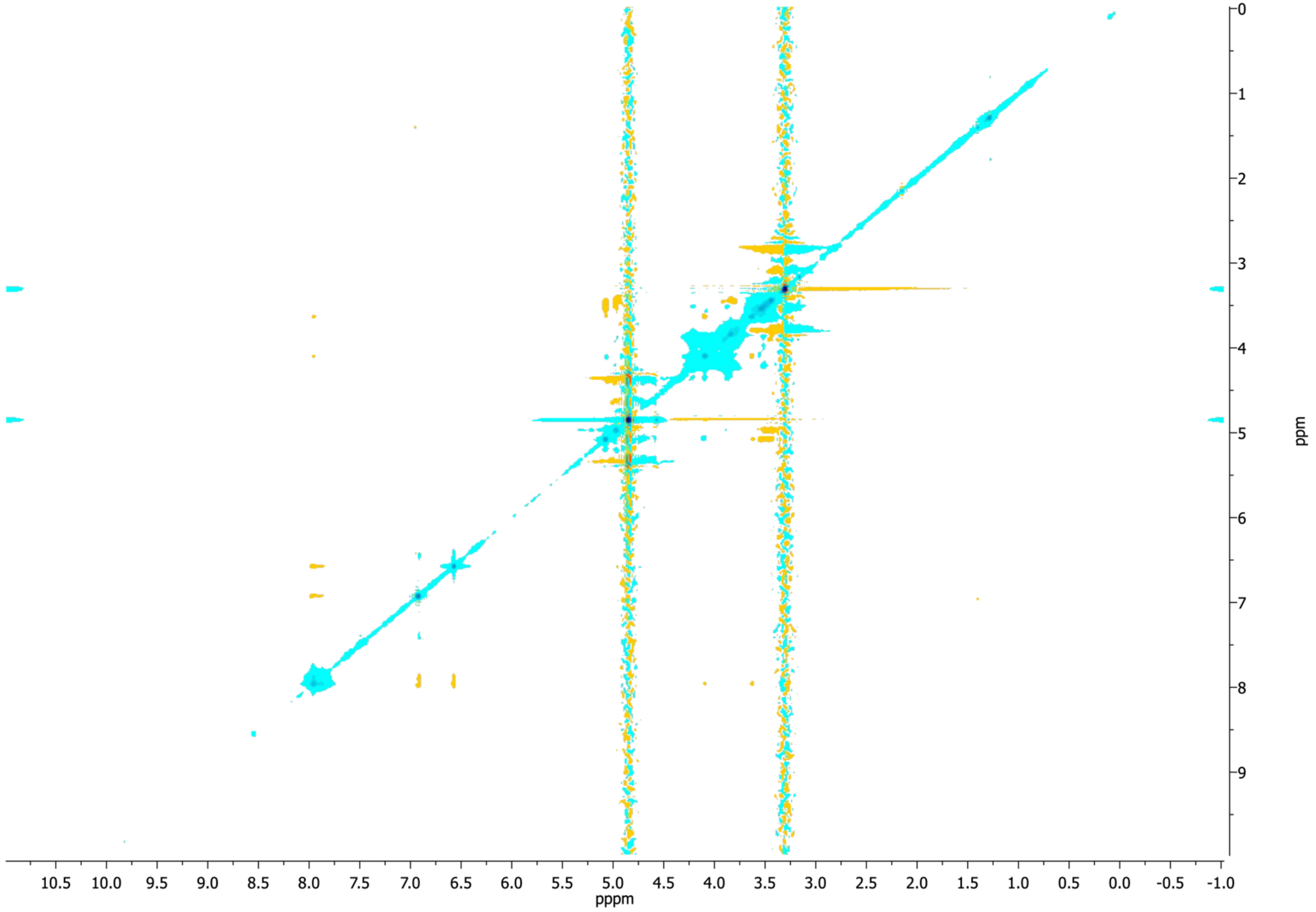

Figure S6. 2D Nuclear Overhauser Effect Spectroscopy Spectrum (NOESY) of the Compound **1**.

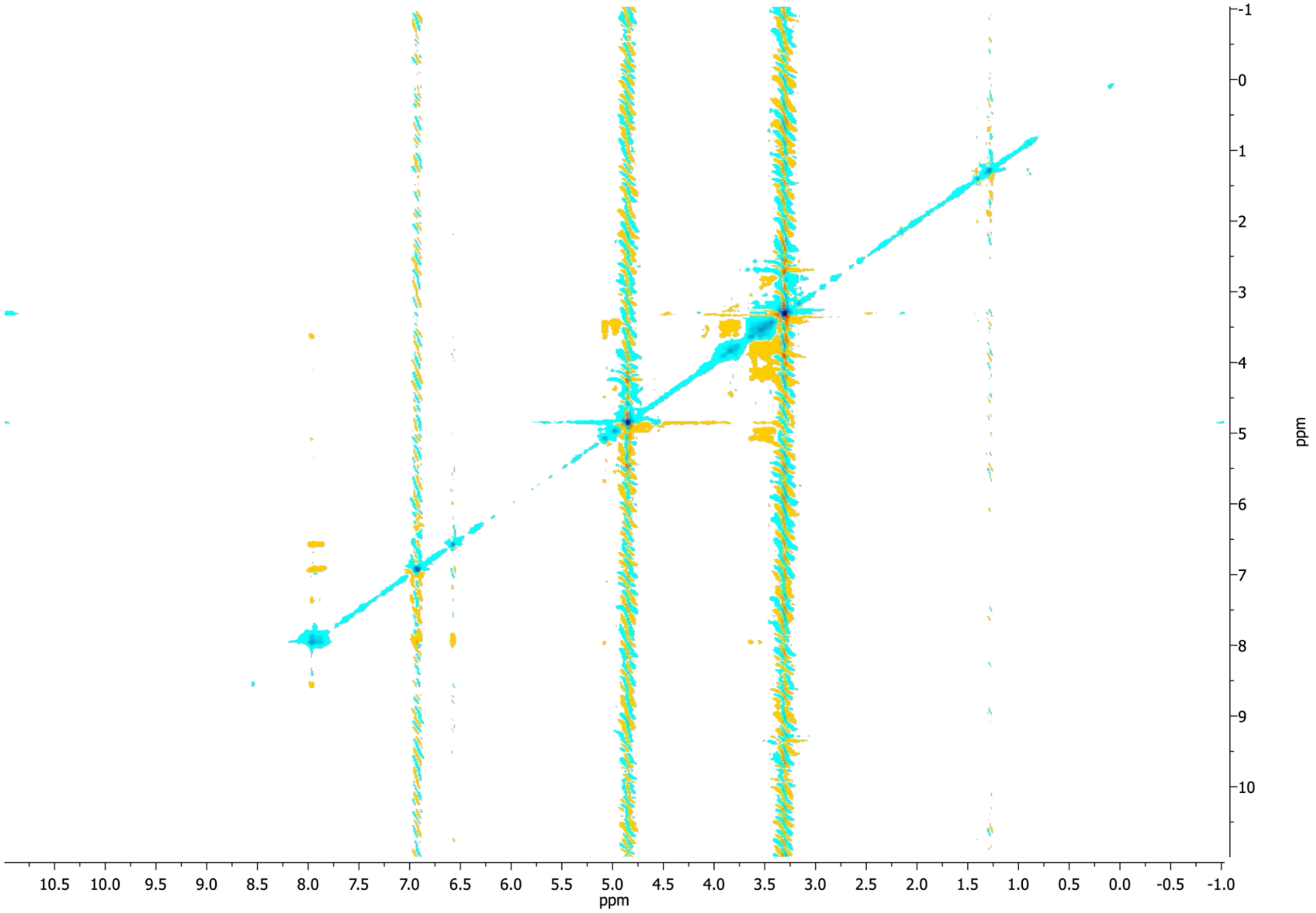

Figure S7. 2D Rotating-frame Overhauser Effect Spectroscopy Spectrum (ROESY) of the Compound **1**.

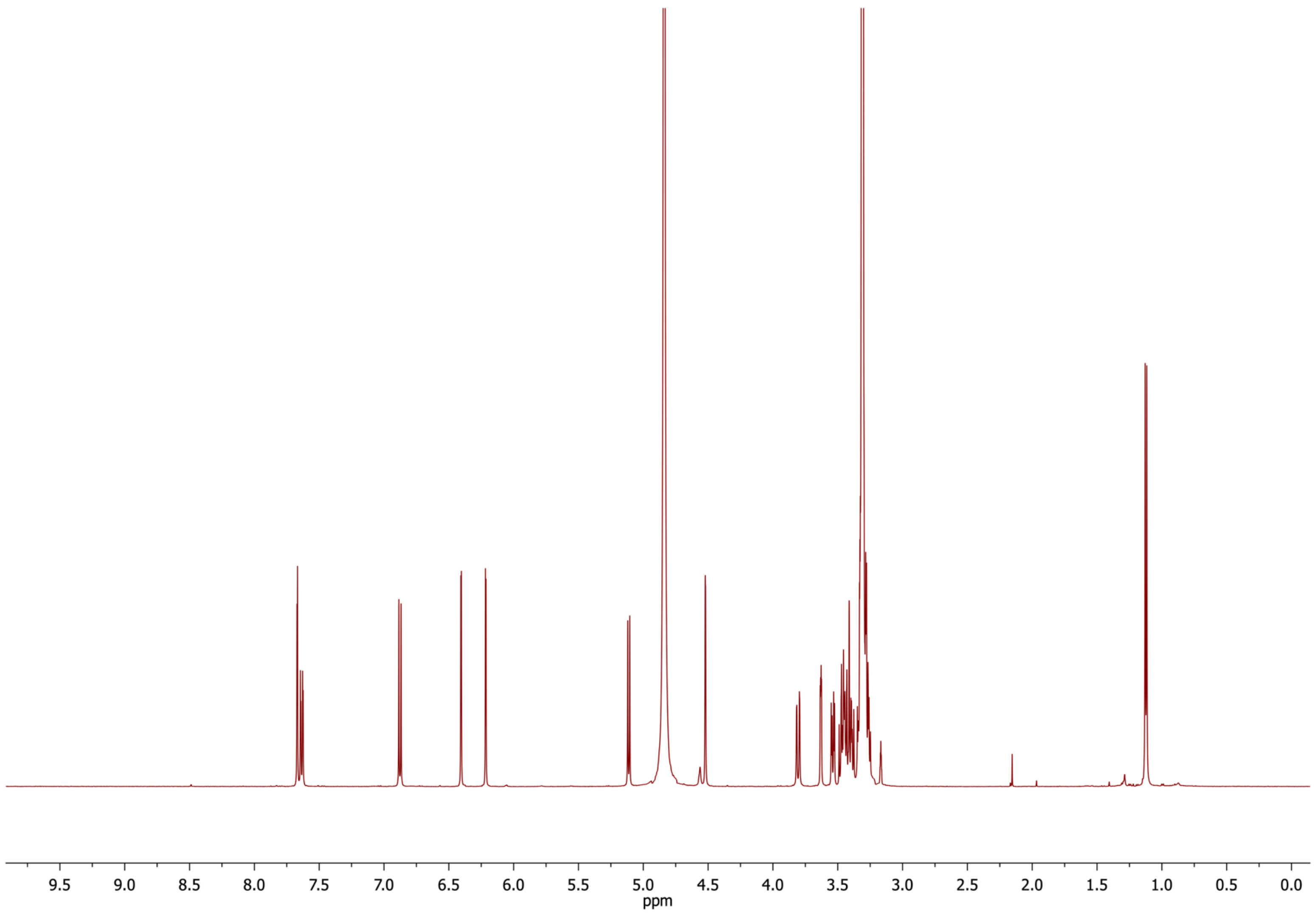

Figure S8. ^{1}H NMR spectrum of Compound **2** (CD_3OD, 500 MHz).

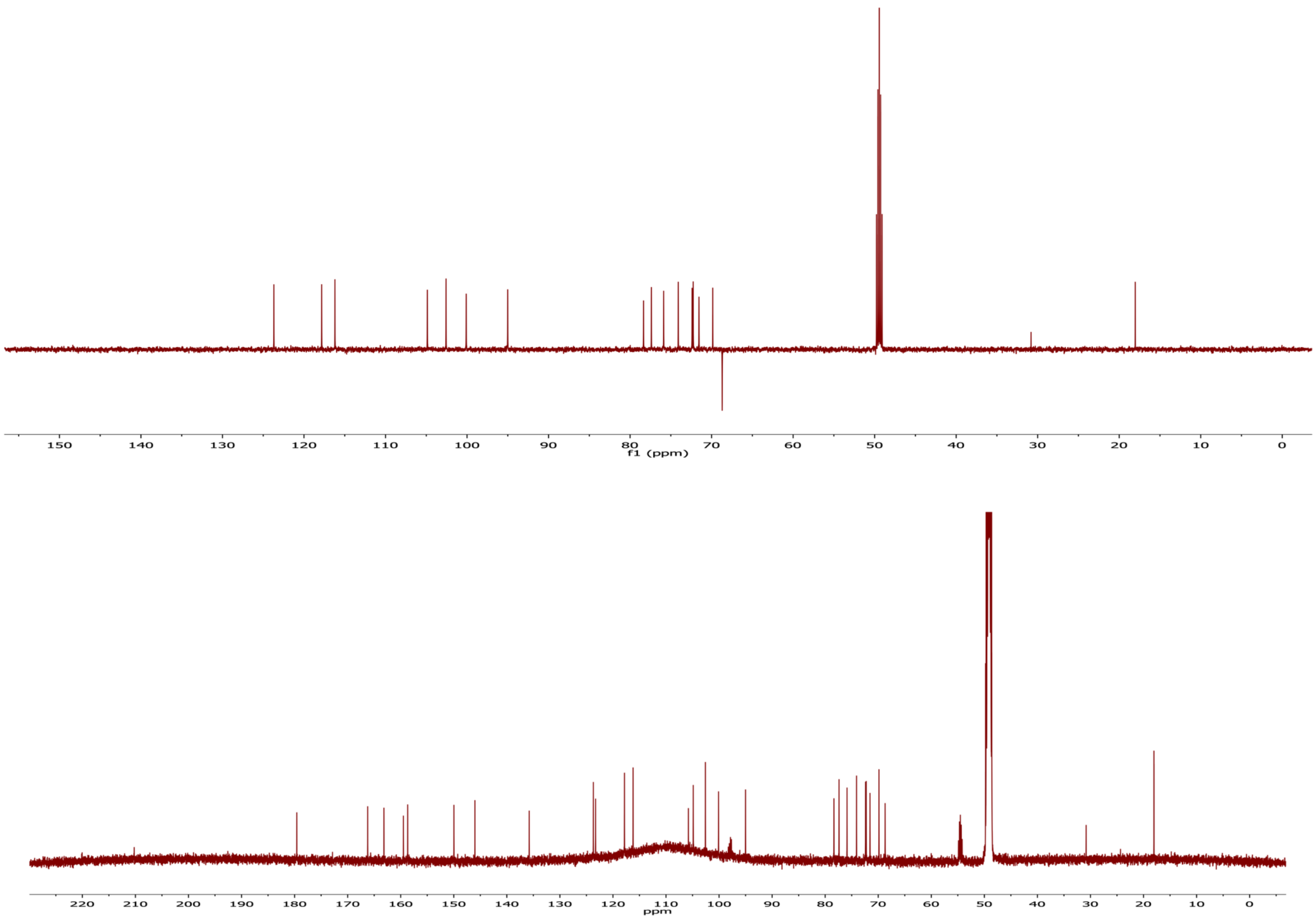

Figure S9. DEPT135 spectrum (CD_3OD, 500 MHz) and ^{13}C NMR spectrum (CD_3OD, 125 MHz) of Compound **2**.

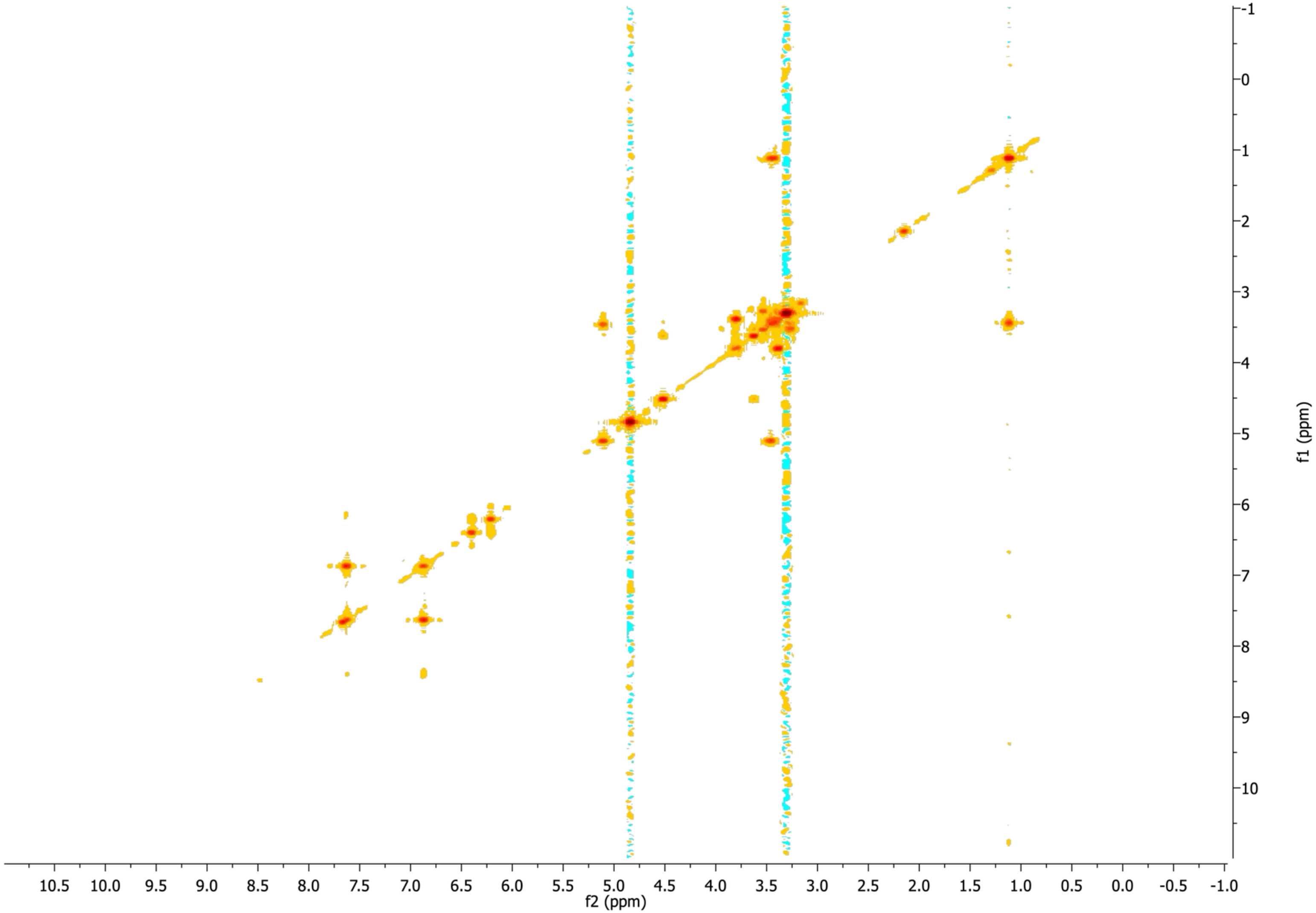

Figure S10. 2D ¹H, ¹H-Homonuclear Correlated Spectrum (COSY) of the Compound **2**.

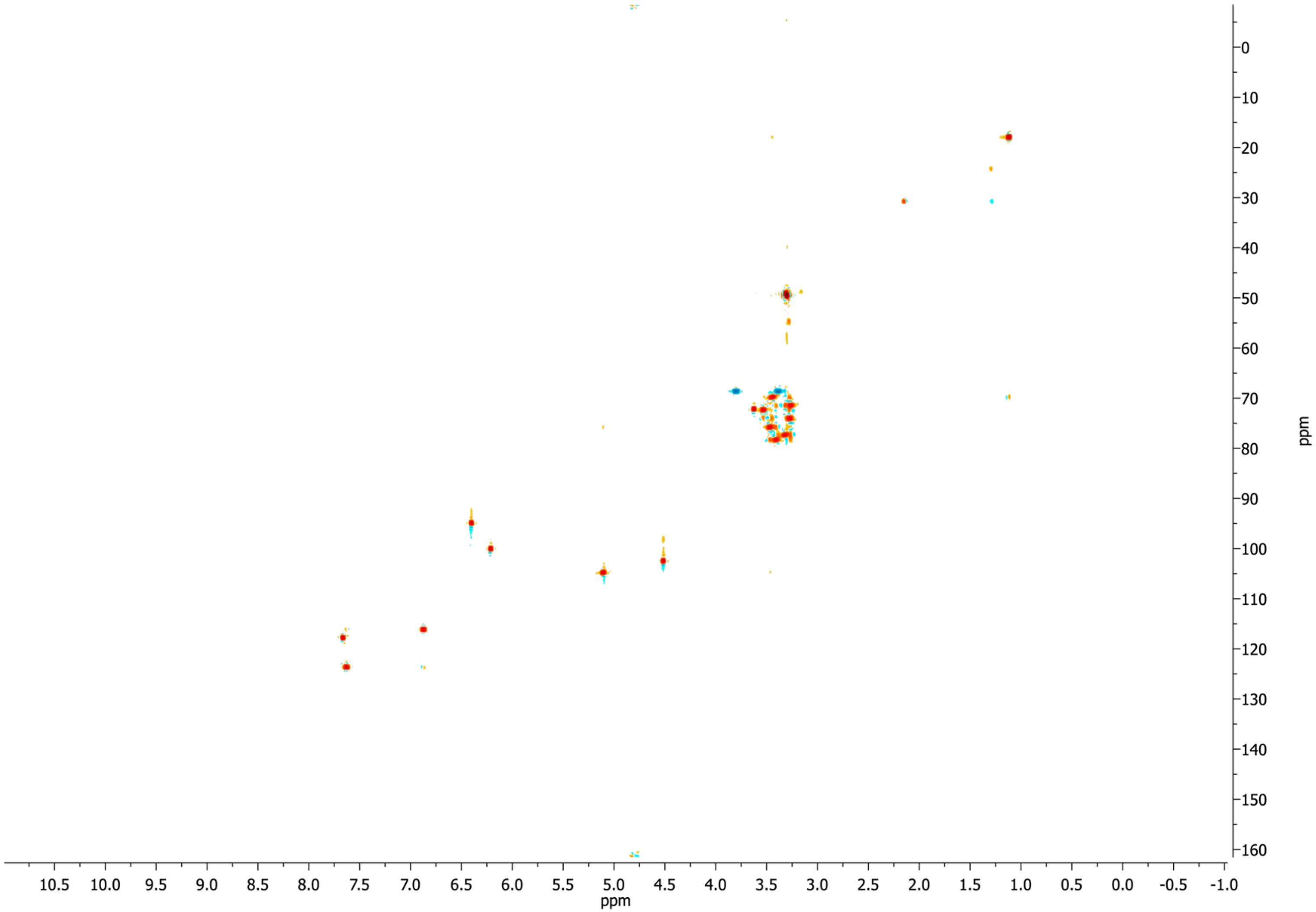

Figure S11. 2D ¹H, ¹³C Heteronuclear Correlation (close range) NMR Spectrum (HSQC) of the Compound **2**.

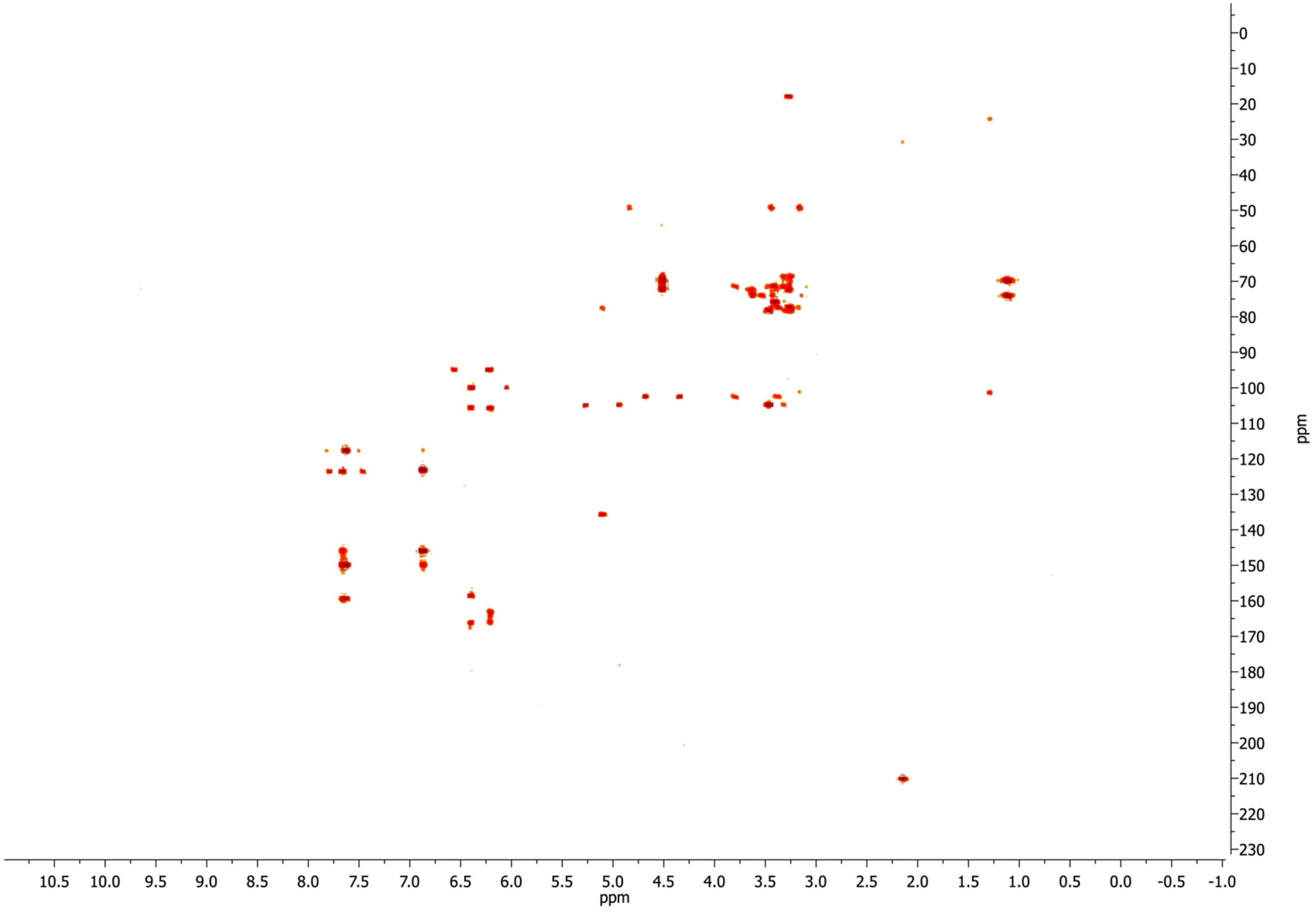

Figure S12. 2D ^{1}H, [13]C Heteronuclear Correlated (long-range) NMR Spectrum (HMBC) of the Compound **2**.

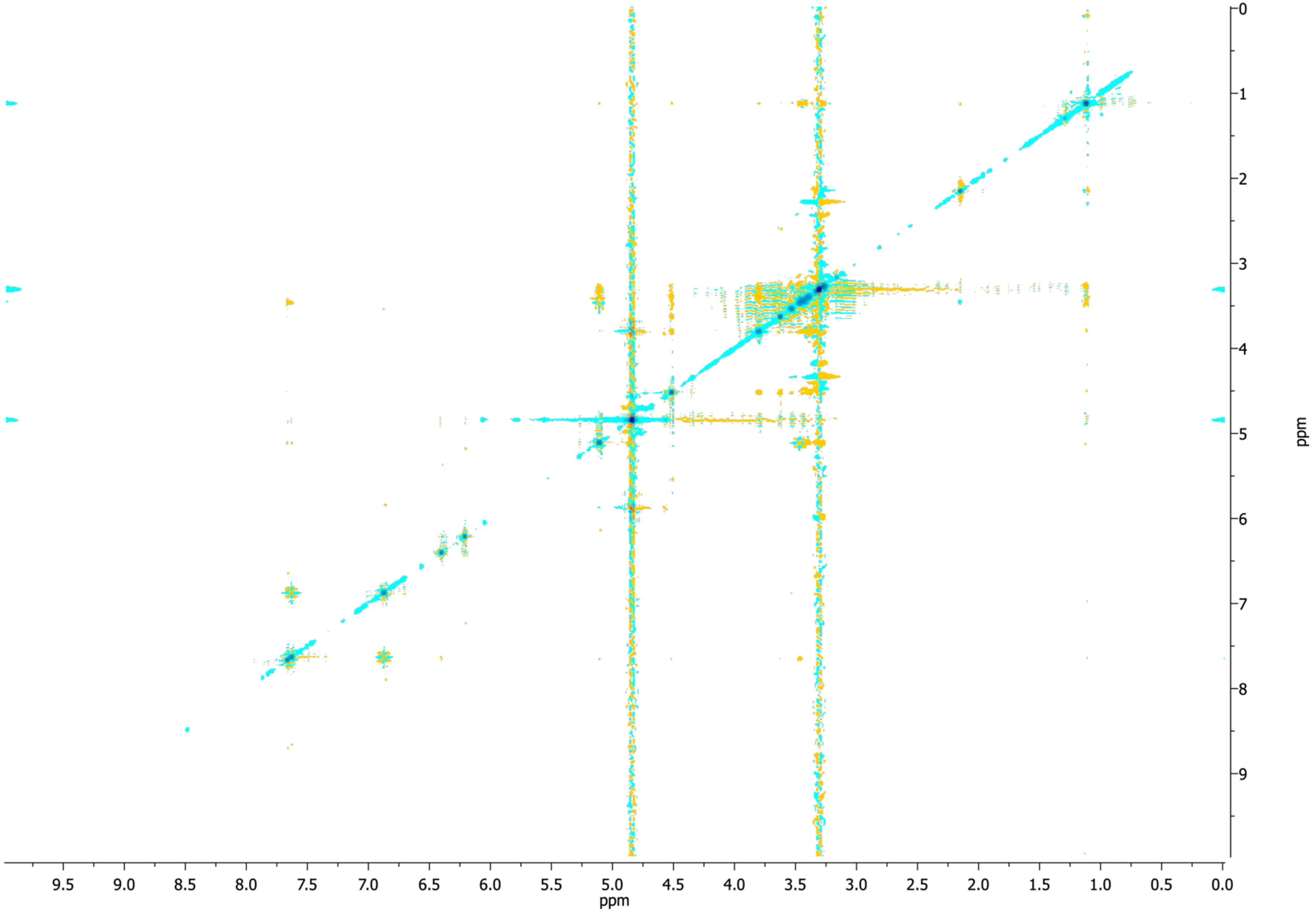

Figure S13. 2D Nuclear Overhauser Effect Spectroscopy Spectrum (NOESY) of the Compound **2**.

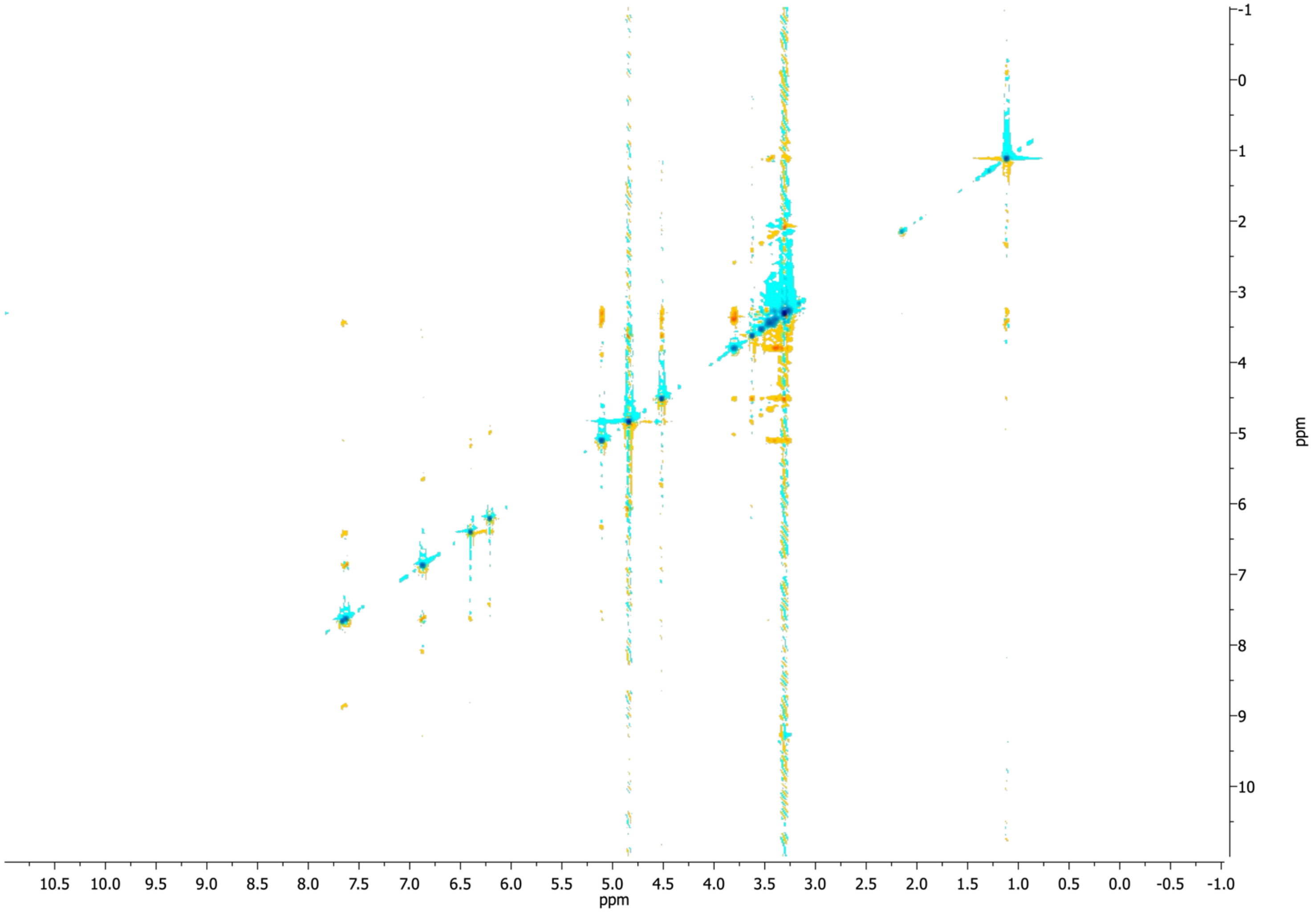

Figure S14. 2D Rotating-frame Overhauser Effect Spectroscopy Spectrum (ROESY) of the Compound **2**.

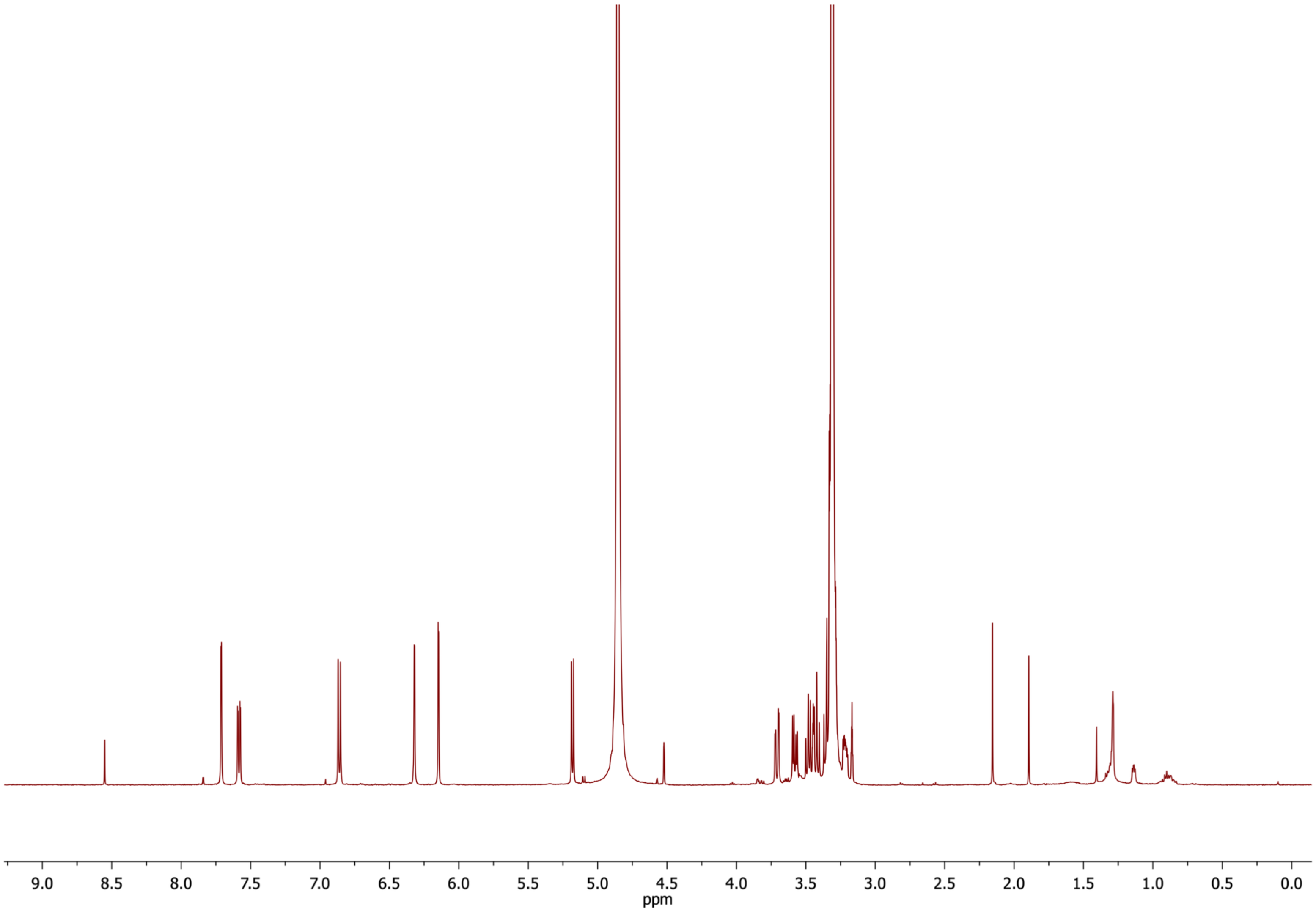

Figure S15. ¹H NMR spectrum of Compound **3** (CD_3OD, 500 MHz).

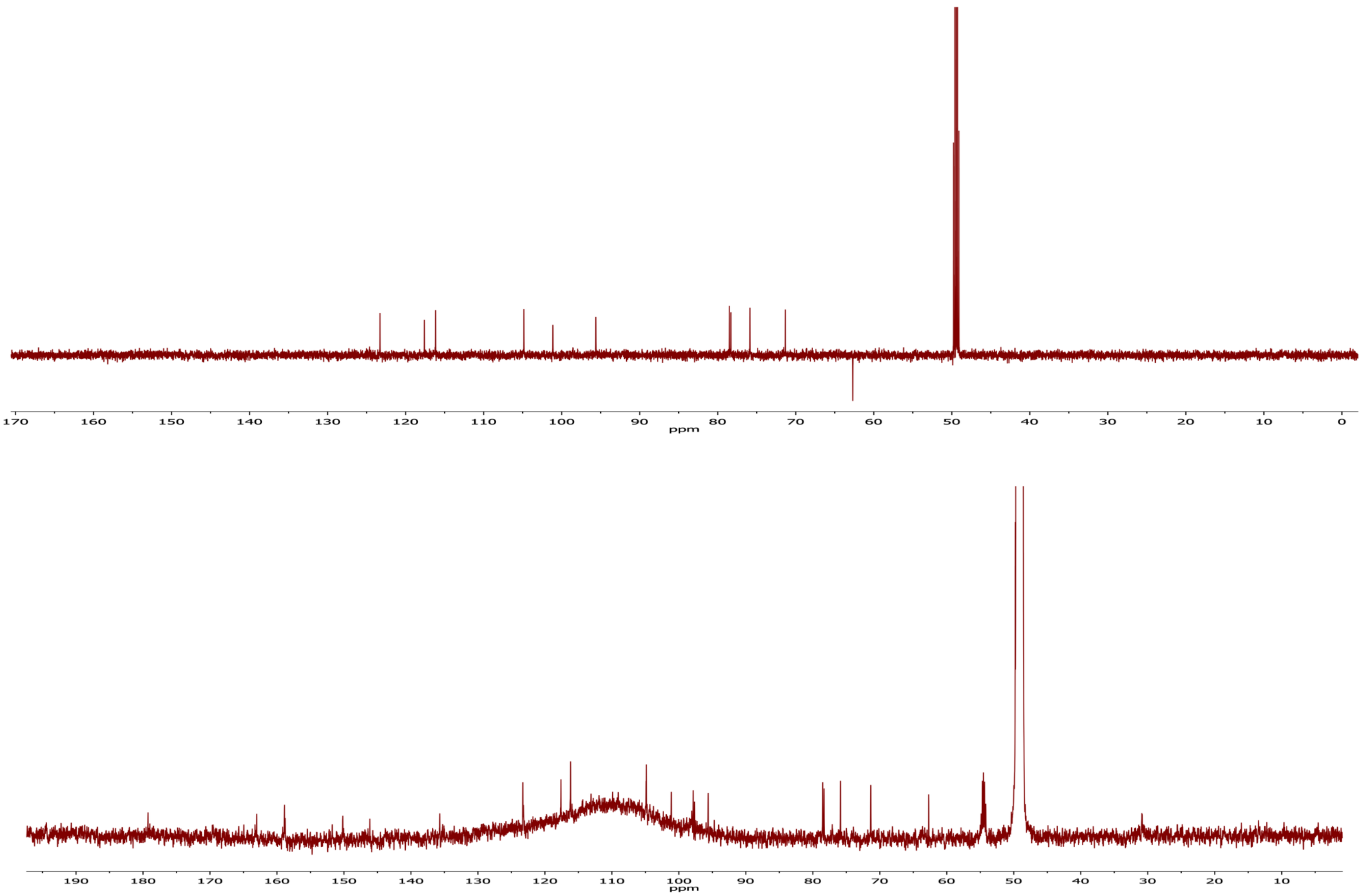

Figure S16. DEPT135 spectrum (CD_3OD, 500 MHz) and ^{13}C NMR spectrum (CD_3OD, 125 MHz) of Compound **3**.

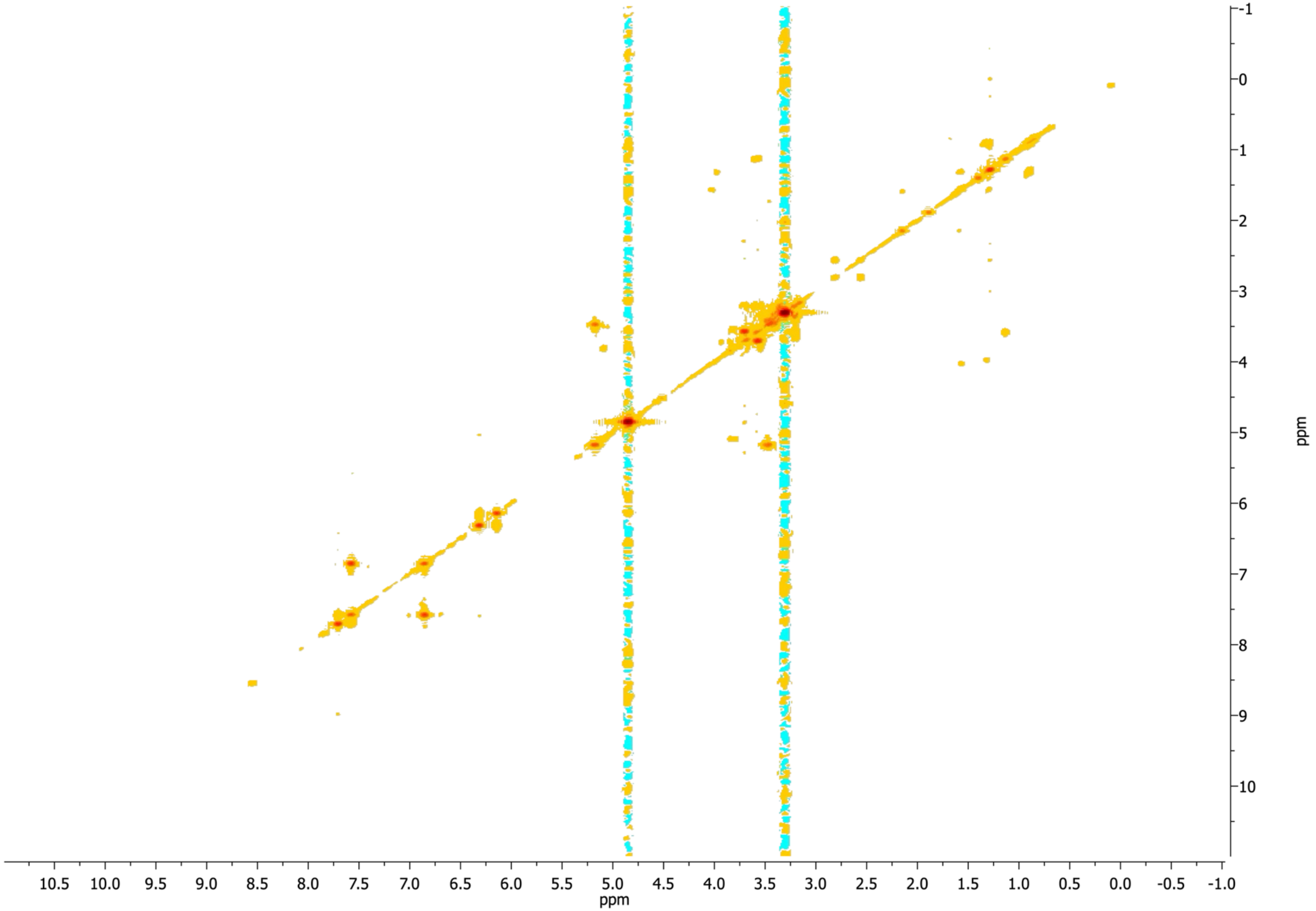

Figure S17. 2D ^{1}H, ^{1}H-Homonuclear Correlated Spectrum (COSY) of the Compound **3**.

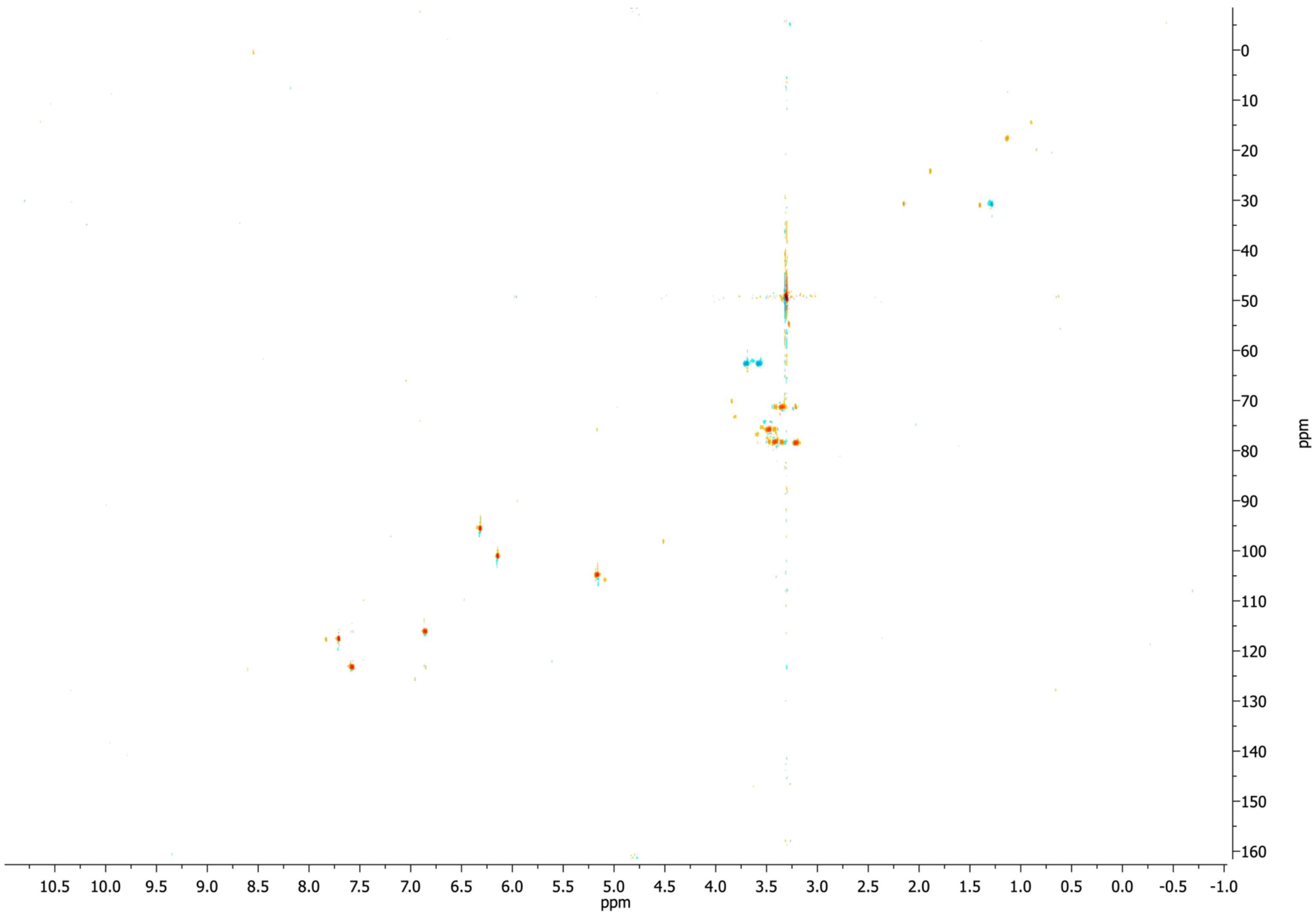

Figure S18. 2D ^{1}H, [13]C Heteronuclear Correlation (close range) NMR Spectrum (HSQC) of the Compound **3**.

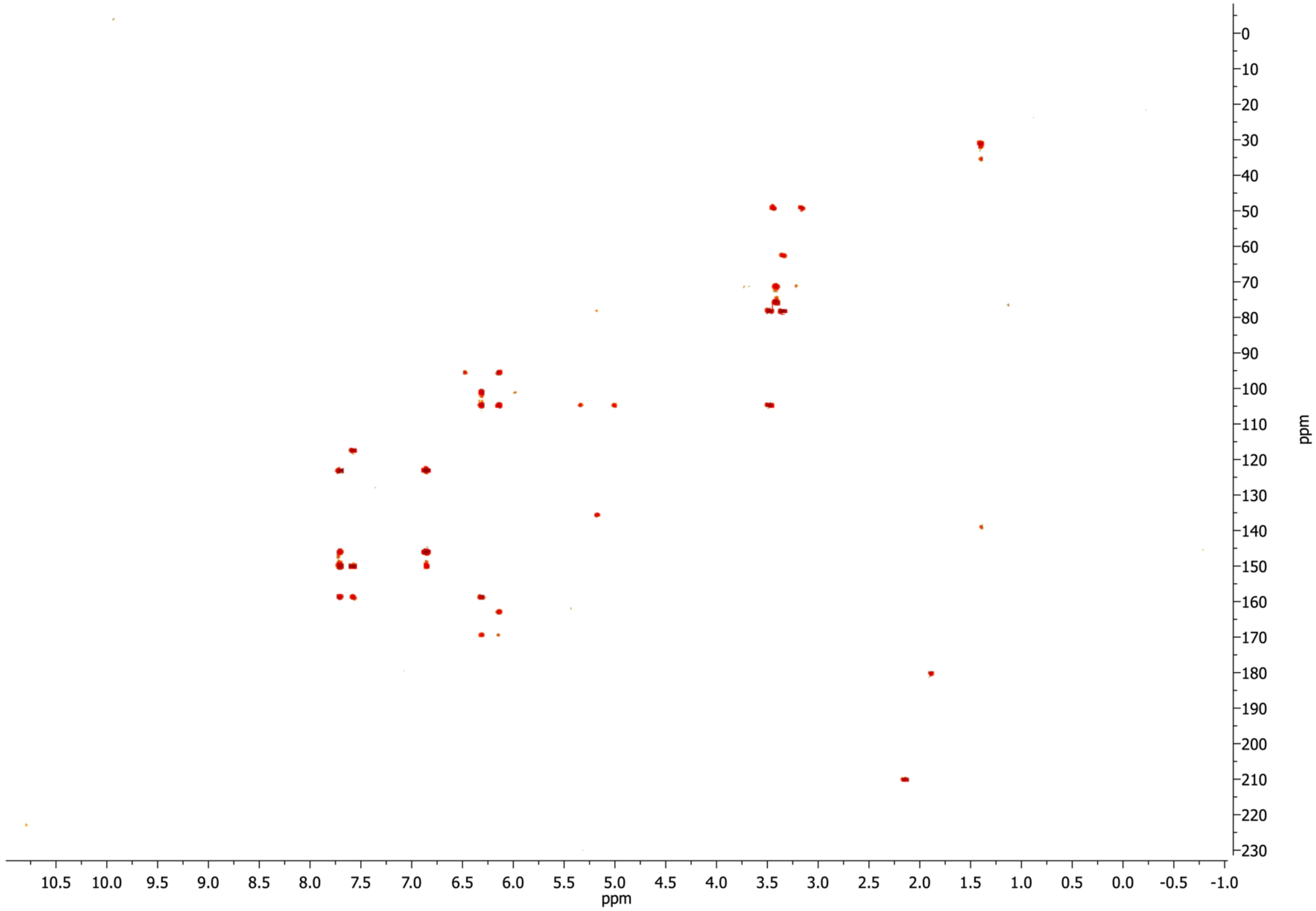

Figure S19. 2D ^{1}H, ^{13}C Heteronuclear Correlated (long-range) NMR Spectrum (HMBC) of the Compound **3**.

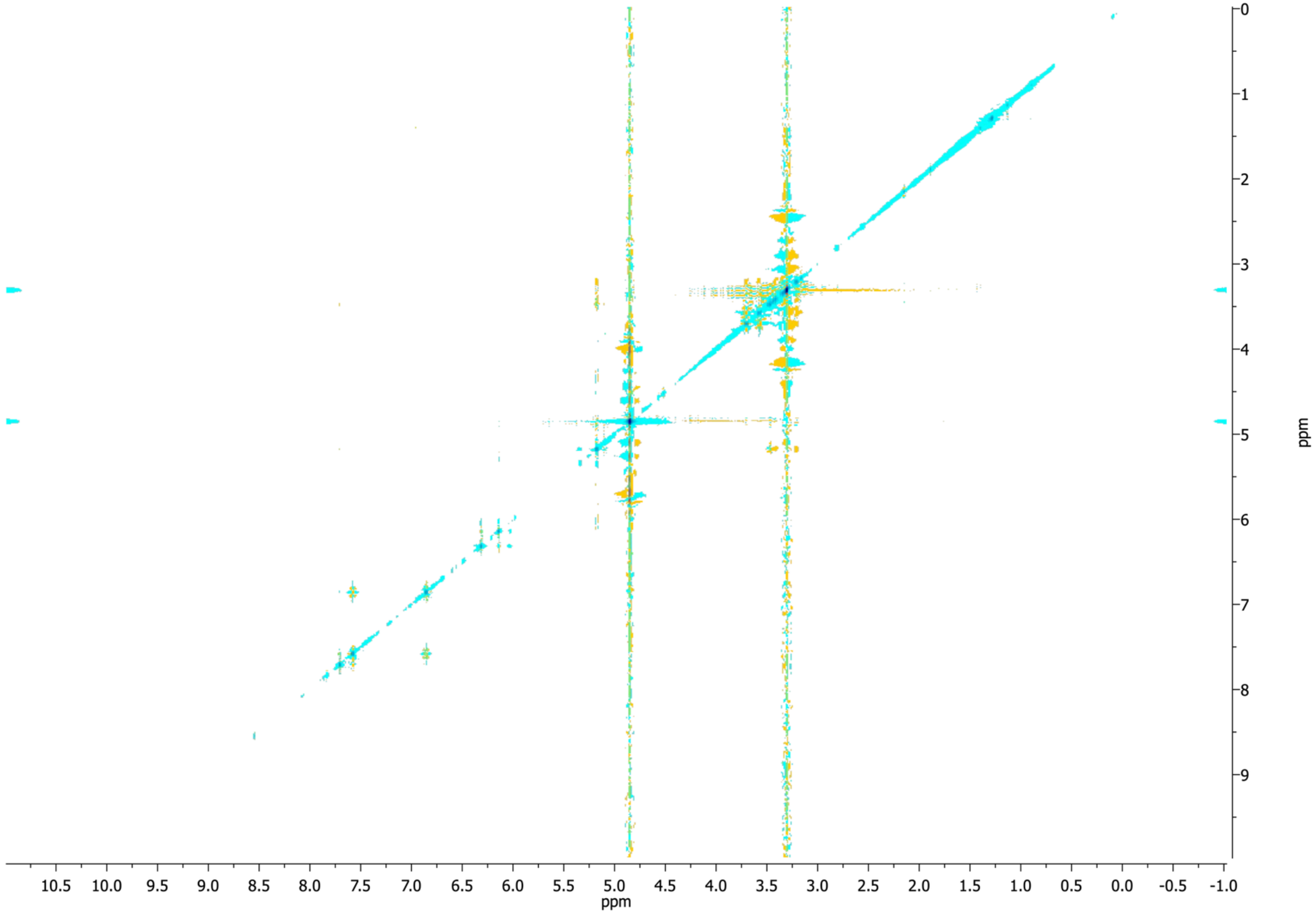

Figure S20. 2D Nuclear Overhauser Effect Spectroscopy Spectrum (NOESY) of the Compound **3**.

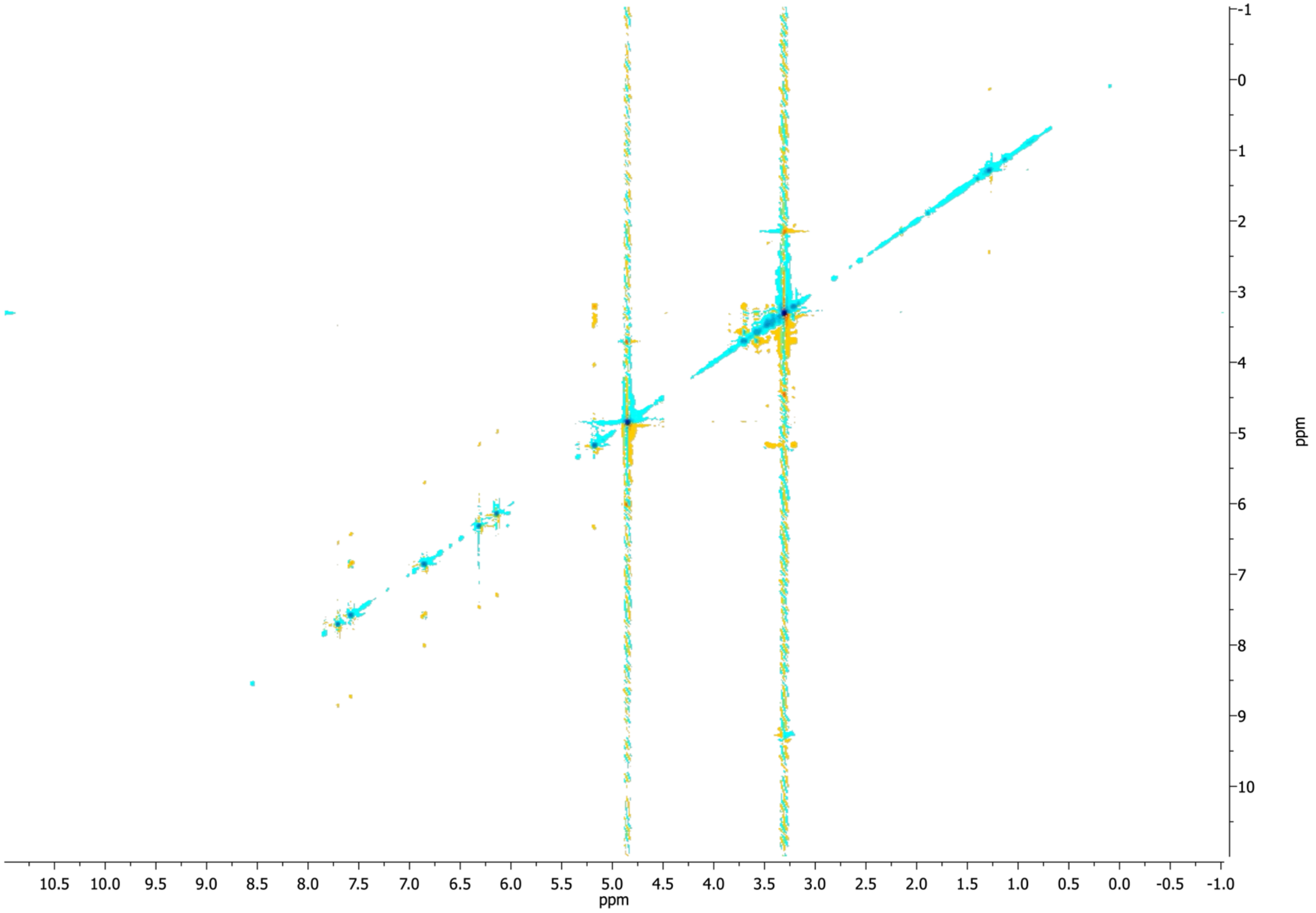

Figure S21. 2D Rotating-frame Overhauser Effect Spectroscopy Spectrum (ROESY) of the Compound **3**.

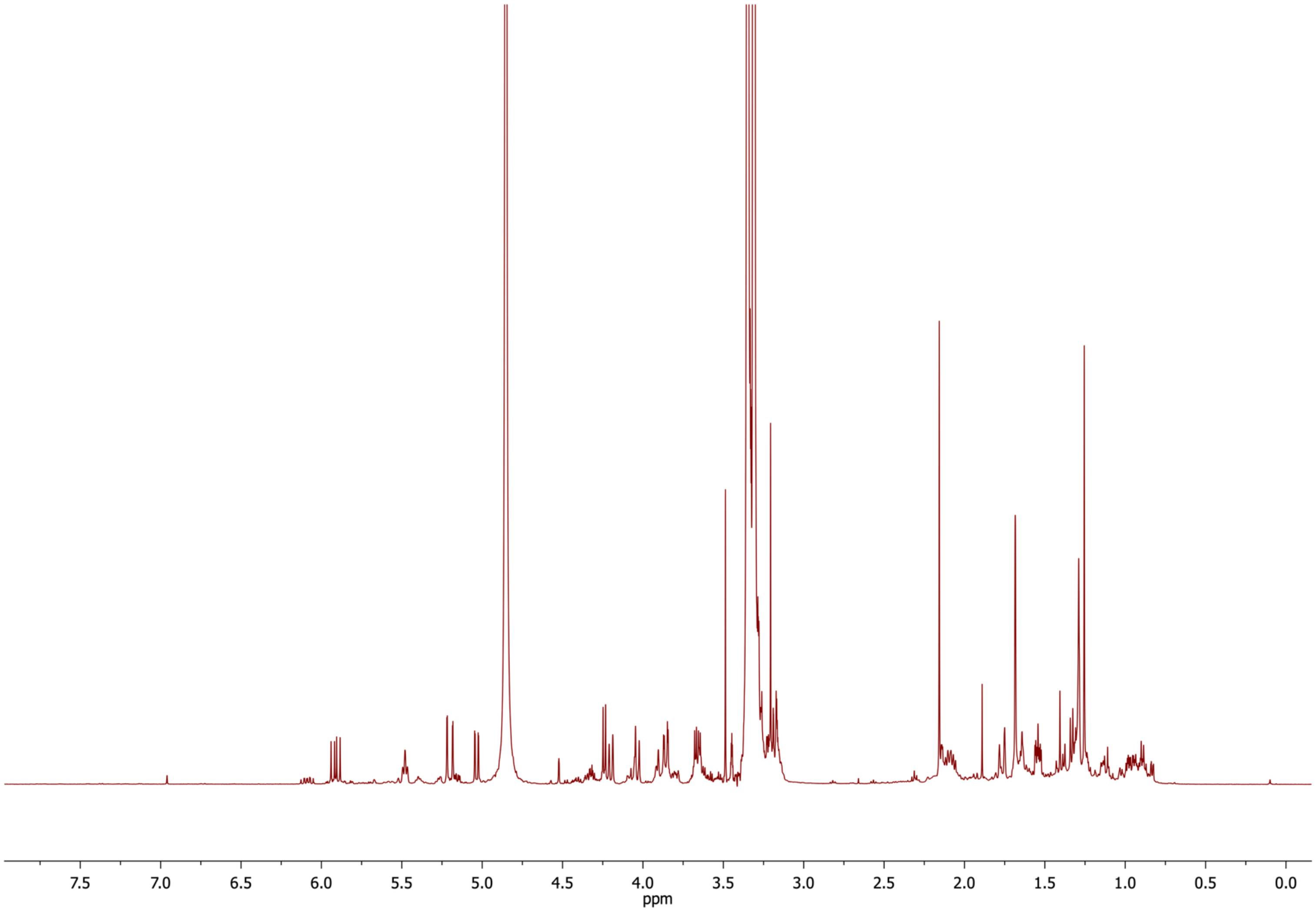

Figure S22. ^{1}H NMR spectrum of Compound **4** (CD_3OD, 500 MHz).

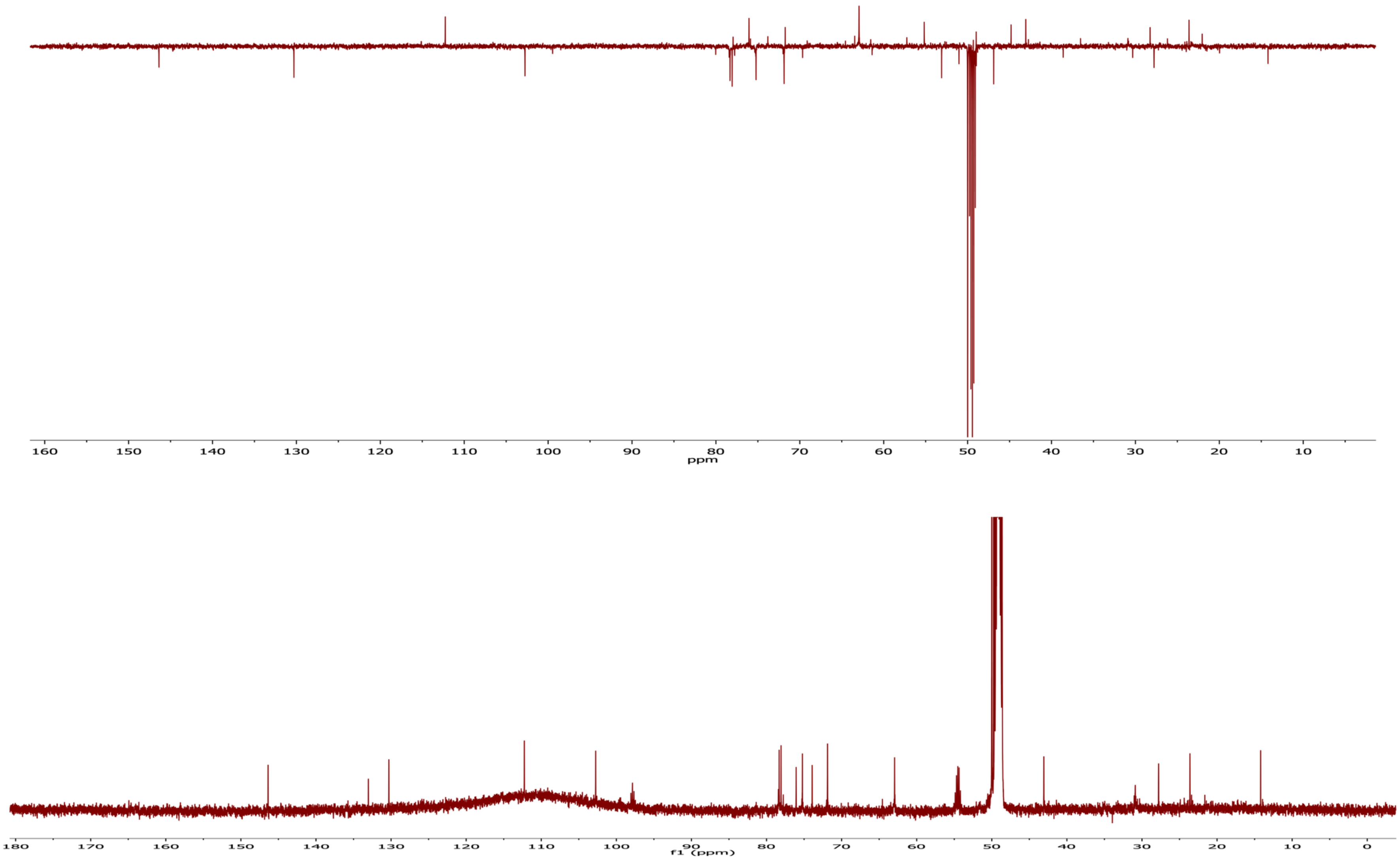

Figure S23. DEPT135 spectrum (CD_3OD, 500 MHz) and ^{13}C NMR spectrum (CD_3OD, 125 MHz) of Compound **4**.

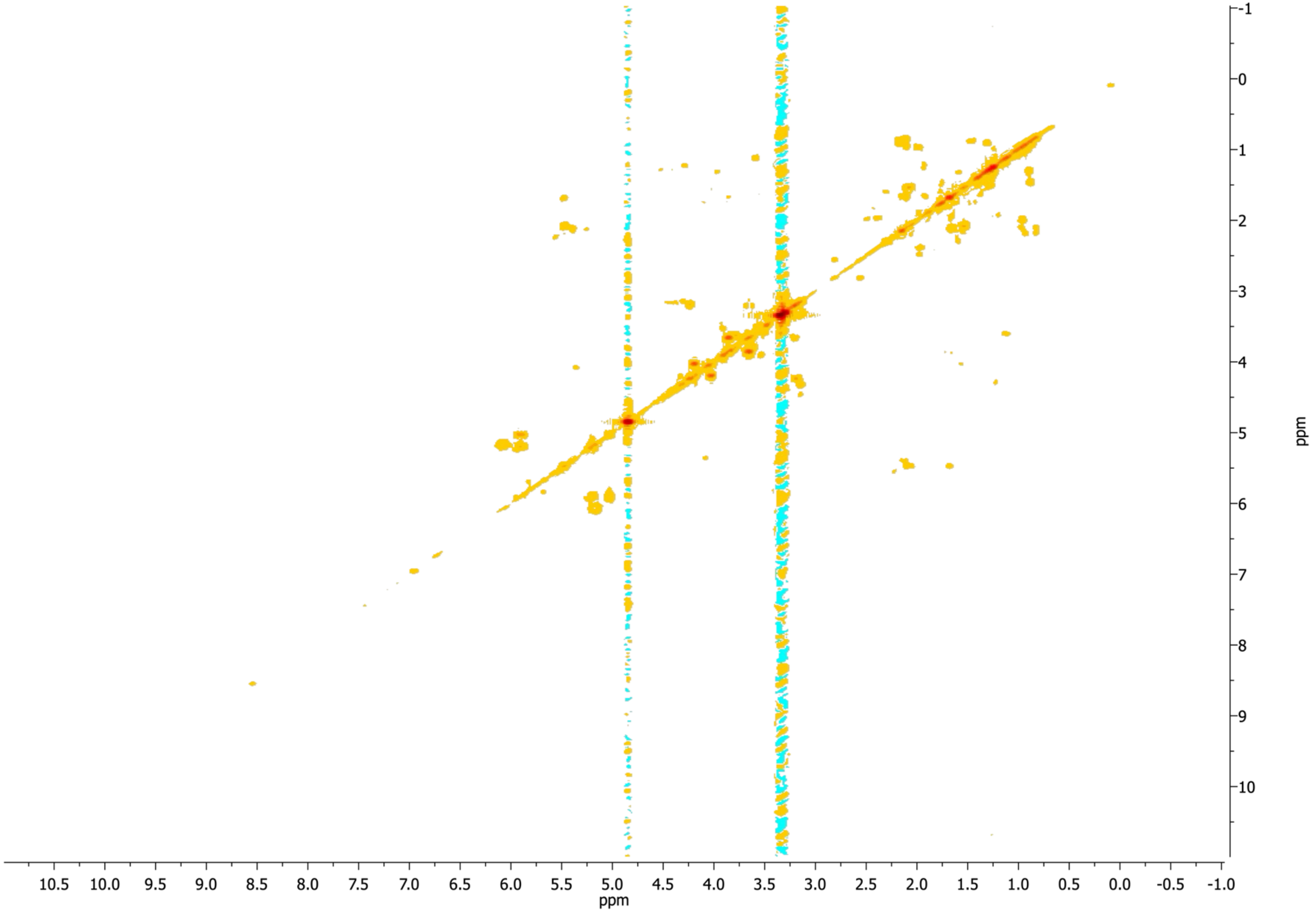

Figure S24. 2D ^{1}H, ^{1}H-Homonuclear Correlated Spectrum (COSY) of the Compound **4**.

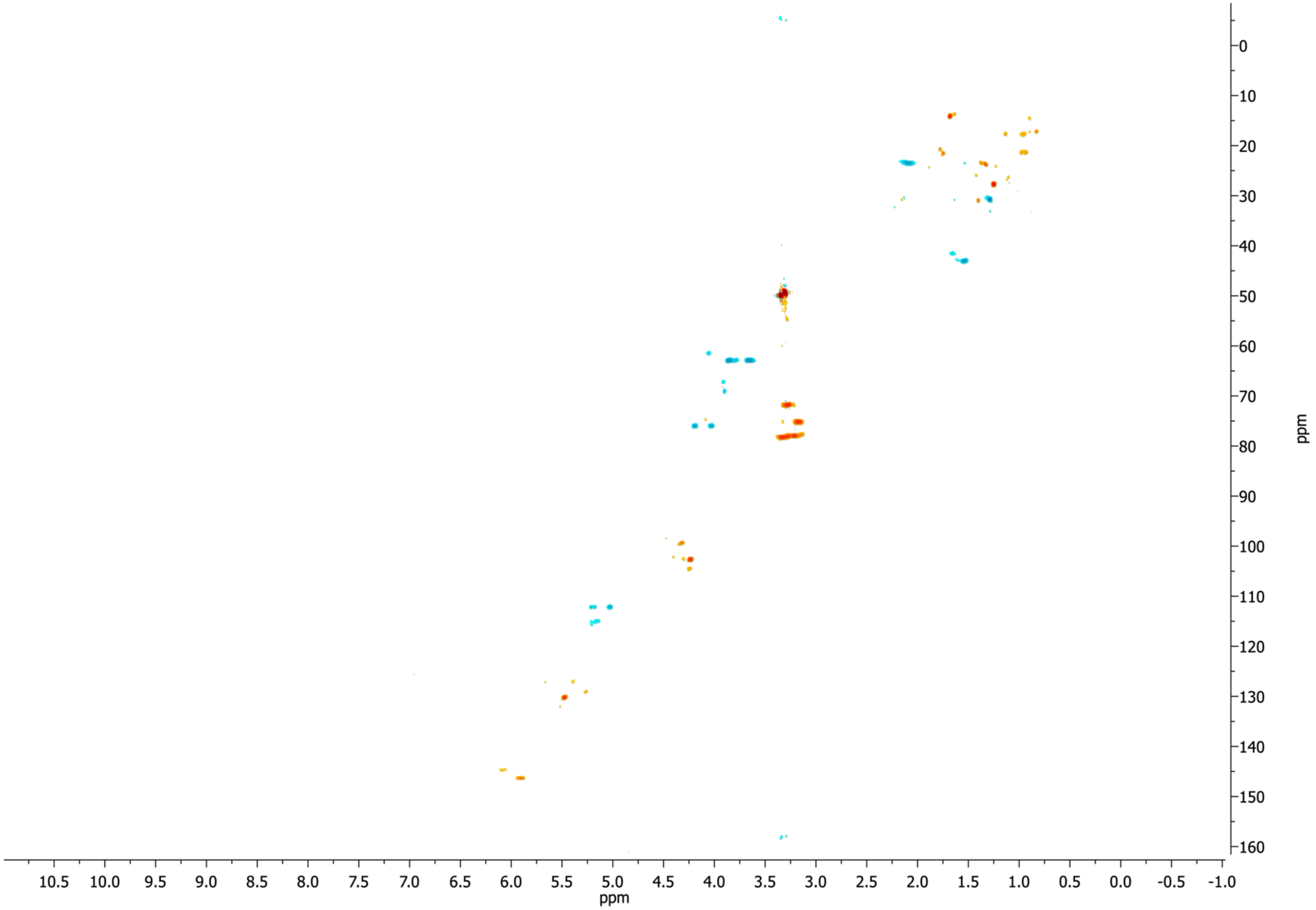

Figure S25. 2D ^{1}H, ^{13}C Heteronuclear Correlation (close range) NMR Spectrum (HSQC) of the Compound **4**.

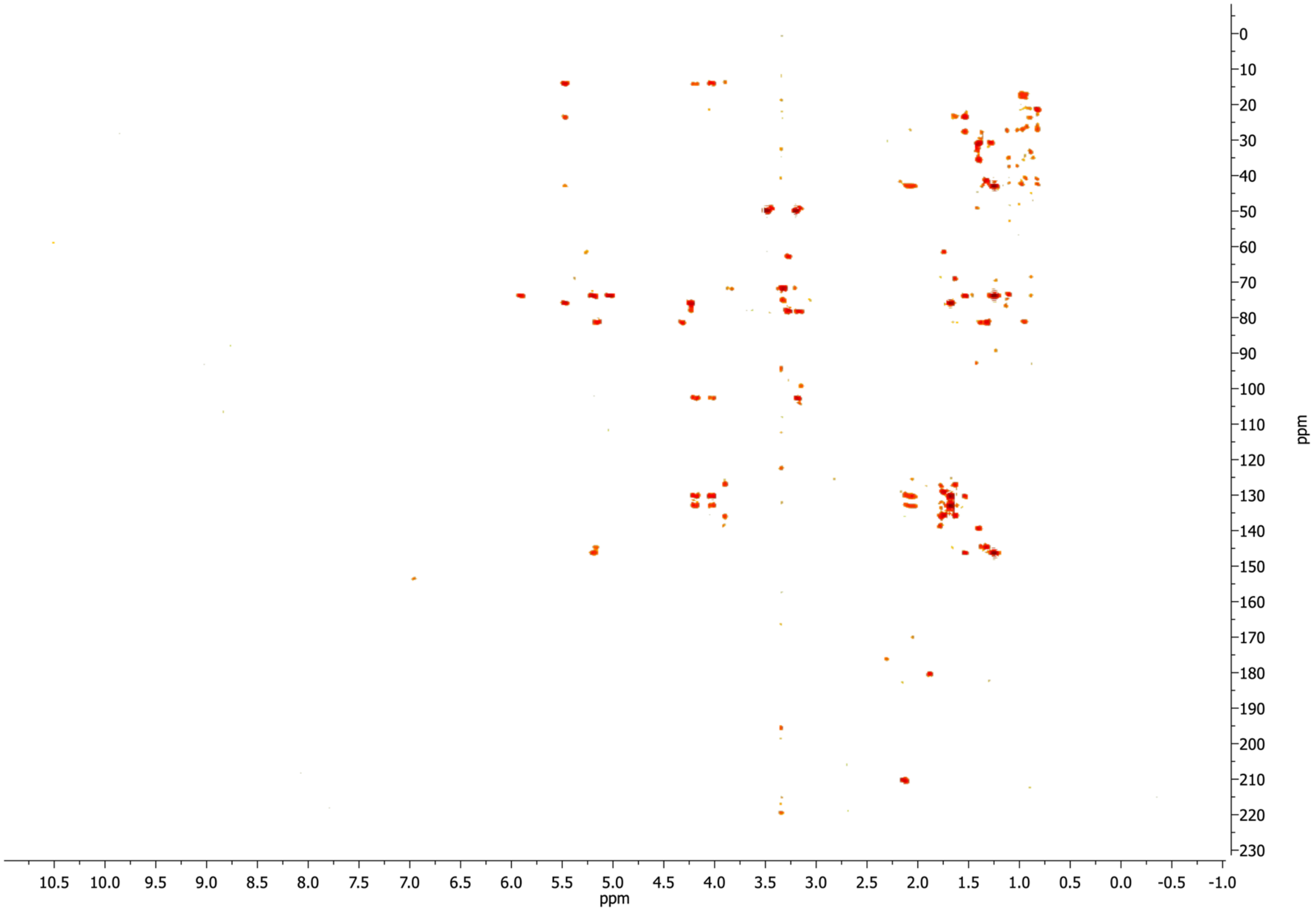

Figure S26. 2D ^{1}H, [13]C Heteronuclear Correlated (long-range) NMR Spectrum (HMBC) of the Compound **4**.

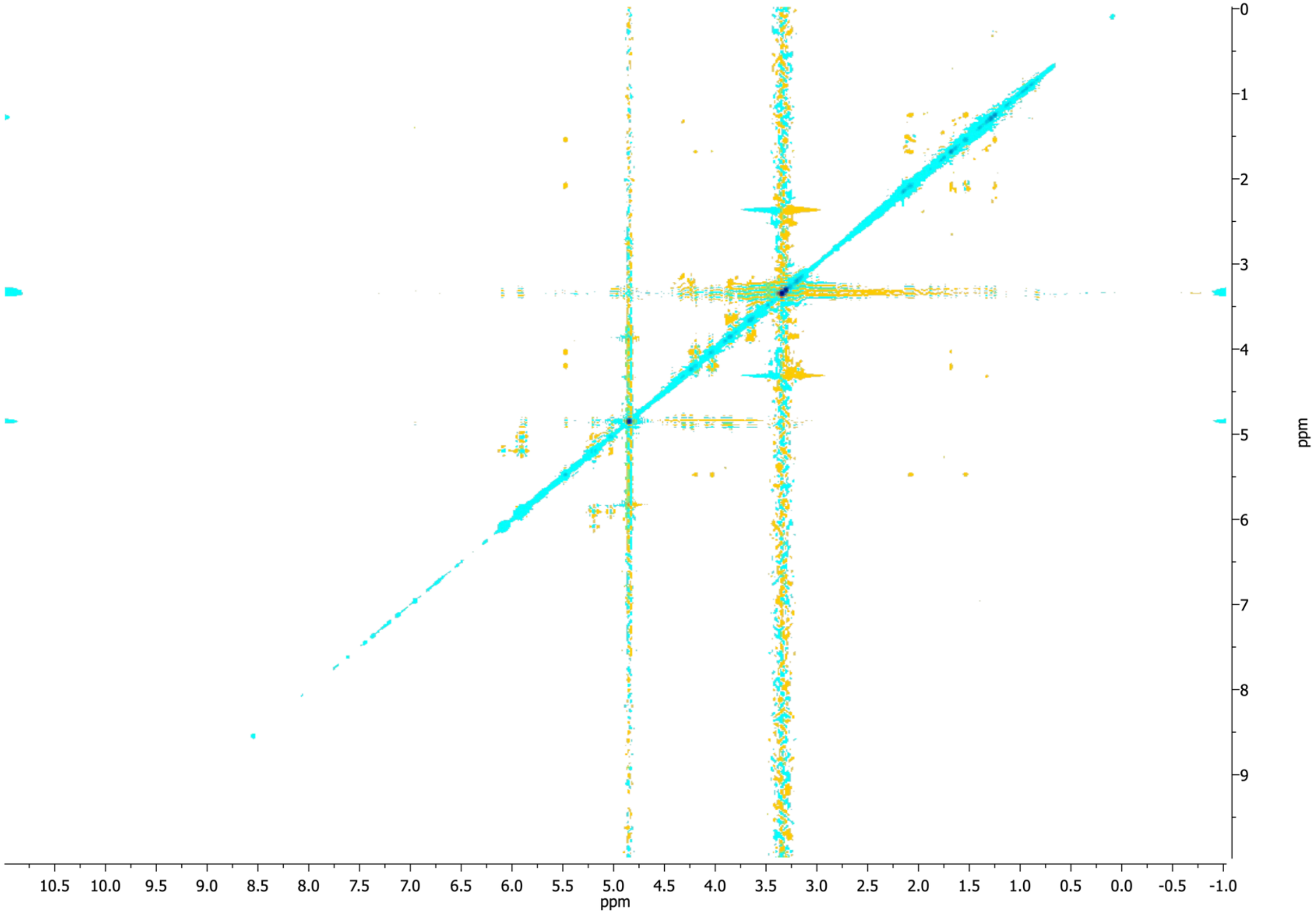

Figure S27. 2D Nuclear Overhauser Effect Spectroscopy Spectrum (NOESY) of the Compound **4**.

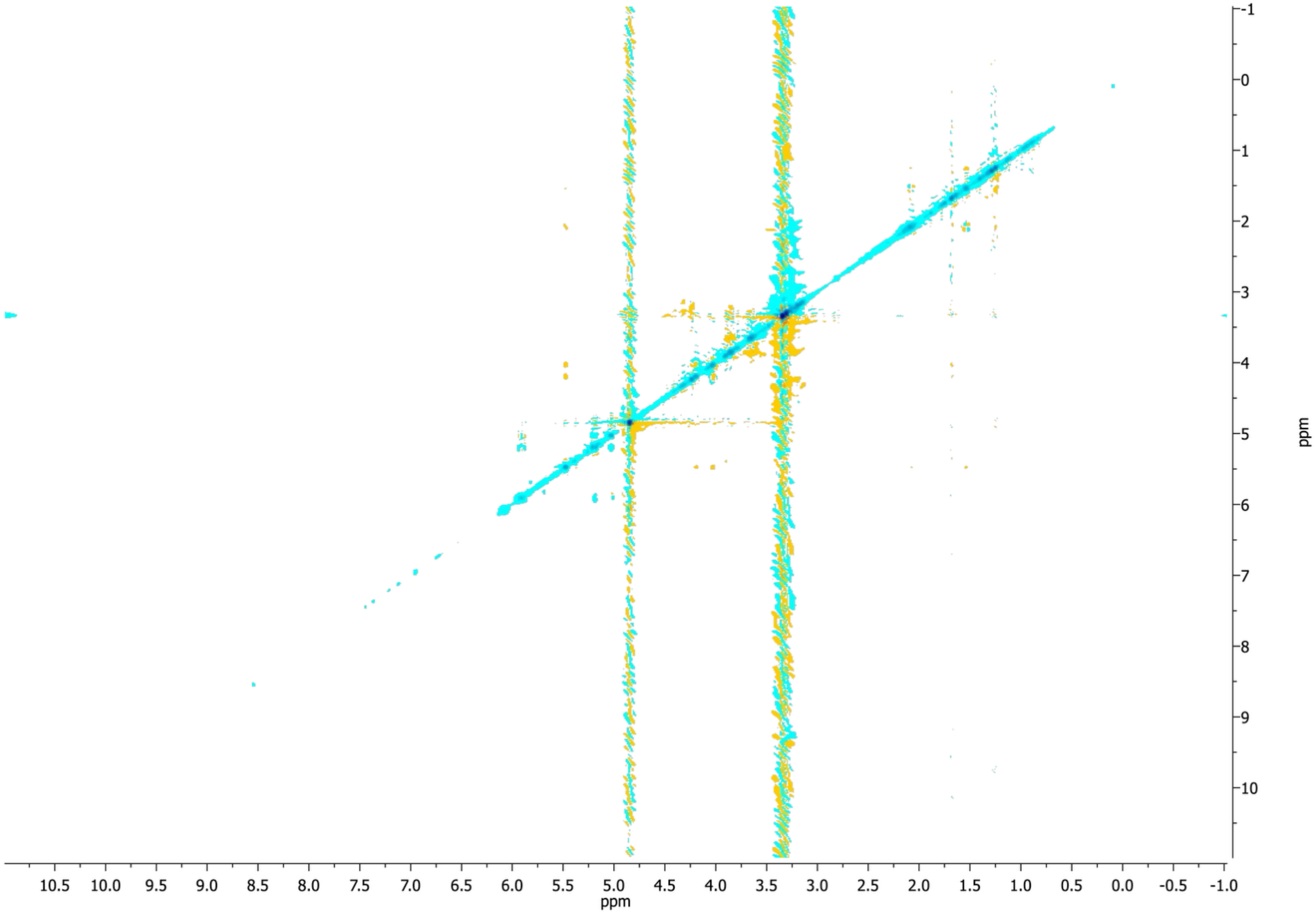

Figure S28. 2D Rotating-frame Overhauser Effect Spectroscopy Spectrum (ROESY) of the Compound **4**.

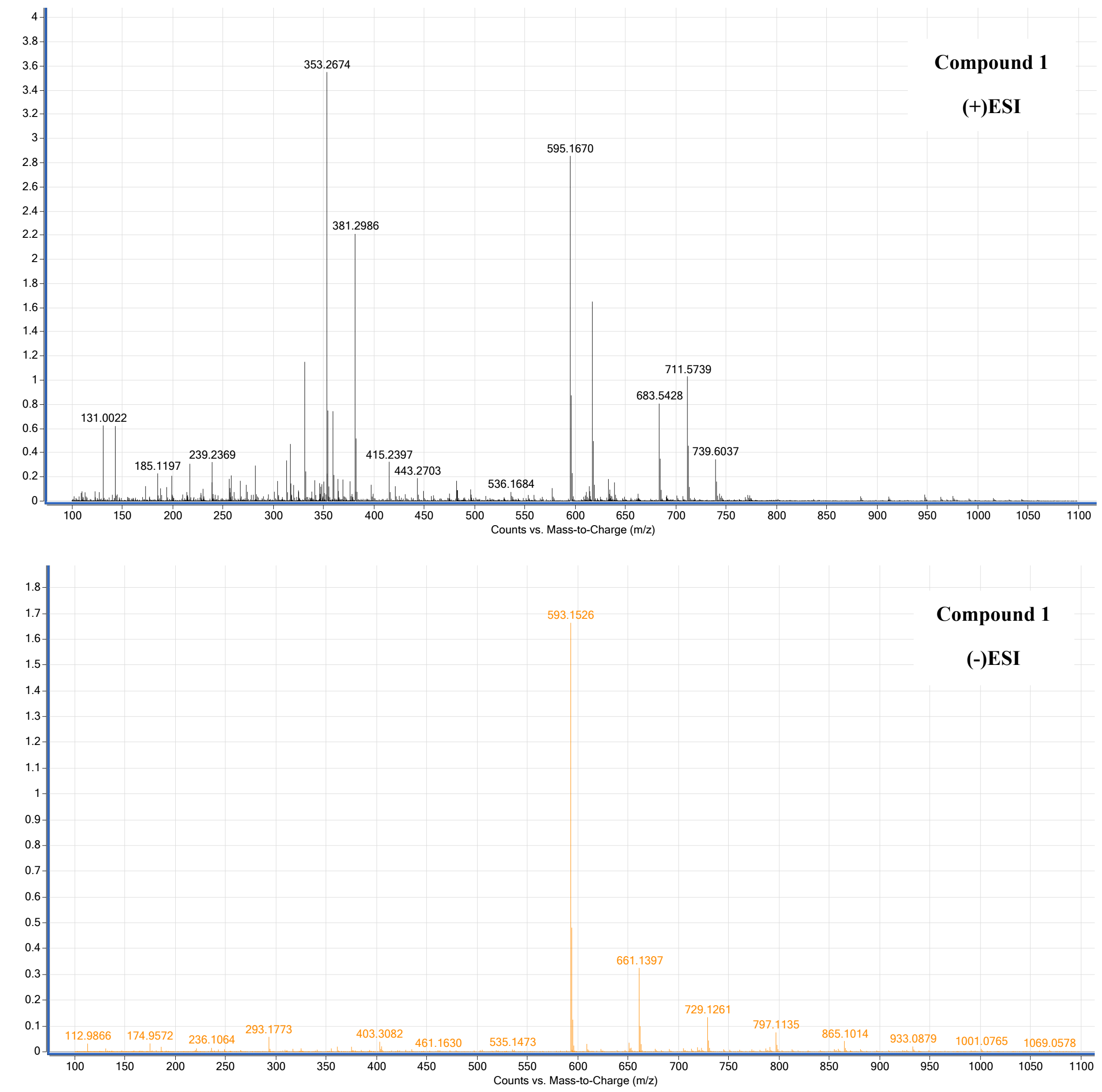

Figure S29. HR-ESI-MS data of Compound **1**.

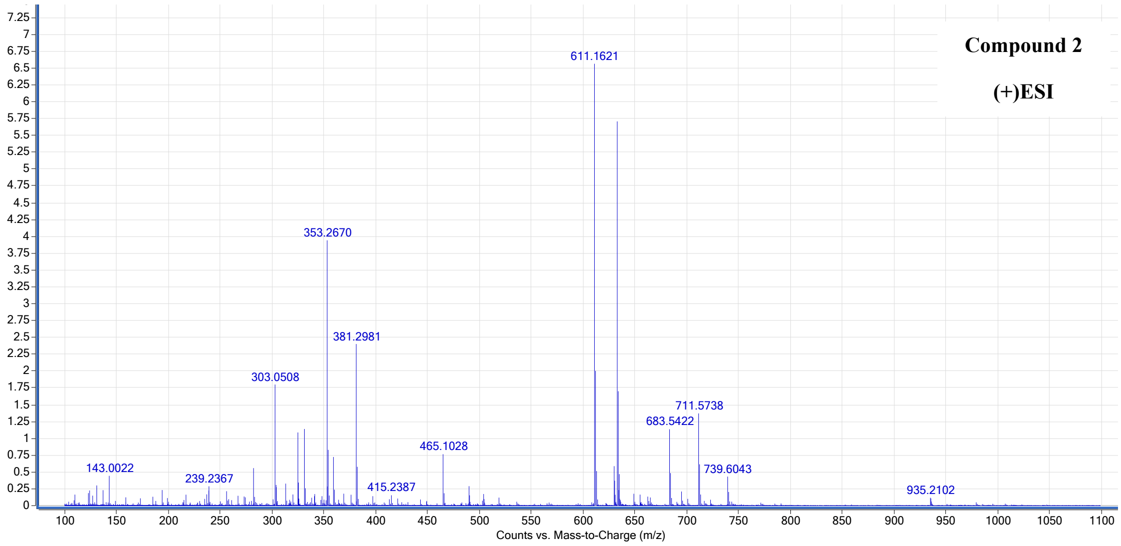

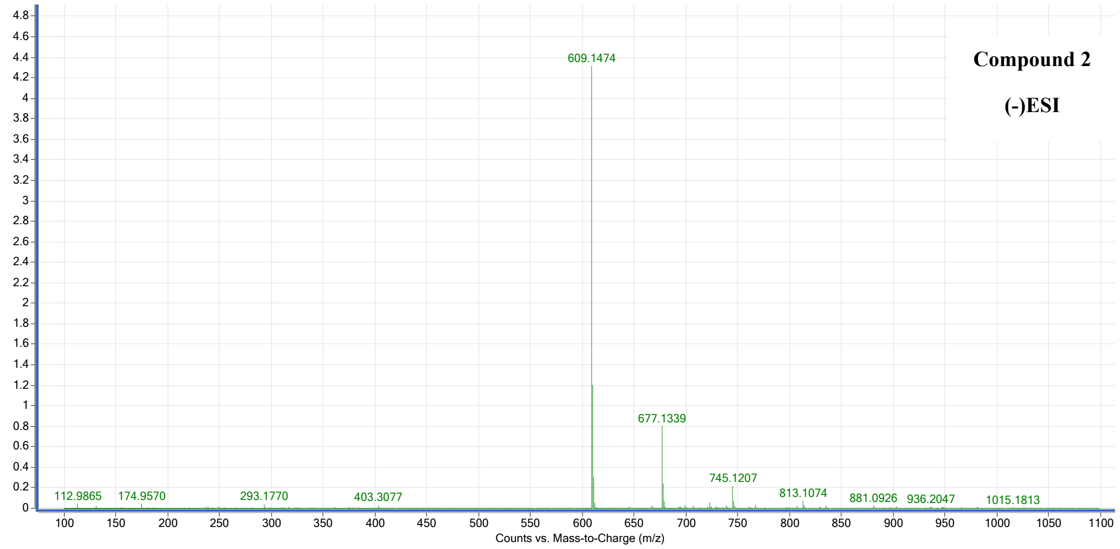

Figure S30. HR-ESI-MS data of Compound **2**.

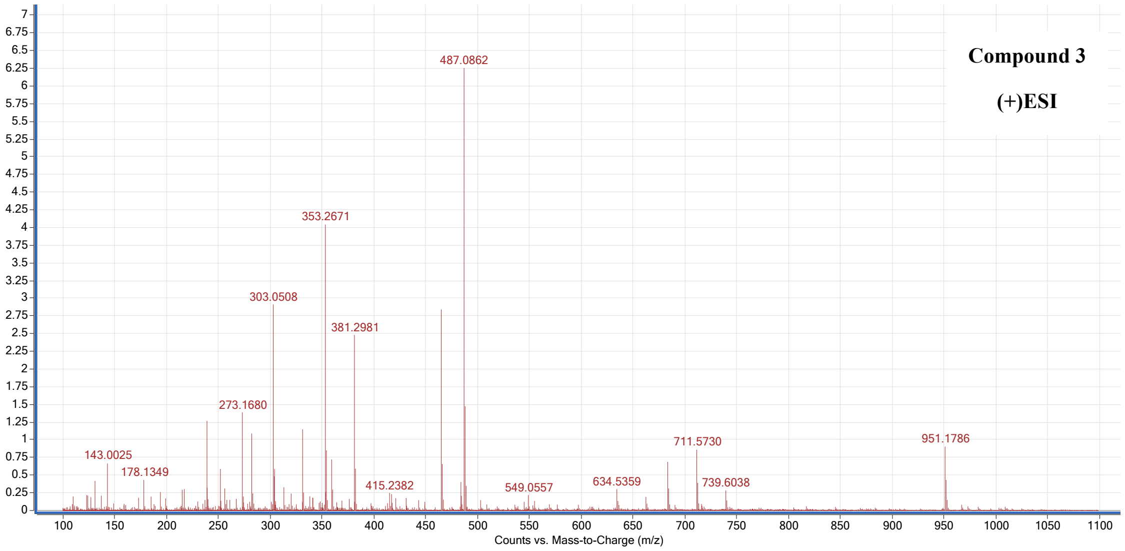

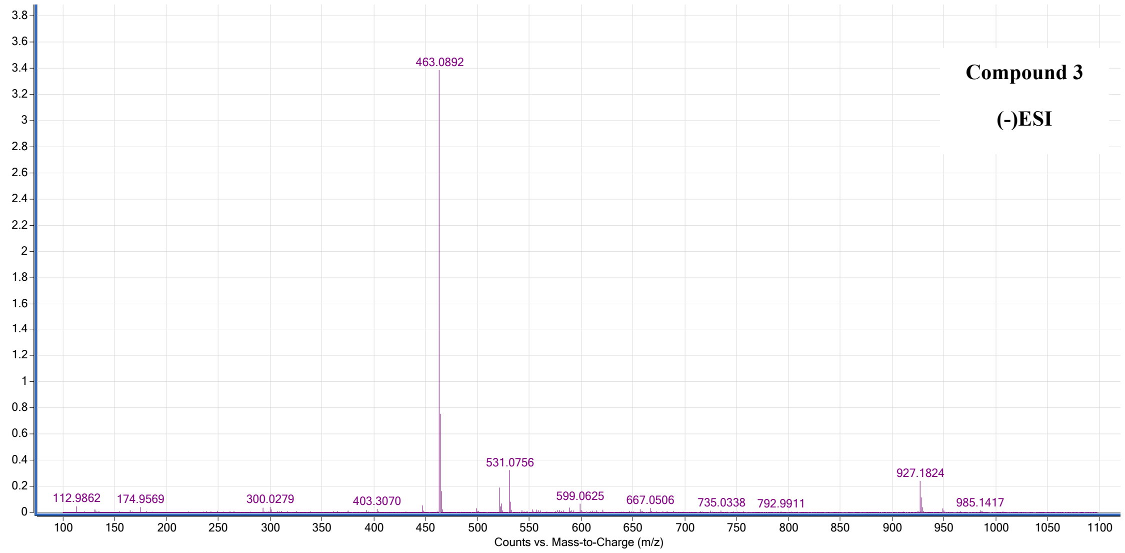

Figure S31. HR-ESI-MS data of Compound **2**.

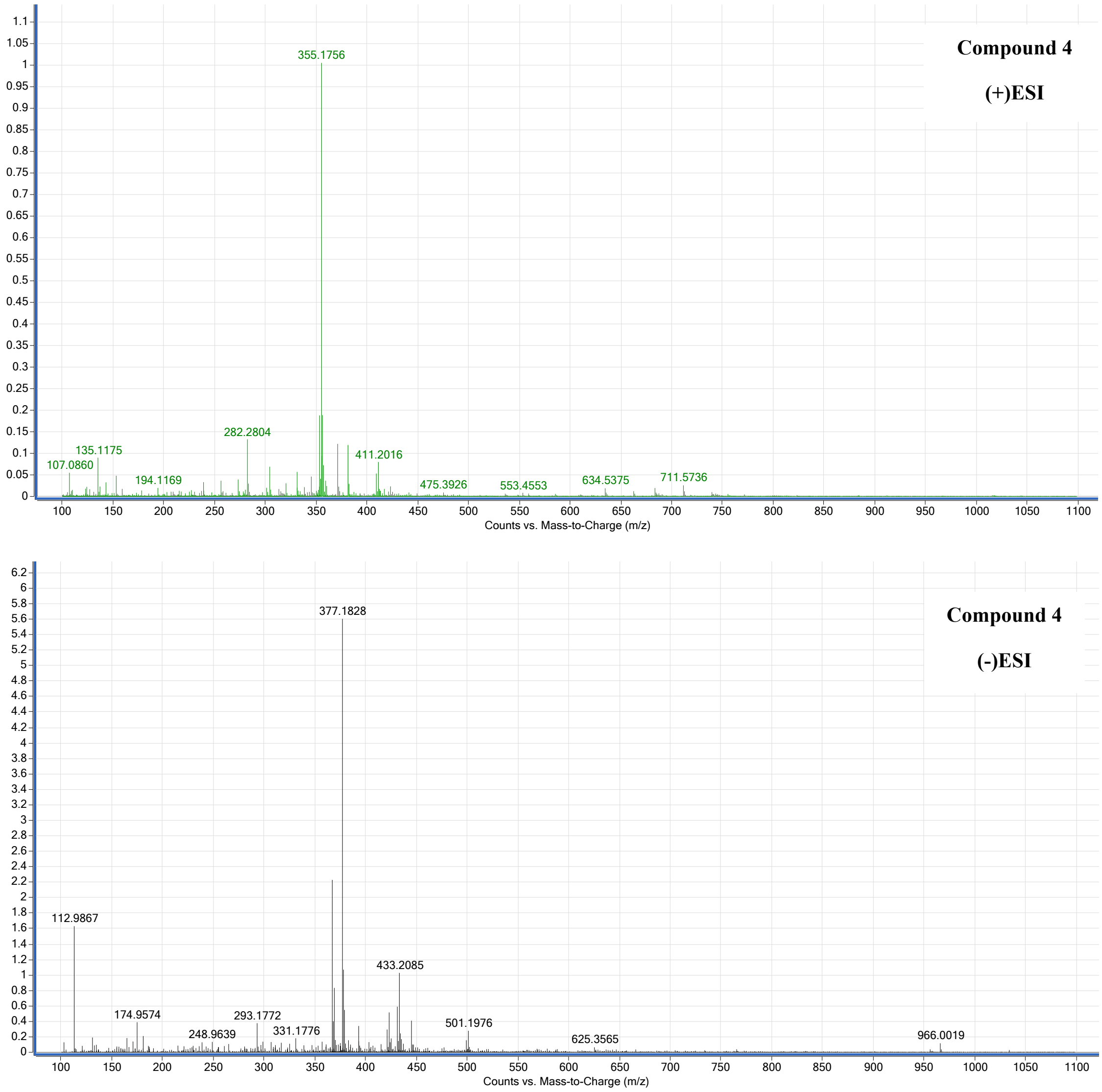

Figure S32. HR-ESI-MS data of Compound **2**.
