# Supplementary material for: Phytochemical Analysis and Bioevaluation of Echinophora sibthorpiana Guss. (Apiaceae): Antioxidant Capacity, DNA Protection and In Vivo Genotoxic Safety
Source: Antioxidants (Basel). 2026 Aug 13;15(8):1009. doi: 10.3390/antiox15081009 (PMC13509921; doi:10.3390/antiox15081009)
Supplement: Supplementary file 1 [file antioxidants-15-01009-s001.zip › Supplementary File S2.pdf]

Supplementary File S2: LC/QTOF-MS-based extracted ion chromatogram (EIC) analysis of target compounds in methanolic extracts of six Turkish *Echinophora* species.

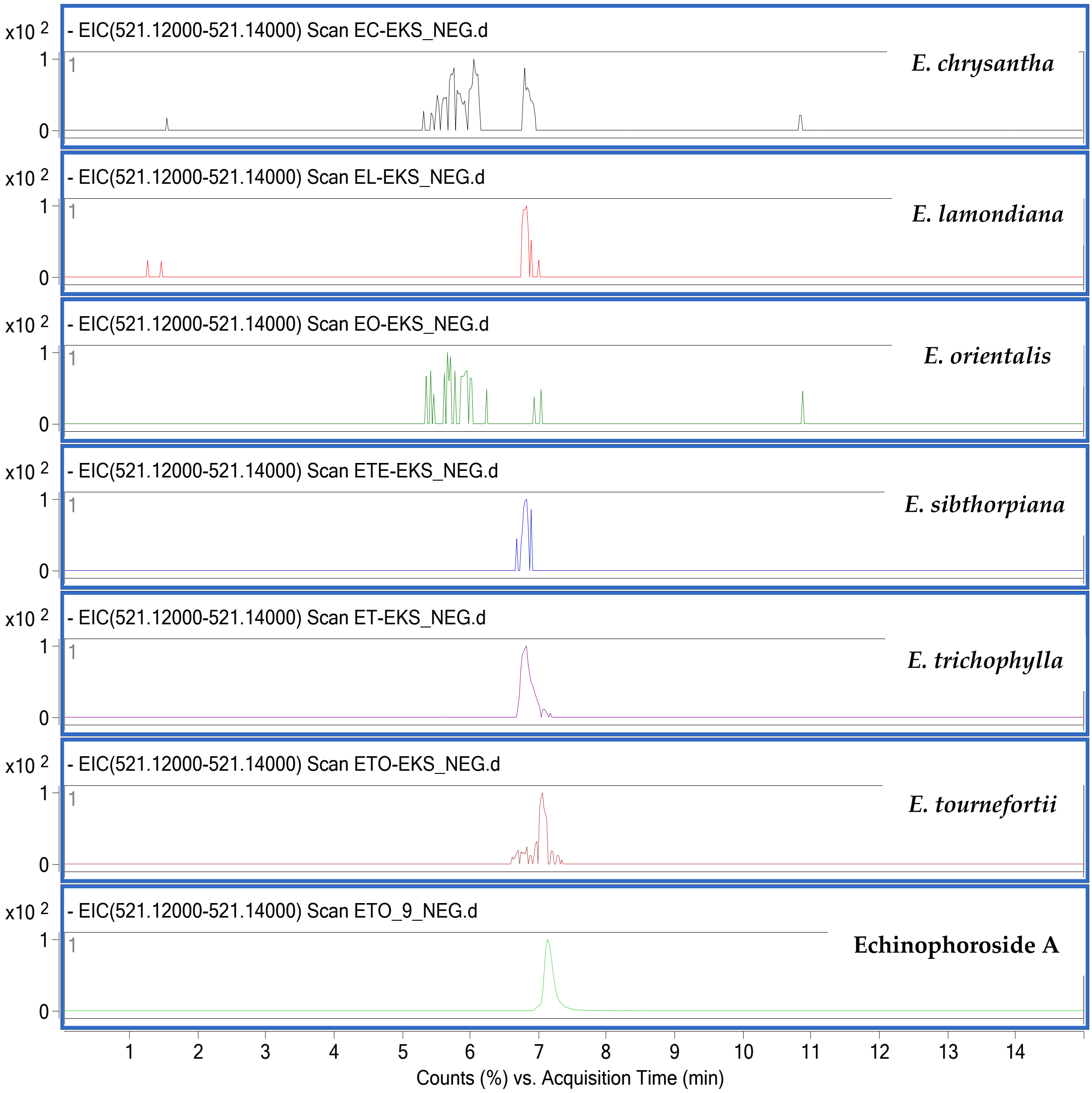

Figure S33. The distribution of the Echinophoroside A in *Echinophora* species.

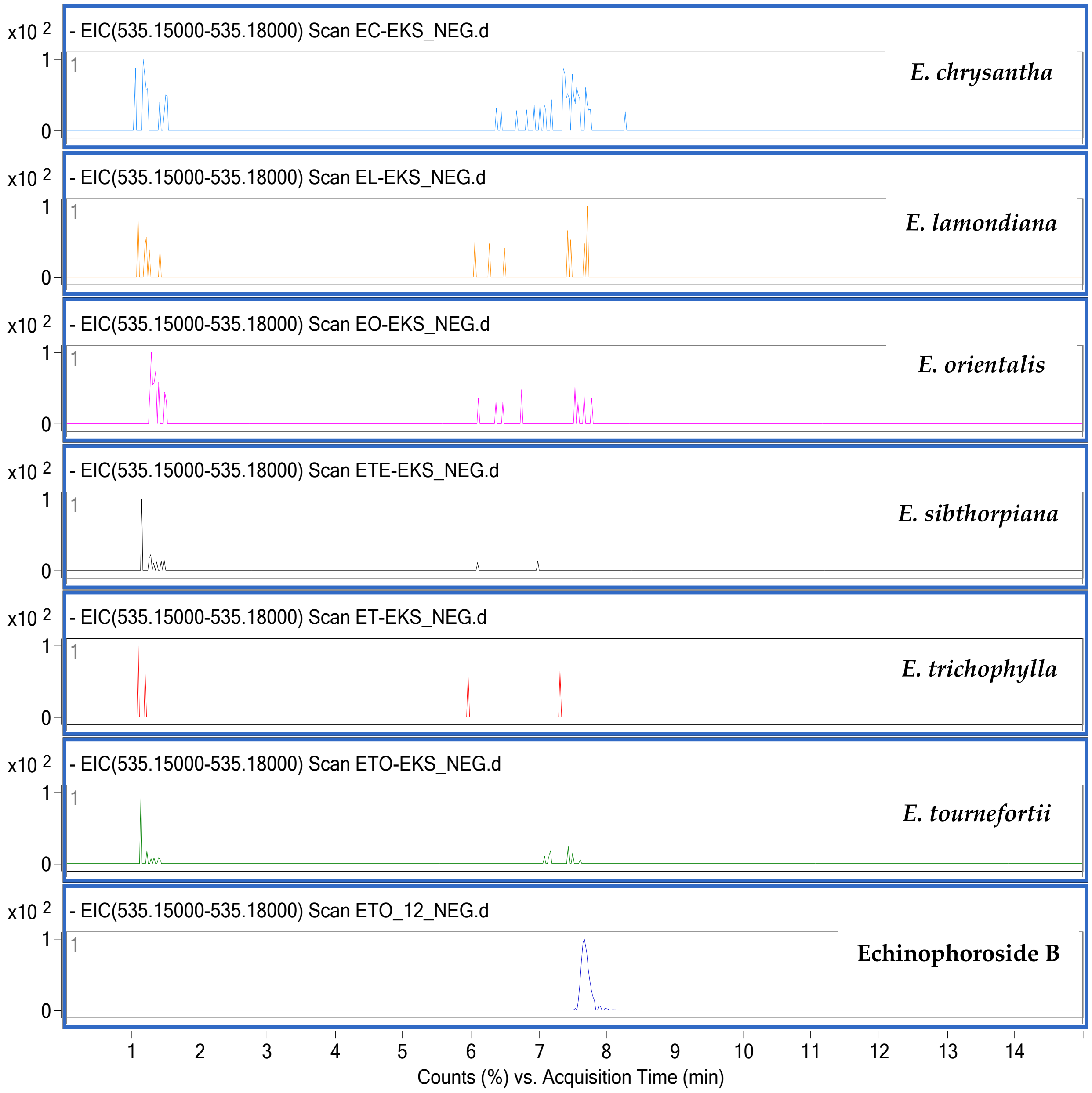

Figure S34. The distribution of the Echinophoroside B in *Echinophora* species.

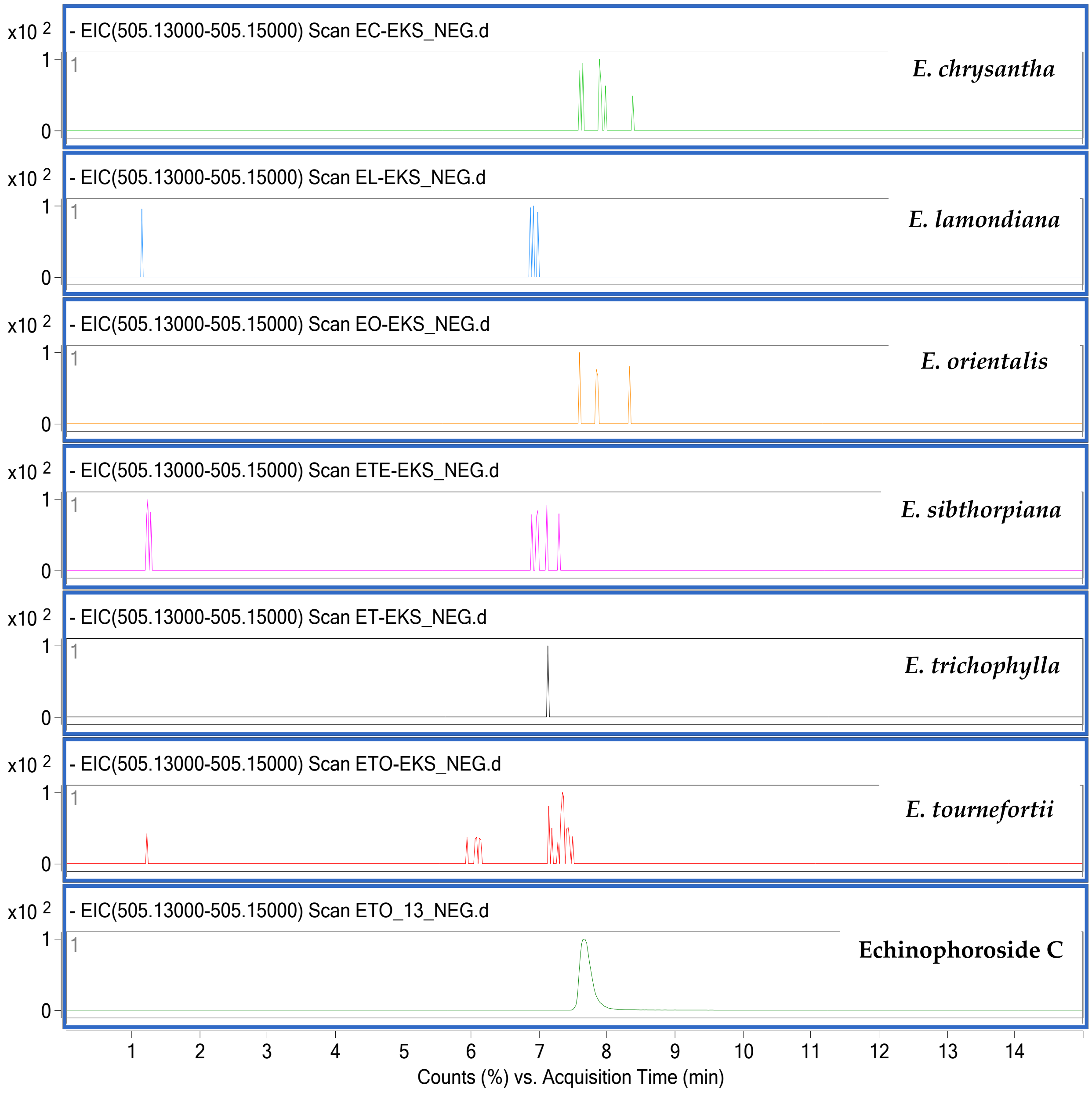

Figure S35. The distribution of the Echinophoroside C in *Echinophora* species.

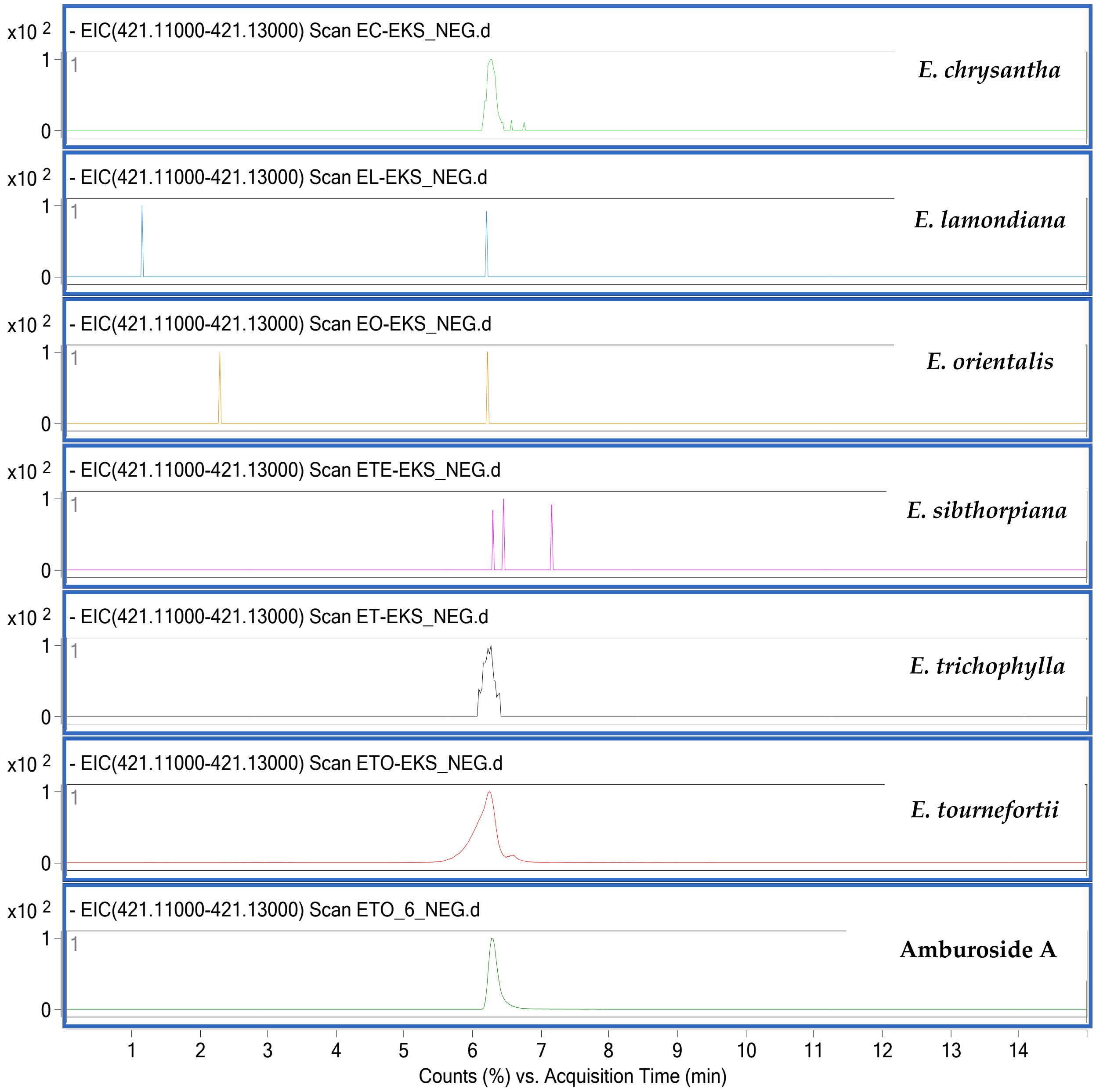

Figure S36. The distribution of the Amburoside A in *Echinophora* species.

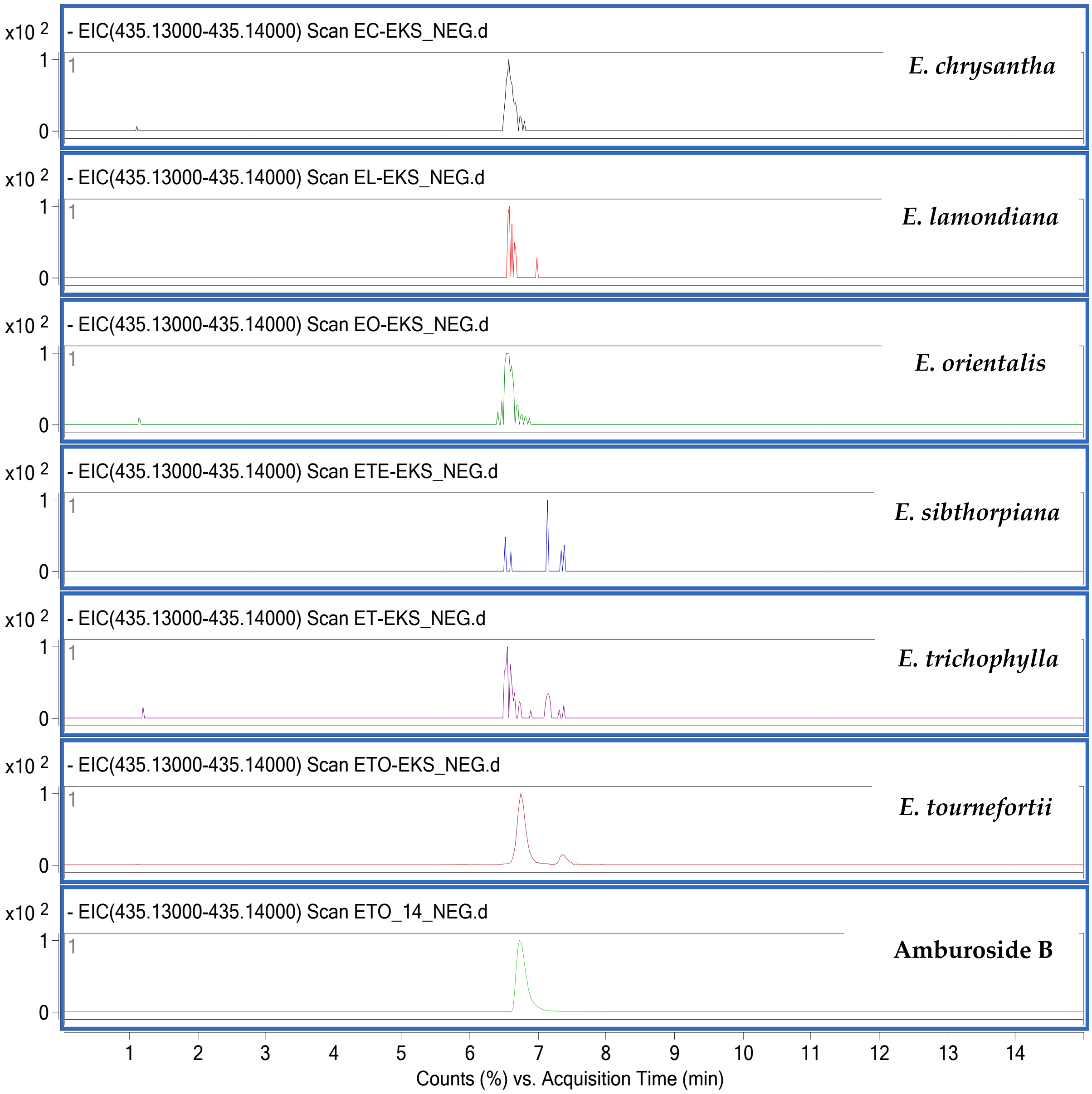

Figure S37. The distribution of the Amburoside B in *Echinophora* species.

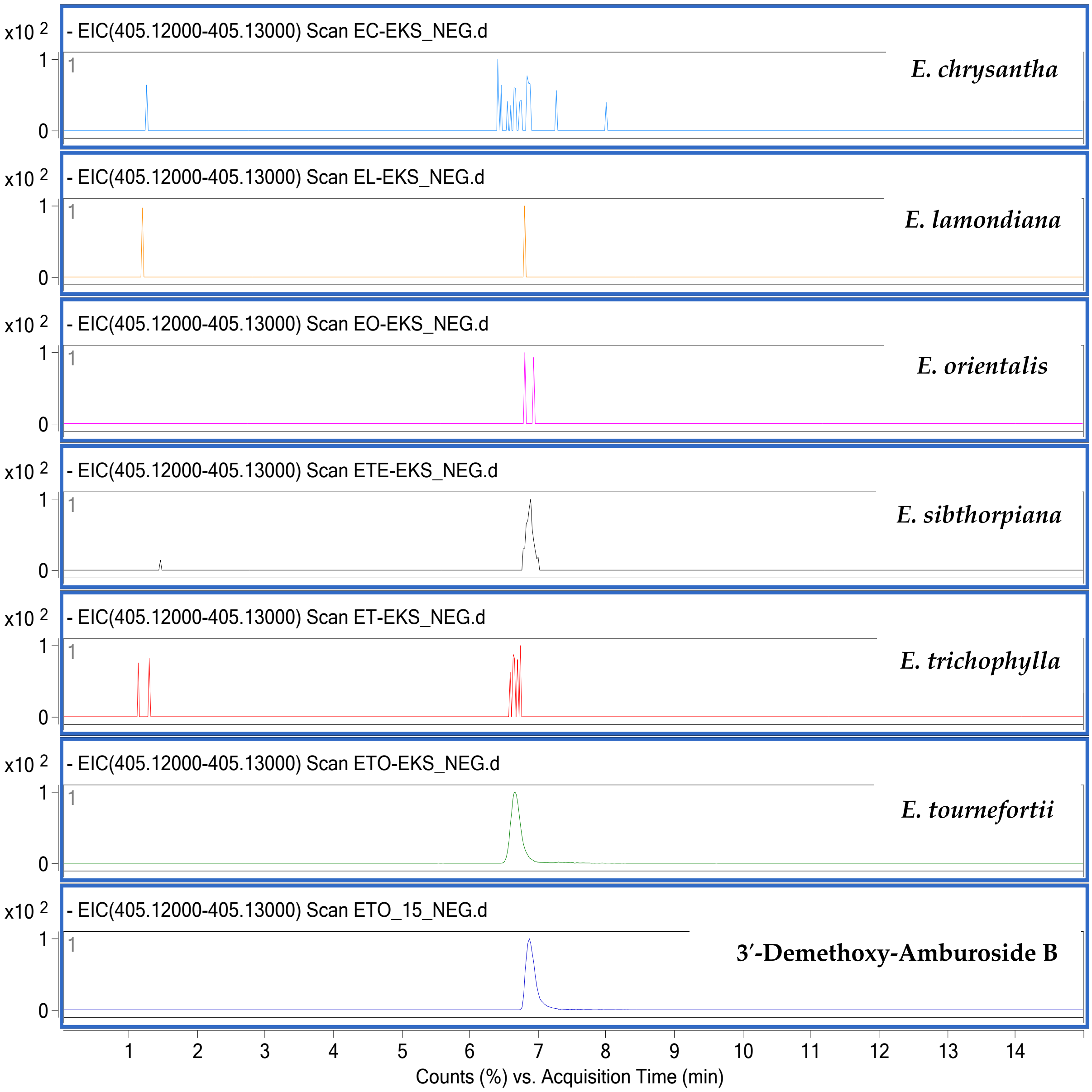

Figure S38. The distribution of the 3′-Demethoxy-Amburoside B in *Echinophora* species.

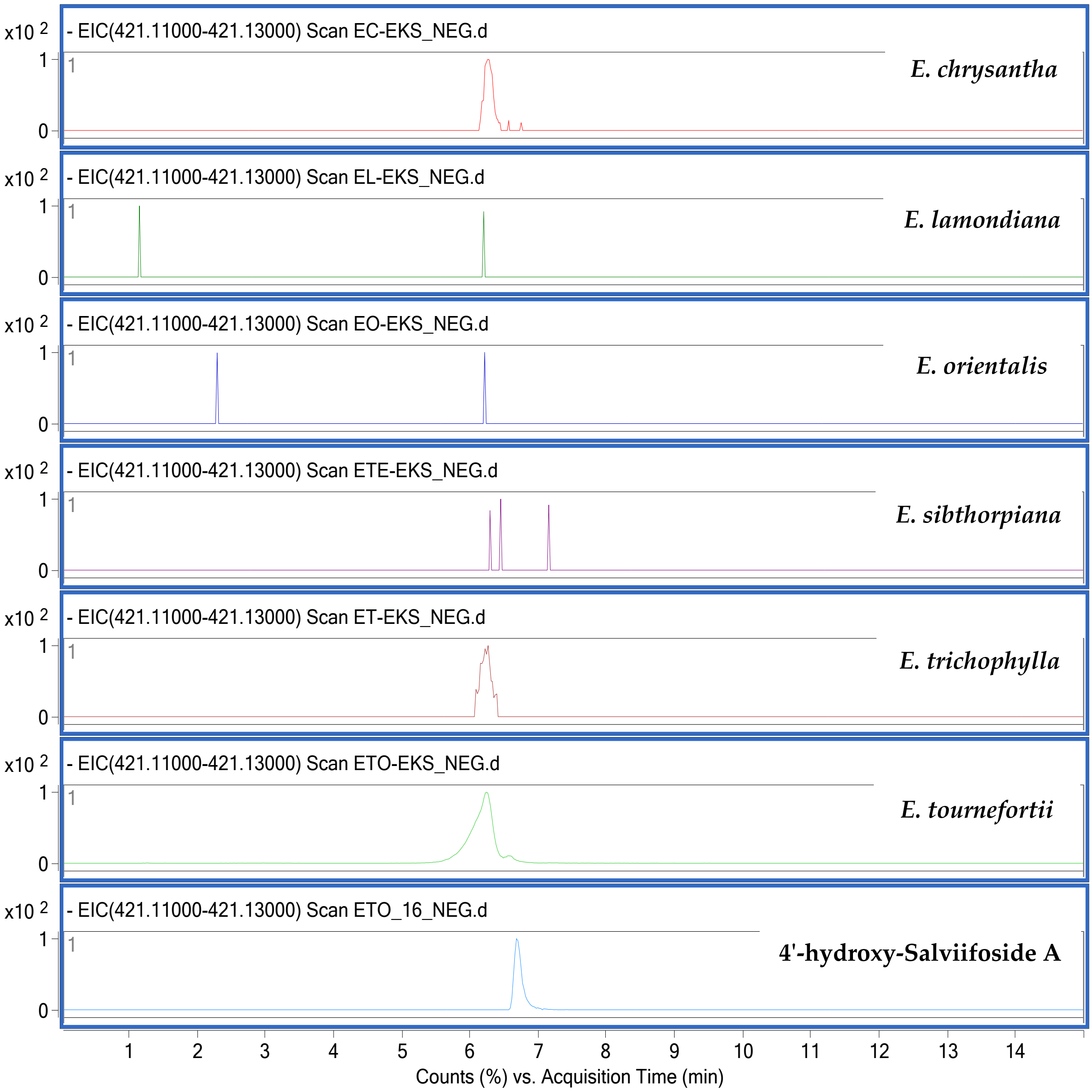

Figure S39. The distribution of the 4'-hydroxy-Salviifoside A in *Echinophora* species.

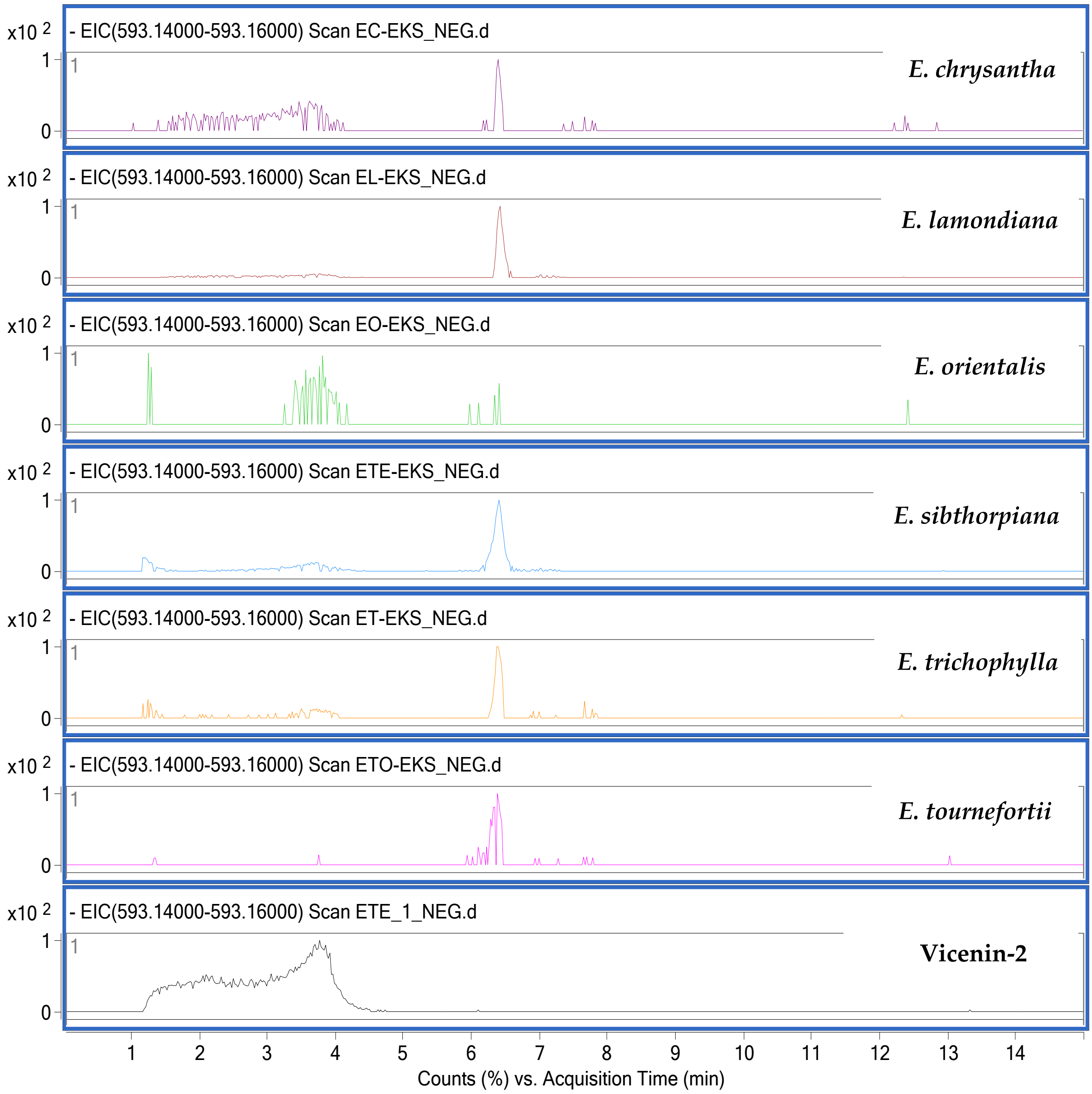

Figure S40. The distribution of the compound **1** (Vicenin-2) in *Echinophora* species.

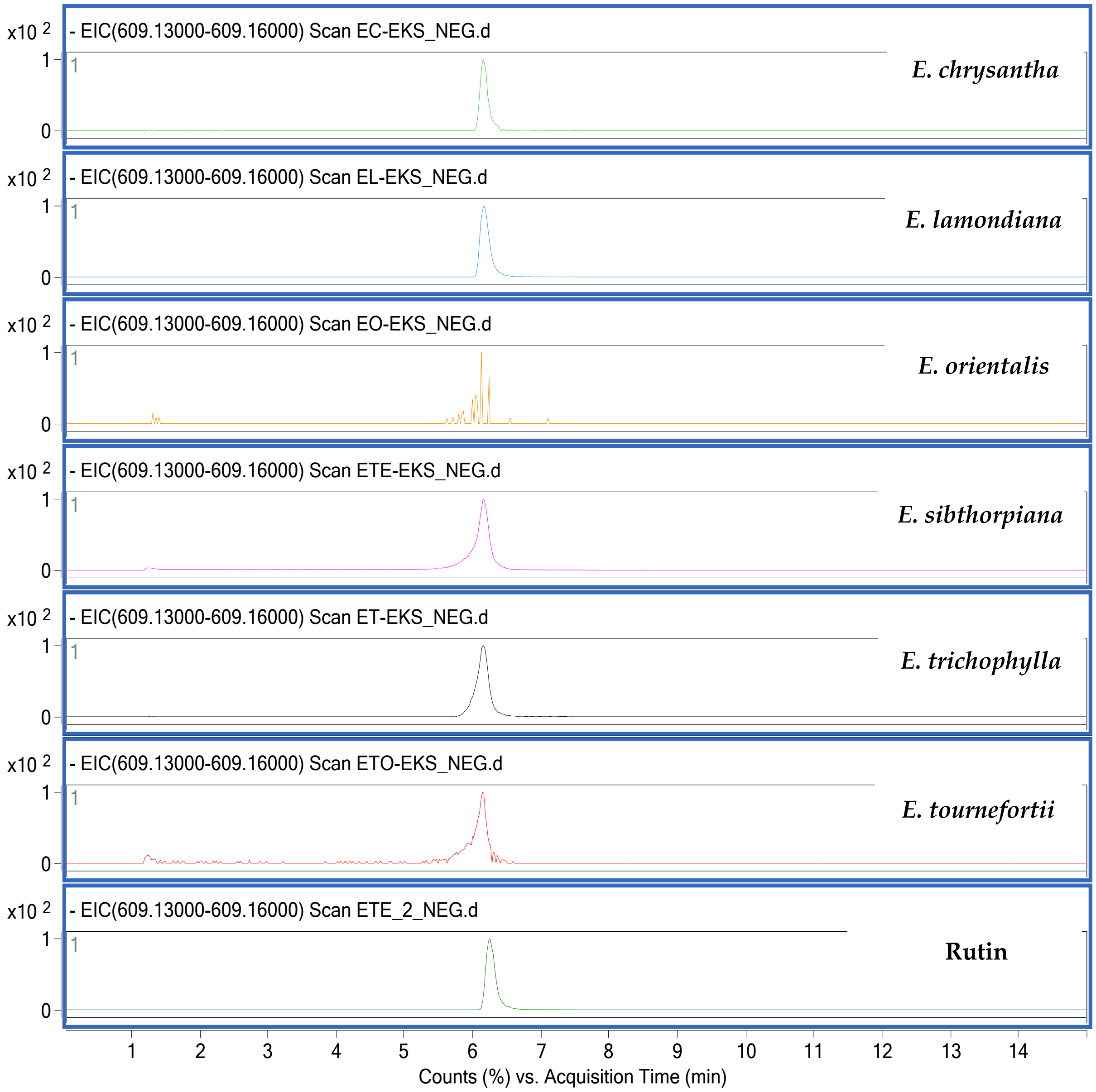

Figure S41. The distribution of the compound **2** (Rutin) in *Echinophora* species.

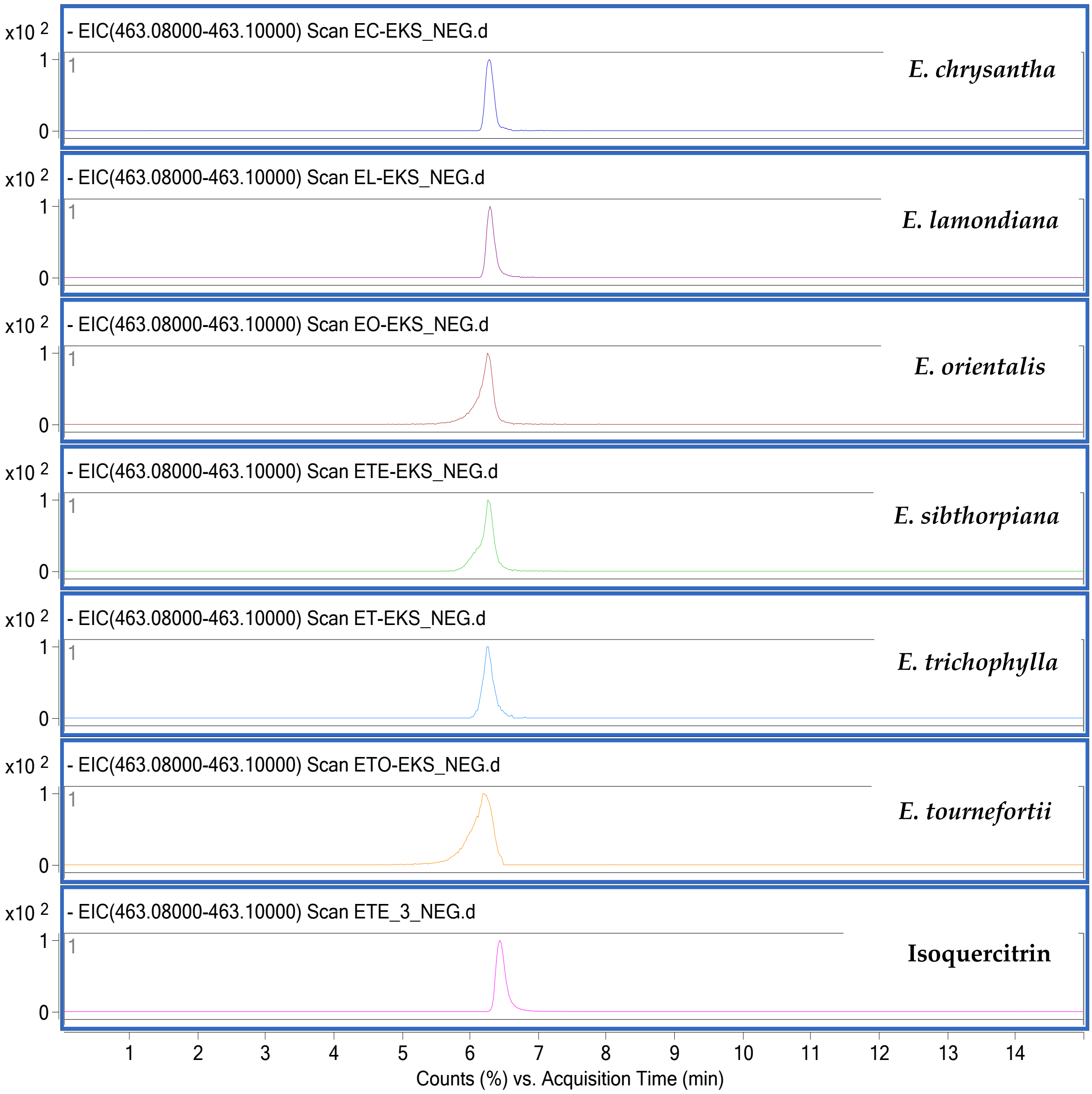

Figure S42. The distribution of the compound **3** (Isoquercitrin) in *Echinophora* species.

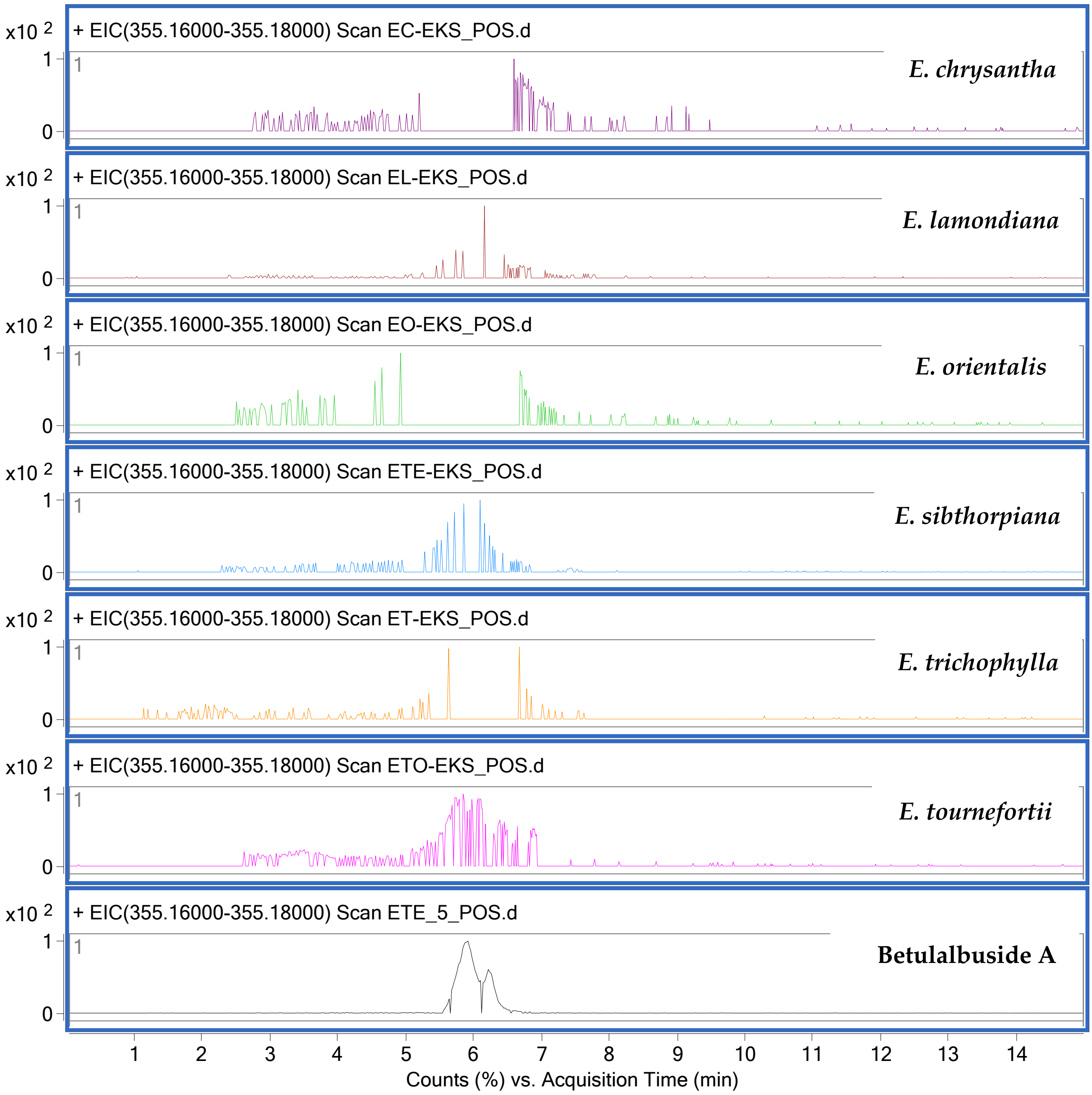

Figure S43. The distribution of the compound **4** (Betulalbuside A) in *Echinophora* species.
