# Supplementary material for: Phytochemical Analysis and Bioevaluation of Echinophora sibthorpiana Guss. (Apiaceae): Antioxidant Capacity, DNA Protection and In Vivo Genotoxic Safety
Source: Antioxidants (Basel). 2026 Aug 13;15(8):1009. doi: 10.3390/antiox15081009 (PMC13509921; doi:10.3390/antiox15081009)
Supplement: Supplementary file 1 [file antioxidants-15-01009-s001.zip › Supplementary File S3.pdf]

Supplementary File S3: Original uncropped gel electrophoresis images of the plasmid DNA protection assay.

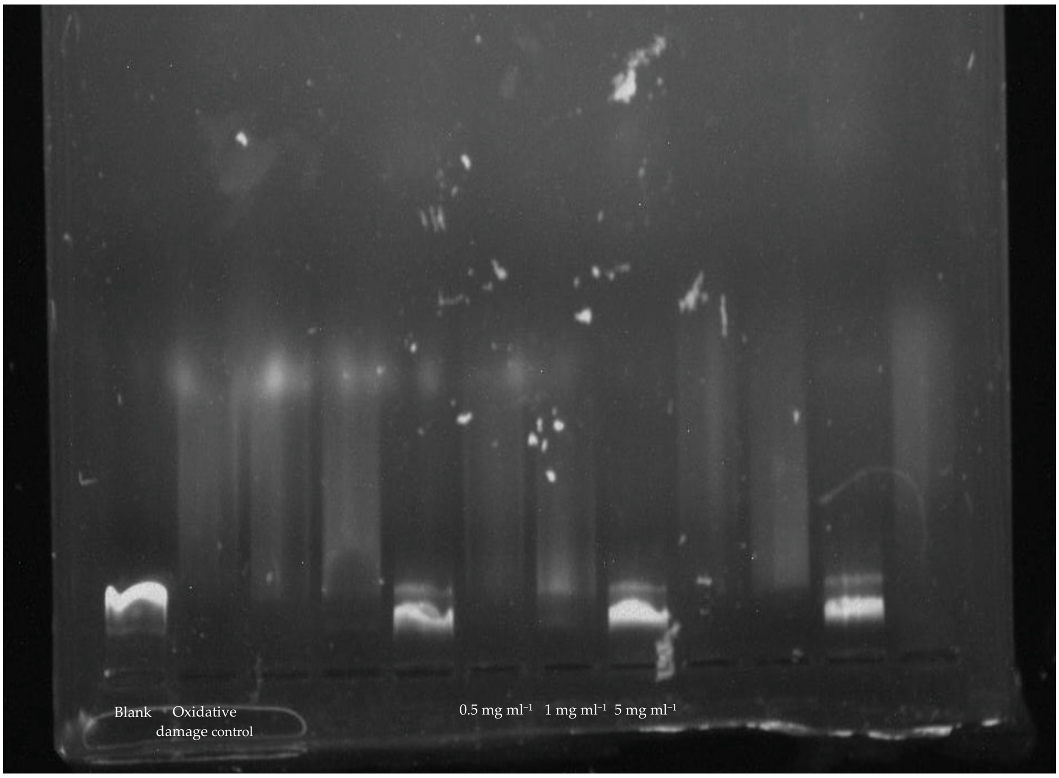

Figure S44. Original uncropped gel image corresponding to Figure 6. All lanes and loading wells are shown, including the lanes containing samples unrelated to the present study that were removed during figure assembly. No adjustments to brightness, contrast, or orientation were made.

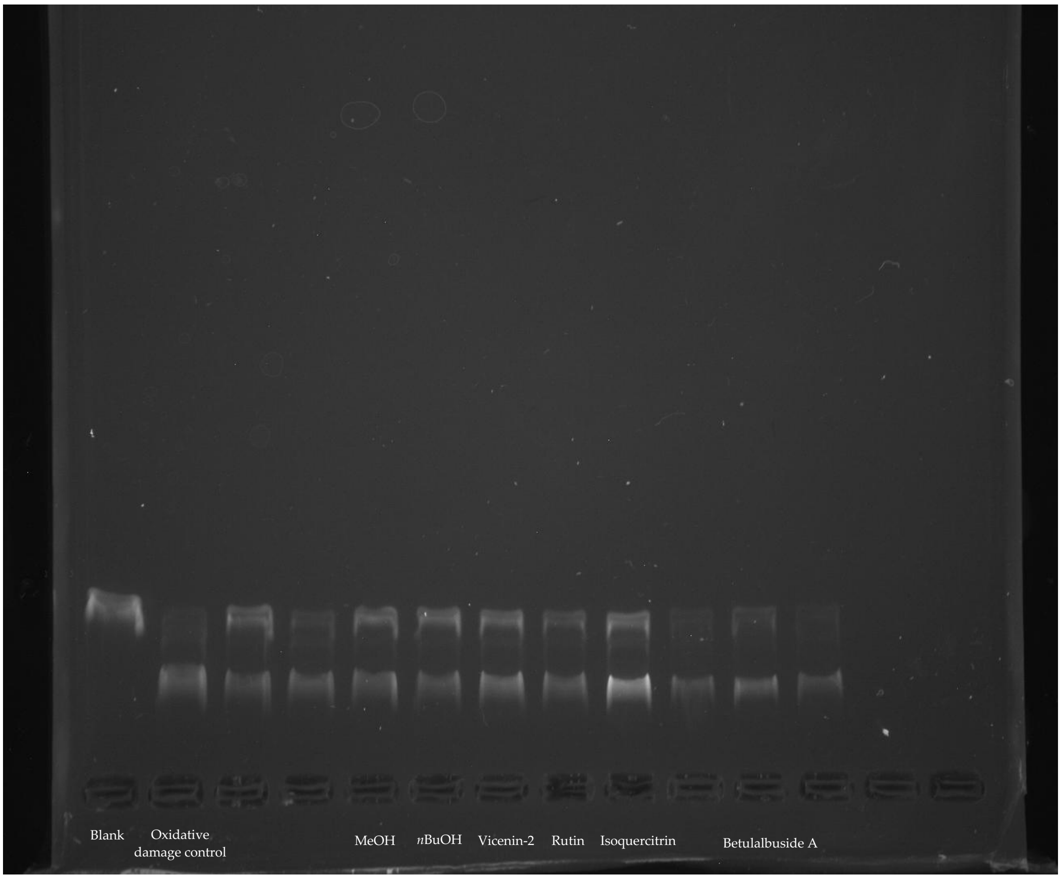

Figure S45. Original uncropped gel image corresponding to Figure 7. All lanes and loading wells are shown, including the lanes containing samples unrelated to the present study that were removed during figure assembly. No adjustments to brightness, contrast, or orientation were made.
